# Supplementary material for: Isolation of Scalarane-Type Sesterterpenoids from the Marine Sponge Dysidea sp. and Stereochemical Reassignment of 12-epi-Phyllactone D/E
Source: Mar Drugs. 2021 Nov 9;19(11):627. doi: 10.3390/md19110627 (PMC8624410; doi:10.3390/md19110627)
Supplement: Supplementary file 1 [file marinedrugs-19-00627-s001.zip › SI-MD-NMR Spectra-revision.pdf]

Supporting Information

NMR Spectra

Isolation of Scalarane-type Sesterterpenoids from the Marine Sponge *Dysidea* sp. and Stereochemical Reassignment of 12-*epi*-phyllactone D/E

A-Young Shin,[1,2] *Arang Son,*[3] *Changhoon Choi,*[3]

*and Jihoon Lee**[1,2]

[1]Korea Institute of Ocean Science and Technology (KIOST), Busan 49111, Republic of Korea

[2]Department of Marine Biotechnology, University of Science and Technology (UST), Daejeon 34113, Republic of Korea

[3]Department of Radiation Oncology, Samsung Medical Center, Seoul 06351, Republic of Korea

Table of contents

Figure S-1. ^{1}H NMR spectrum of compound **1** ($CDCl_3$, 600 MHz).......... 6
Figure S-2. ^{13}C NMR spectrum of compound **1** ($CDCl_3$, 150 MHz).......... 6
Figure S-3. HSQC spectrum of compound **1** ($CDCl_3$, 600 MHz). 7
Figure S-4. ^{1}H-^{1}H COSY spectrum of compound **1** ($CDCl_3$, 600 MHz).......... 7
Figure S-5. HMBC spectrum of compound **1** ($CDCl_3$, 600 MHz). 8
Figure S-6. NOESY spectrum of compound **1** ($CDCl_3$, 600 MHz).......... 8
Figure S-7. HRMS spectrum of compound **1**. 8
Figure S-8. ^{1}H NMR spectrum of compound **2** ($CDCl_3$, 600 MHz).......... 9
Figure S-9. ^{13}C NMR spectrum of compound **2** ($CDCl_3$, 150 MHz).......... 9
Figure S-10. HSQC spectrum of compound **2** ($CDCl_3$, 600 MHz). 10
Figure S-11. ^{1}H-^{1}H COSY spectrum of compound **2** ($CDCl_3$, 600 MHz).......... 10
Figure S-12. HMBC spectrum of compound **2** ($CDCl_3$, 600 MHz). 11
Figure S-13. NOESY spectrum of compound **2** ($CDCl_3$, 600 MHz).......... 11
Figure S-14. HRMS spectrum of compound **2**. 11
Figure S-15. ^{1}H NMR spectrum of compound **3** ($CDCl_3$, 600 MHz).......... 12
Figure S-16. ^{13}C NMR spectrum of compound **3** ($CDCl_3$, 150 MHz).......... 12
Figure S-17. HSQC spectrum of compound **3** ($CDCl_3$, 600 MHz). 13
Figure S-18. ^{1}H-^{1}H COSY spectrum of compound **3** ($CDCl_3$, 600 MHz).......... 13
Figure S-19. HMBC spectrum of compound **3** ($CDCl_3$, 600 MHz). 14
Figure S-20. NOESY spectrum of compound **3** ($CDCl_3$, 600 MHz).......... 14
Figure S-21. HRMS spectrum of compound **3**. 14
Figure S-22. ^{1}H NMR spectrum of compound **4** ($CDCl_3$, 600 MHz).......... 15
Figure S-23. ^{13}C NMR spectrum of compound **4** ($CDCl_3$, 150 MHz).......... 15
Figure S-24. HSQC spectrum of compound **4** ($CDCl_3$, 600 MHz). 16
Figure S-25. ^{1}H-^{1}H COSY spectrum of compound **4** ($CDCl_3$, 600 MHz).......... 16
Figure S-26. HMBC spectrum of compound **4** ($CDCl_3$, 600 MHz). 17
Figure S-27. NOESY spectrum of compound **4** ($CDCl_3$, 600 MHz).......... 17
Figure S-28. HRMS spectrum of compound **4**. 17
Figure S-29. ^{1}H NMR spectrum of compound **5** ($CDCl_3$, 600 MHz).......... 18
Figure S-30. ^{13}C NMR spectrum of compound **5** ($CDCl_3$, 150 MHz).......... 18
Figure S-31. HSQC spectrum of compound **5** ($CDCl_3$, 600 MHz). 19
Figure S-32. ^{1}H-^{1}H COSY spectrum of compound **5** ($CDCl_3$, 600 MHz).......... 19
Figure S-33. HMBC spectrum of compound **5** ($CDCl_3$, 600 MHz). 20
Figure S-34. NOESY spectrum of compound **5** ($CDCl_3$, 600 MHz).......... 20
Figure S-35. HRMS spectrum of compound **5**. 20

Figure S-36. ^{1}H NMR spectrum of compound **6** ($CDCl_3$, 600 MHz)........ 21
Figure S-37. ^{13}C NMR spectrum of compound **6** ($CDCl_3$, 150 MHz)........ 21
Figure S-38. HSQC spectrum of compound **6** ($CDCl_3$, 600 MHz). 22
Figure S-39. ^{1}H-^{1}H COSY spectrum of compound **6** ($CDCl_3$, 600 MHz)........ 22
Figure S-40. HMBC spectrum of compound **6** ($CDCl_3$, 600 MHz). 23
Figure S-41. NOESY spectrum of compound **6** ($CDCl_3$, 600 MHz)........ 23
Figure S-42. HRMS spectrum of compound **6**. 23
Figure S-43. ^{1}H NMR spectrum of compound **7** ($CDCl_3$, 600 MHz)........ 24
Figure S-44. ^{13}C NMR spectrum of compound **7** ($CDCl_3$, 150 MHz)........ 24
Figure S-45. HSQC spectrum of compound **7** ($CDCl_3$, 600 MHz). 25
Figure S-46. ^{1}H-^{1}H COSY spectrum of compound **7** ($CDCl_3$, 600 MHz)........ 25
Figure S-47. HMBC spectrum of compound **7** ($CDCl_3$, 600 MHz). 26
Figure S-48. NOESY spectrum of compound **7** ($CDCl_3$, 600 MHz)........ 26
Figure S-49. HRMS spectrum of compound **7**. 26
Figure S-50. ^{1}H NMR spectrum of compound **8** ($CDCl_3$, 600 MHz)........ 27
Figure S-51. ^{13}C NMR spectrum of compound **8** ($CDCl_3$, 150 MHz)........ 27
Figure S-52. HSQC spectrum of compound **8** ($CDCl_3$, 600 MHz). 28
Figure S-53. ^{1}H-^{1}H COSY spectrum of compound **8** ($CDCl_3$, 600 MHz)........ 28
Figure S-54. HMBC spectrum of compound **8** ($CDCl_3$, 600 MHz). 29
Figure S-55. NOESY spectrum of compound **8** ($CDCl_3$, 600 MHz)........ 29
Figure S-56. HRMS of compound **8**........ 29
Figure S-57. ^{1}H NMR spectrum of compound **9** ($CDCl_3$, 600 MHz)........ 30
Figure S-58. ^{13}C NMR spectrum of compound **9** ($CDCl_3$, 150 MHz)........ 30
Figure S-59. HSQC spectrum of compound **9** ($CDCl_3$, 600 MHz). 31
Figure S-60. ^{1}H-^{1}H COSY spectrum of compound **9** ($CDCl_3$, 600 MHz)........ 31
Figure S-61. HMBC spectrum of compound **9** ($CDCl_3$, 600 MHz). 32
Figure S-62. NOESY spectrum of compound **9** ($CDCl_3$, 600 MHz)........ 32
Figure S-63. HRMS spectrum of compound **9**. 32
Figure S-64. ^{1}H NMR spectrum of compound **10** ($CDCl_3$, 600 MHz)........ 33
Figure S-65. ^{13}C NMR spectrum of compound **10** ($CDCl_3$, 150 MHz)........ 33
Figure S-66. HSQC spectrum of compound **10** ($CDCl_3$, 600 MHz). 34
Figure S-67. ^{1}H-^{1}H COSY spectrum of compound **10** ($CDCl_3$, 600 MHz)........ 34
Figure S-68. HMBC spectrum of compound **10** ($CDCl_3$, 600 MHz). 35
Figure S-69. NOESY spectrum of compound **10** ($CDCl_3$, 600 MHz)........ 35
Figure S-70. HRMS spectrum of compound **10**. 35
Figure S-71. ^{1}H NMR spectrum of compound **11** ($CDCl_3$, 600 MHz)........ 36
Figure S-72. ^{13}C NMR spectrum of compound **11** ($CDCl_3$, 150 MHz)........ 36

Figure S-73. HSQC spectrum of compound **11** ($CDCl_3$, 600 MHz). 37
Figure S-74. ^{1}H-^{1}H COSY spectrum of compound **11** ($CDCl_3$, 600 MHz). 37
Figure S-75. HMBC spectrum of compound **11** ($CDCl_3$, 600 MHz). 38
Figure S-76. NOESY spectrum of compound **11** ($CDCl_3$, 600 MHz). 38
Figure S-77. HRMS spectrum of compound **11**. 38
Figure S-78. ^{1}H NMR spectrum of compound **12** ($CDCl_3$, 600 MHz). 39
Figure S-79. ^{13}C NMR spectrum of compound **12** ($CDCl_3$, 150 MHz). 39
Figure S-80. HSQC spectrum of compound **12** ($CDCl_3$, 600 MHz). 40
Figure S-81. ^{1}H-^{1}H COSY spectrum of compound **12** ($CDCl_3$, 600 MHz). 40
Figure S-82. HMBC spectrum of compound **12** ($CDCl_3$, 600 MHz). 41
Figure S-83. NOESY spectrum of compound **12** ($CDCl_3$, 600 MHz). 41
Figure S-84. HRMS spectrum of compound **12**. 41
Figure S-85. ^{1}H NMR spectrum of compound **13** ($CDCl_3$, 600 MHz). 42
Figure S-86. ^{13}C NMR spectrum of compound **13** ($CDCl_3$, 150 MHz). 42
Figure S-87. HSQC spectrum of compound **13** ($CDCl_3$, 600 MHz). 43
Figure S-88. ^{1}H-^{1}H COSY spectrum of compound **13** ($CDCl_3$, 600 MHz). 43
Figure S-89. HMBC spectrum of compound **13** ($CDCl_3$, 600 MHz). 44
Figure S-90. NOESY spectrum of compound **13** ($CDCl_3$, 600 MHz). 44
Figure S-91. HRMS spectrum of compound **13**. 44
Figure S-92. ^{1}H NMR spectrum of compound **14** ($CDCl_3$, 600 MHz). 45
Figure S-93. ^{13}C NMR spectrum of compound **14** ($CDCl_3$, 150 MHz). 45
Figure S-94. HSQC spectrum of compound **14** ($CDCl_3$, 600 MHz). 46
Figure S-95. ^{1}H-^{1}H COSY spectrum of compound **14** ($CDCl_3$, 600 MHz). 46
Figure S-96. HMBC spectrum of compound **14** ($CDCl_3$, 600 MHz). 47
Figure S-97. NOESY spectrum of compound **14** ($CDCl_3$, 600 MHz). 47
Figure S-98. HRMS spectrum of compound **14**. 47
Figure S-99. ^{1}H NMR spectrum of compound **15** ($CDCl_3$, 600 MHz). 48
Figure S-100. ^{13}C NMR spectrum of compound **15** ($CDCl_3$, 150 MHz). 48
Figure S-101. HSQC spectrum of compound **15** ($CDCl_3$, 600 MHz). 49
Figure S-102. ^{1}H-^{1}H COSY spectrum of compound **15** ($CDCl_3$, 600 MHz). 49
Figure S-103. HMBC spectrum of compound **15** ($CDCl_3$, 600 MHz). 50
Figure S-104. NOESY spectrum of compound **15** ($CDCl_3$, 600 MHz). 50
Figure S-105. HRMS spectrum of compound **15**. 50
Figure S-106. ^{1}H NMR spectrum of compound **16** ($CDCl_3$, 600 MHz). 51
Figure S-107. ^{13}C NMR spectrum of compound **16** ($CDCl_3$, 150 MHz). 51
Figure S-108. HSQC spectrum of compound **16** ($CDCl_3$, 600 MHz). 52
Figure S-109. ^{1}H-^{1}H COSY spectrum of compound **16** ($CDCl_3$, 600 MHz). 52

Figure S-110. HMBC spectrum of compound **16** ($CDCl_3$, 600 MHz). 53
Figure S-111. NOESY spectrum of compound **16** ($CDCl_3$, 600 MHz)... 53
Figure S-112. HRMS spectrum of compound **16**. 53

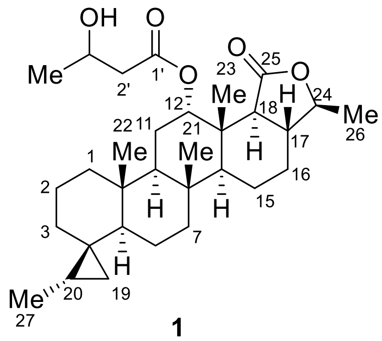

1

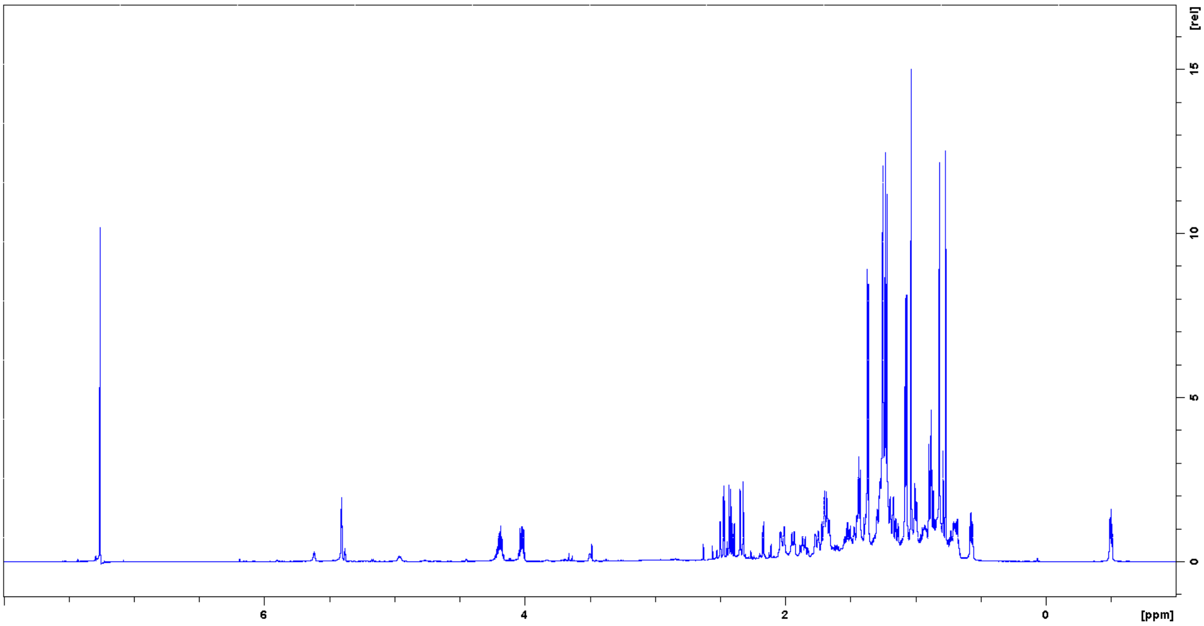

Figure S-1. ^{1}H NMR spectrum of compound **1** ($CDCl_3$, 600 MHz).

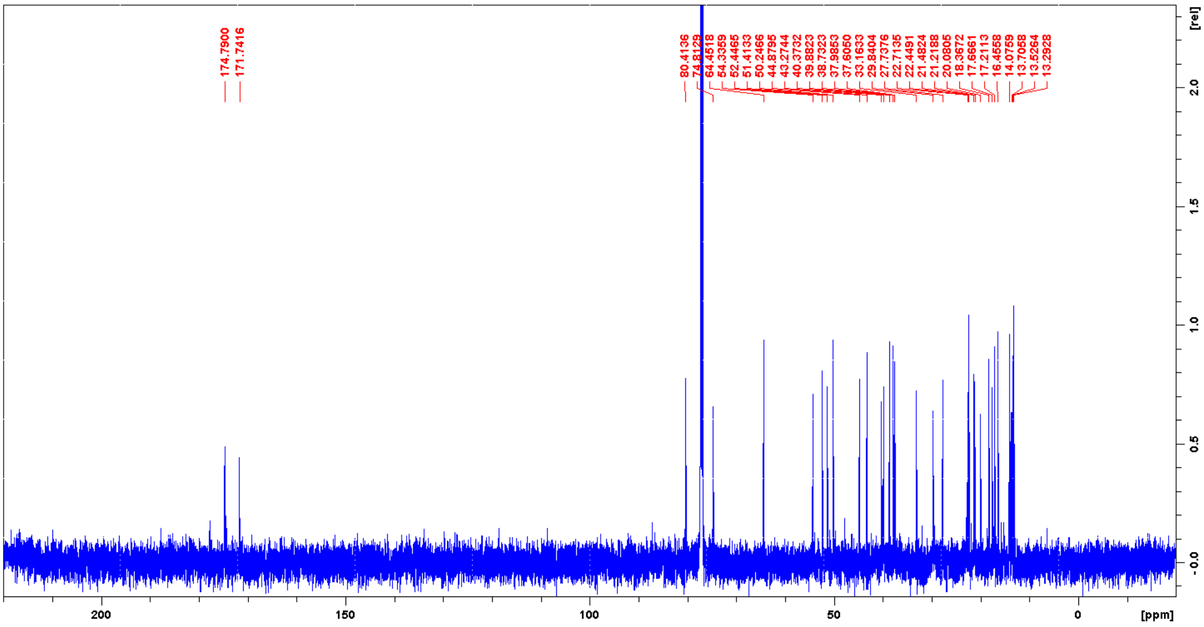

Figure S-2. ^{13}C NMR spectrum of compound **1** ($CDCl_3$, 150 MHz).

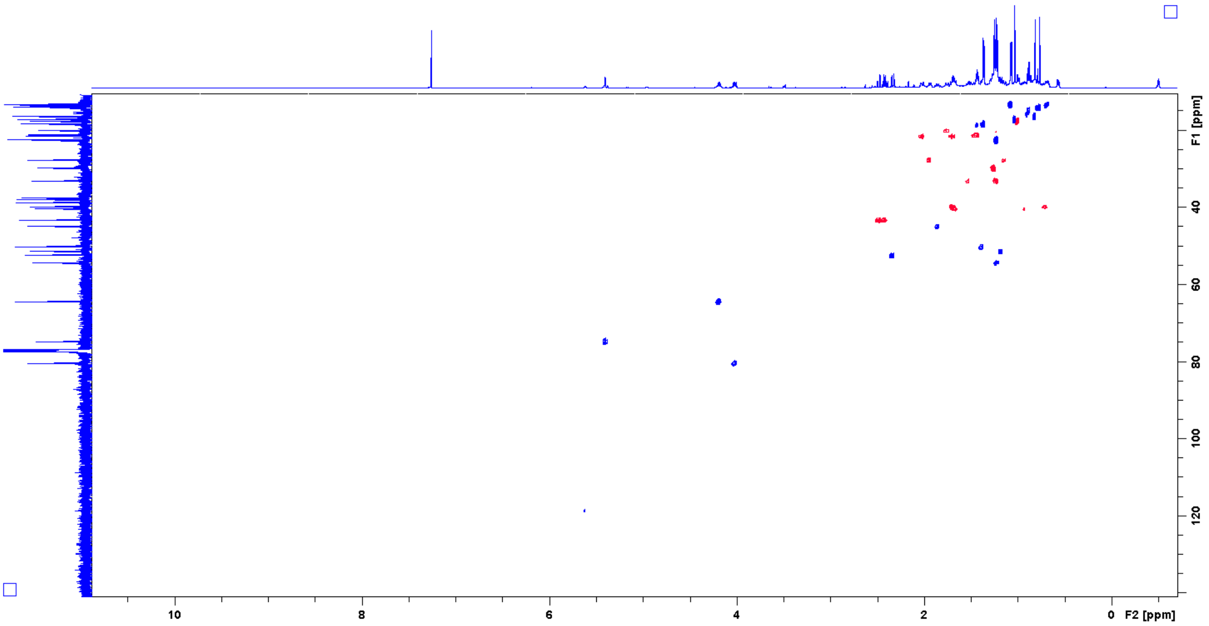

Figure S-3. HSQC spectrum of compound **1** ($CDCl_3$, 600 MHz).

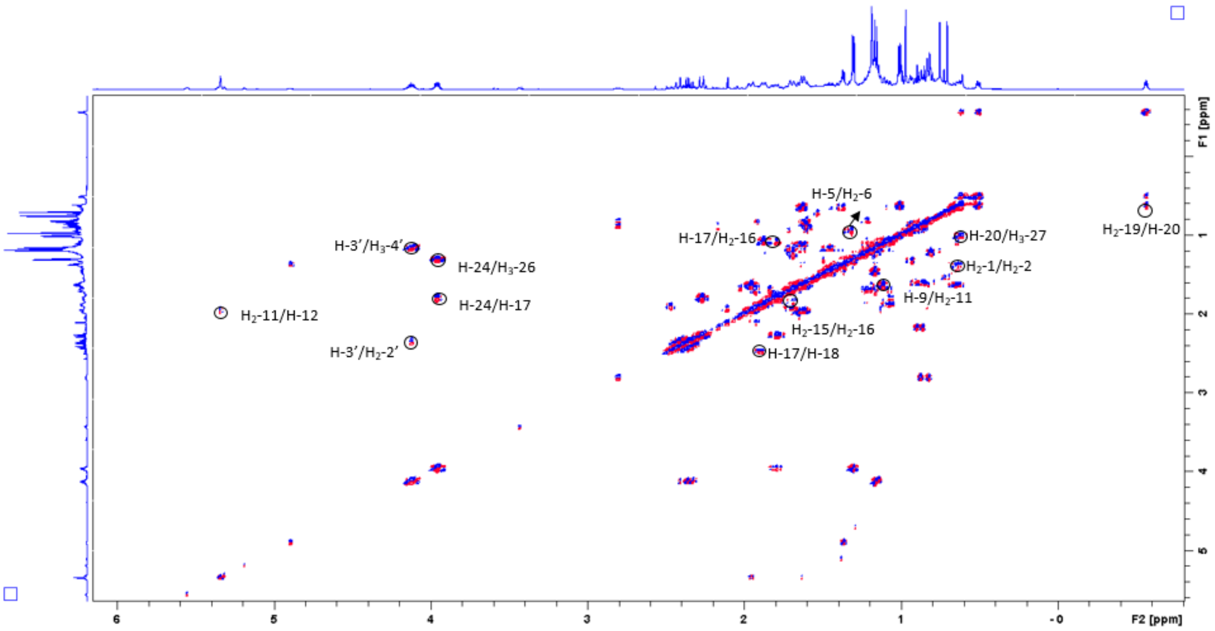

Figure S-4. ^1H-^1H COSY spectrum of compound **1** ($CDCl_3$, 600 MHz).

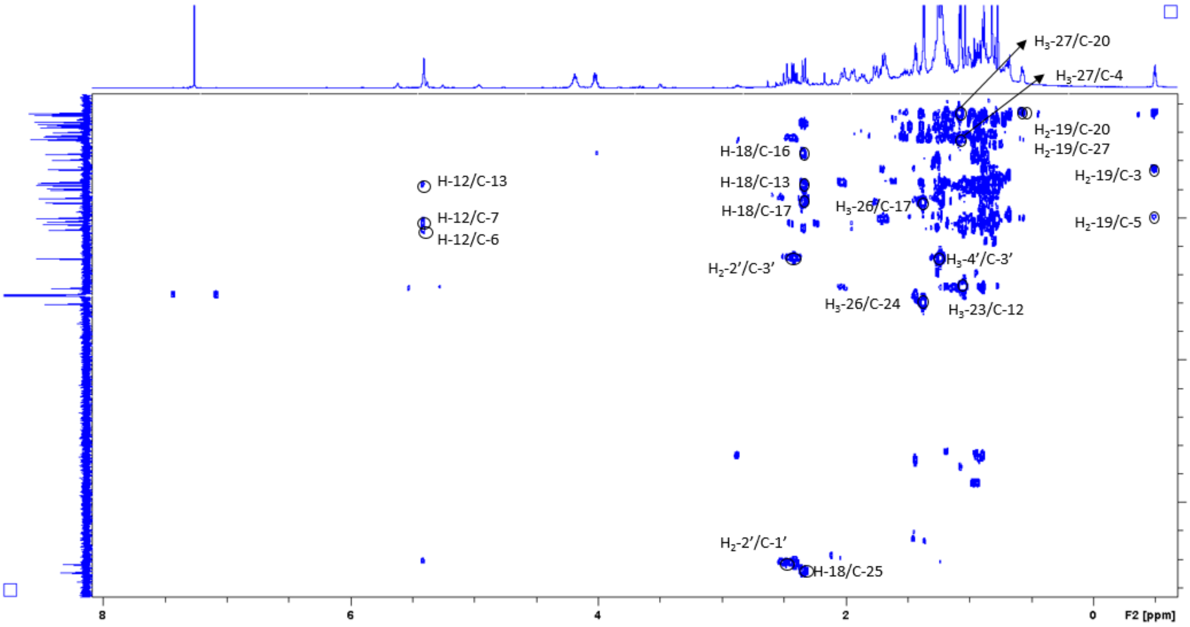

Figure S-5. HMBC spectrum of compound **1** ($CDCl_3$, 600 MHz).

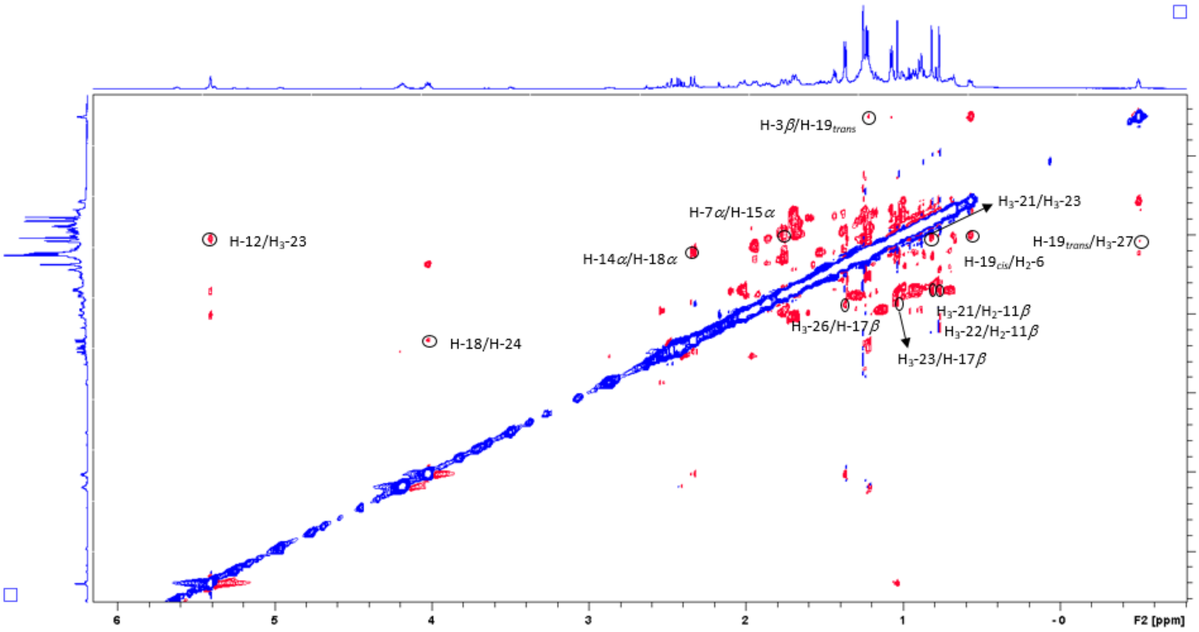

Figure S-6. NOESY spectrum of compound **1** ($CDCl_3$, 600 MHz).

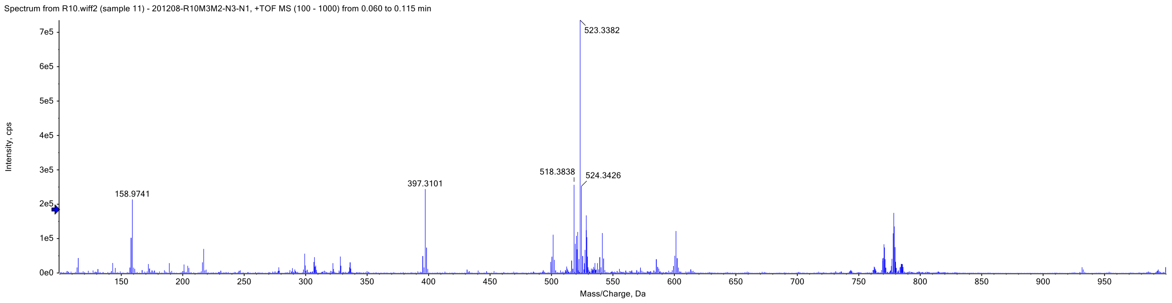

Figure S-7. HRMS spectrum of compound **1**.

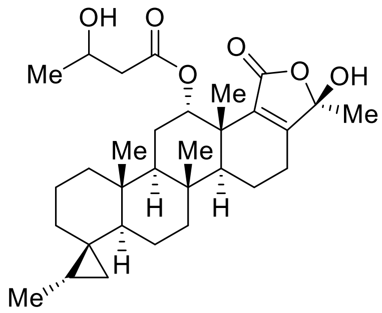

2

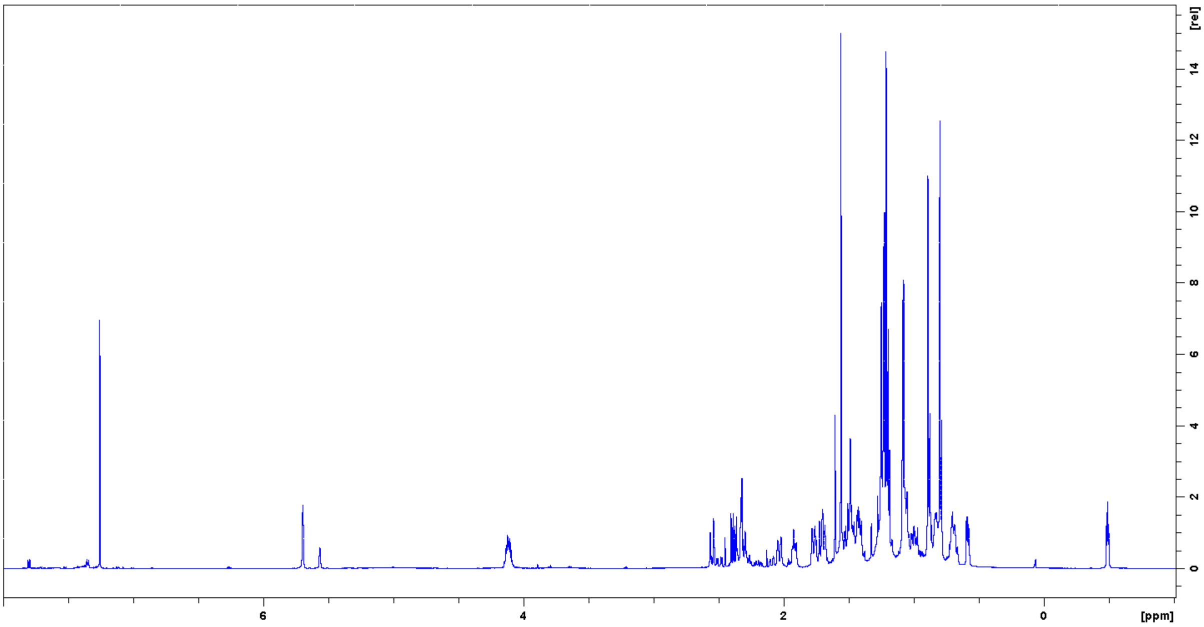

Figure S-8. ^{1}H NMR spectrum of compound **2** ($CDCl_3$, 600 MHz).

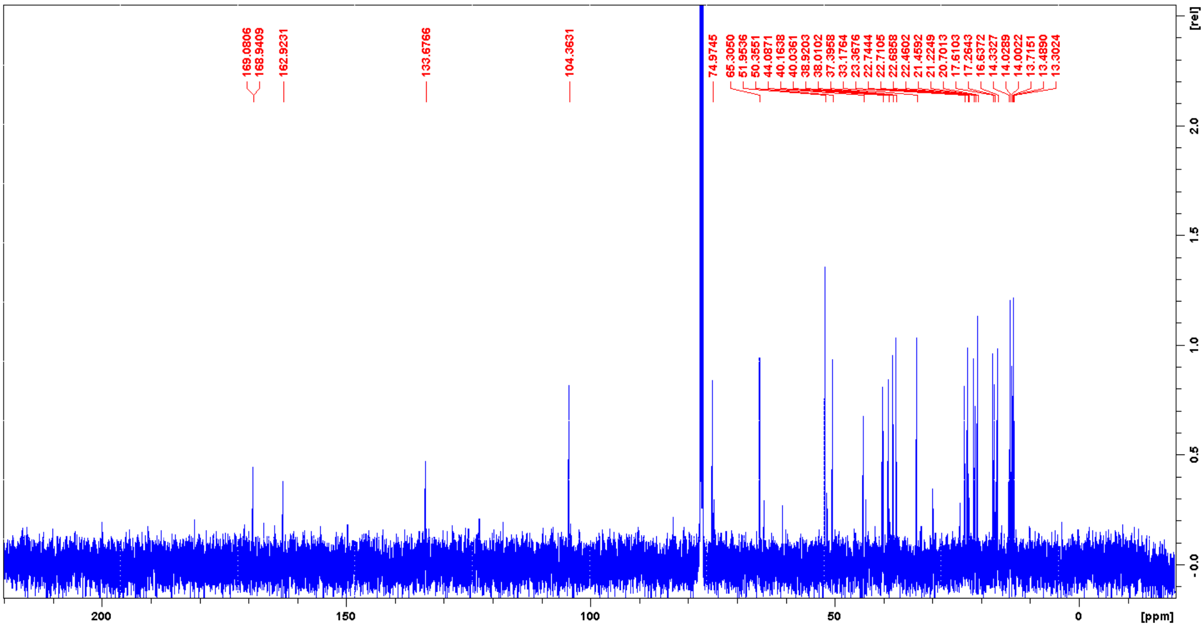

Figure S-9. ^{13}C NMR spectrum of compound **2** ($CDCl_3$, 150 MHz).

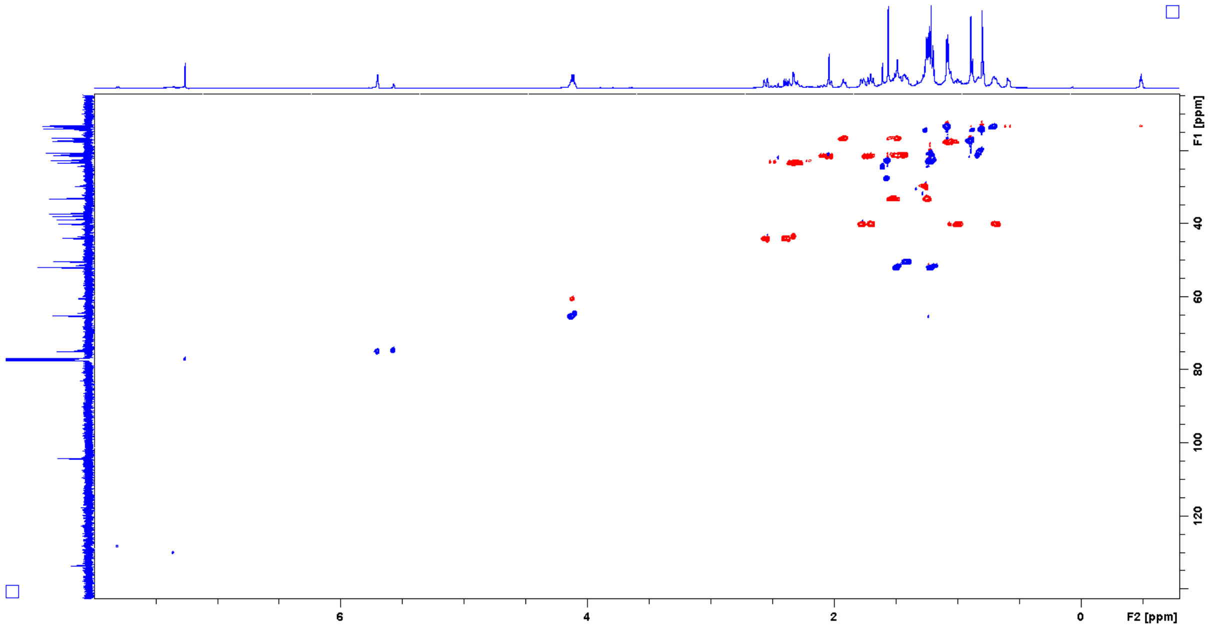

Figure S-10. HSQC spectrum of compound **2** ($CDCl_3$, 600 MHz).

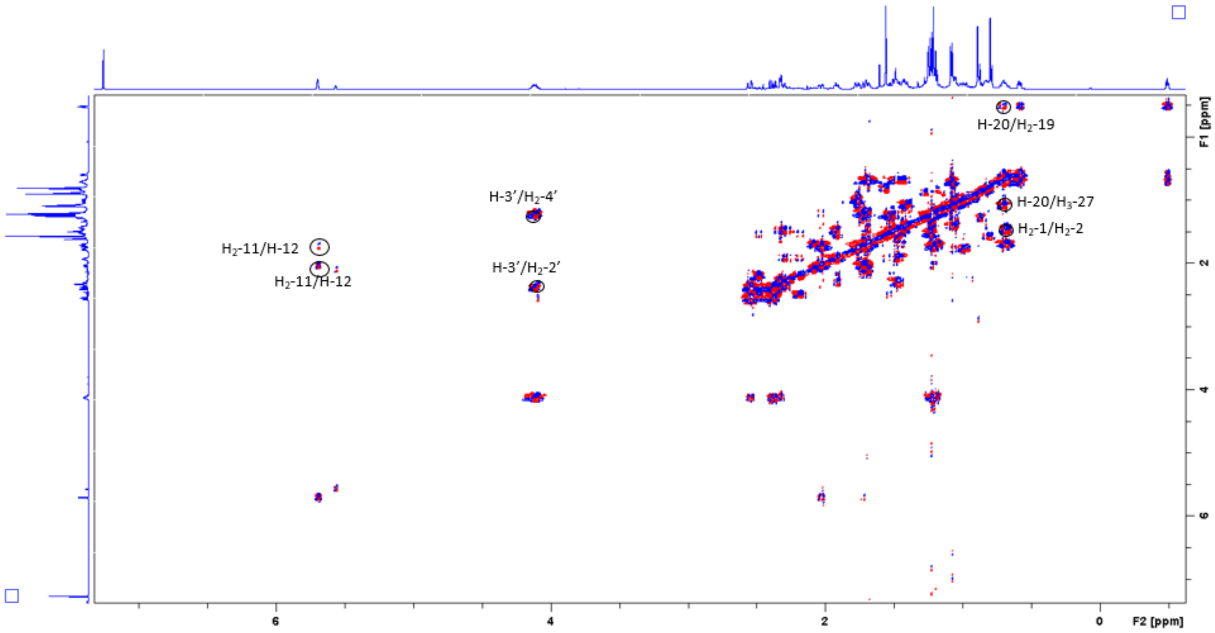

Figure S-11. ^{1}H-^{1}H COSY spectrum of compound **2** ($CDCl_3$, 600 MHz).

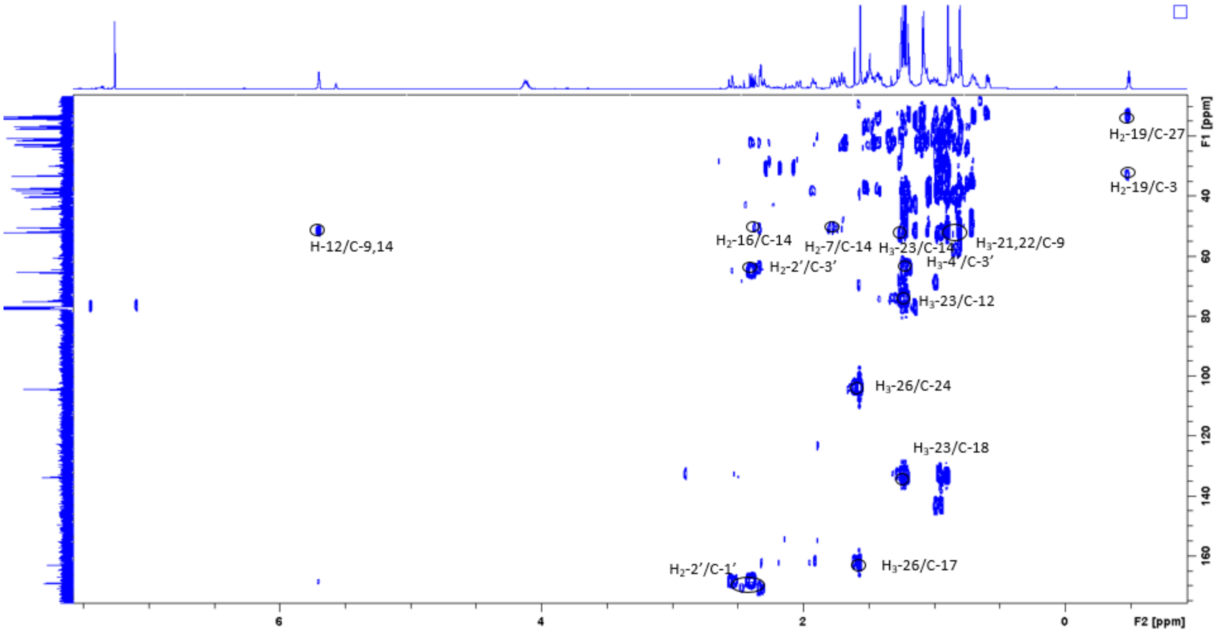

Figure S-12. HMBC spectrum of compound **2** ($CDCl_3$, 600 MHz).

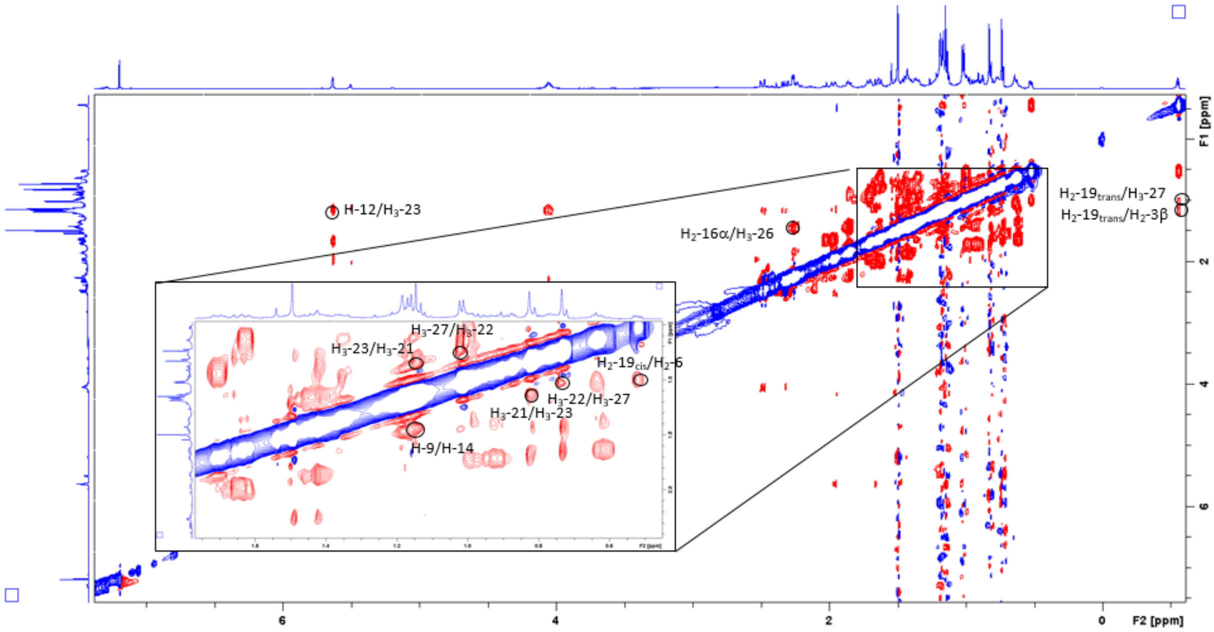

Figure S-13. NOESY spectrum of compound **2** ($CDCl_3$, 600 MHz).

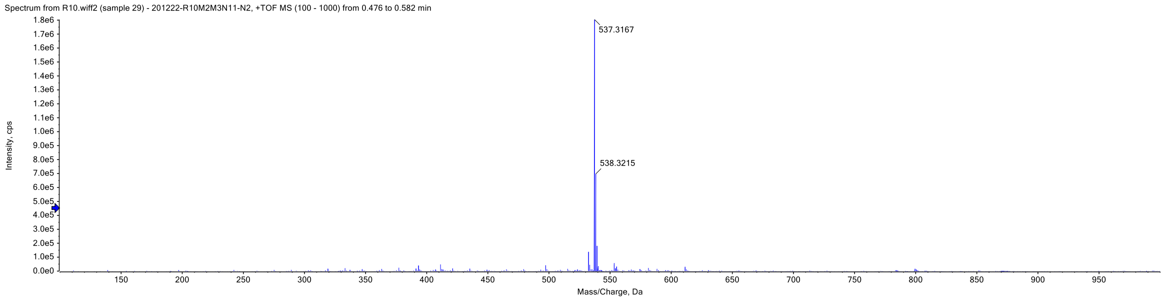

Figure S-14. HRMS spectrum of compound **2**.

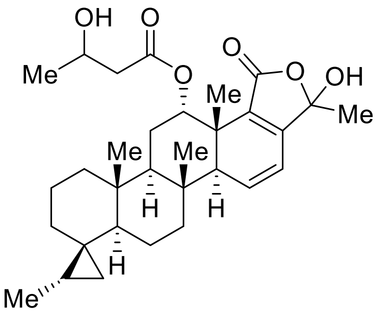

3

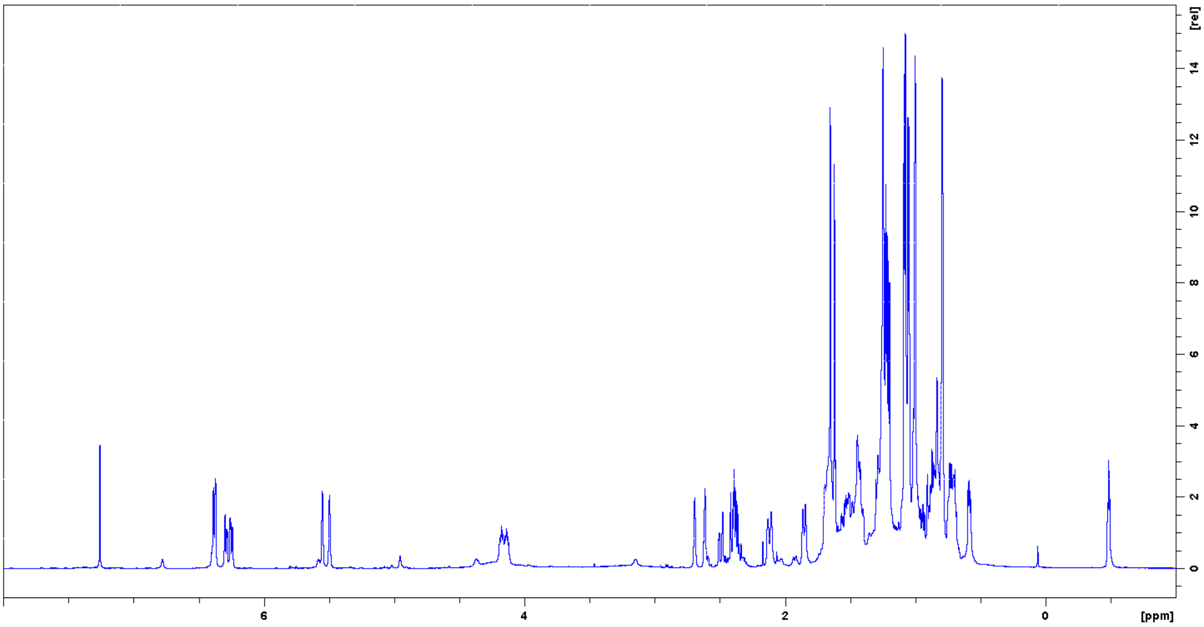

Figure S-15. ^{1}H NMR spectrum of compound **3** ($CDCl_3$, 600 MHz).

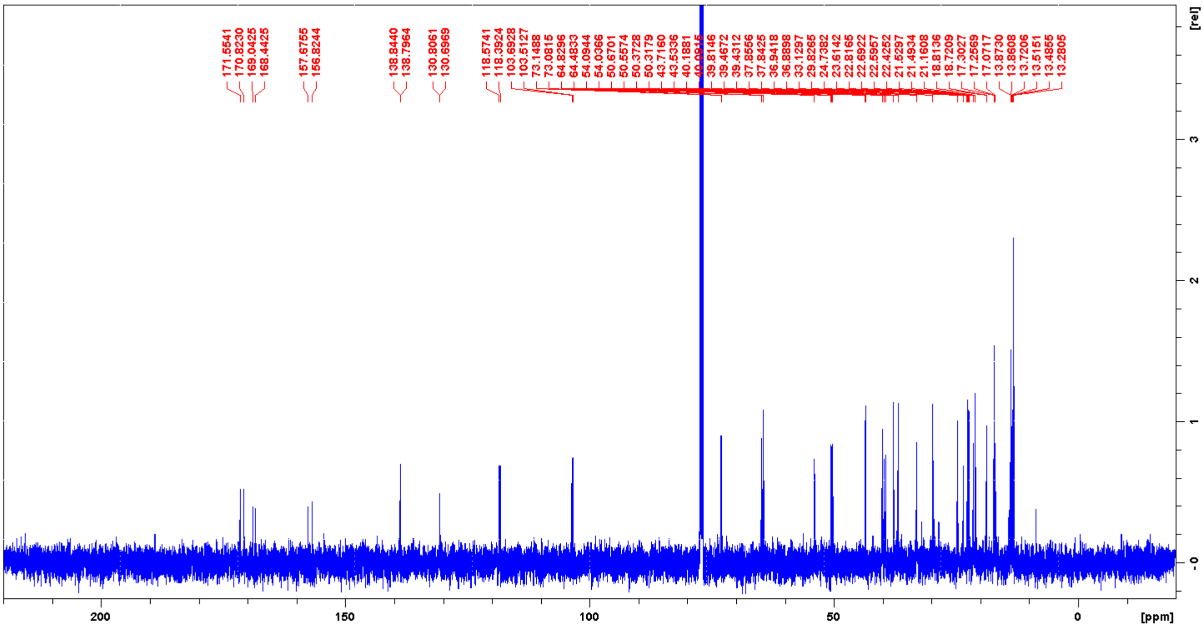

Figure S-16. ^{13}C NMR spectrum of compound **3** ($CDCl_3$, 150 MHz).

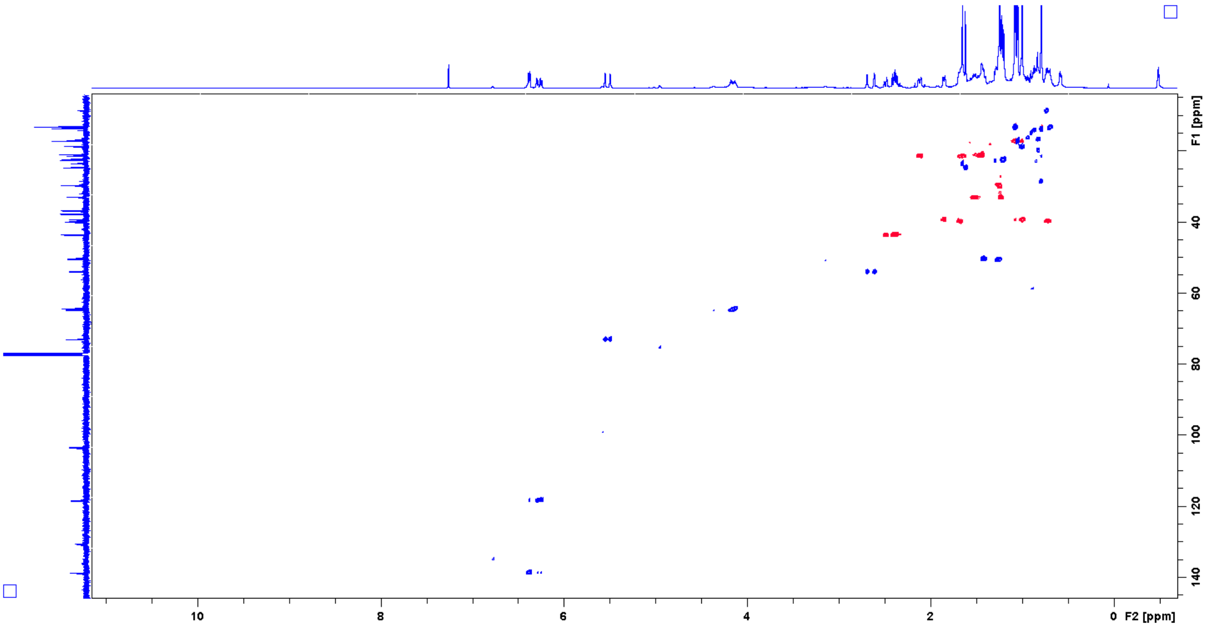

Figure S-17. HSQC spectrum of compound **3** ($CDCl_3$, 600 MHz).

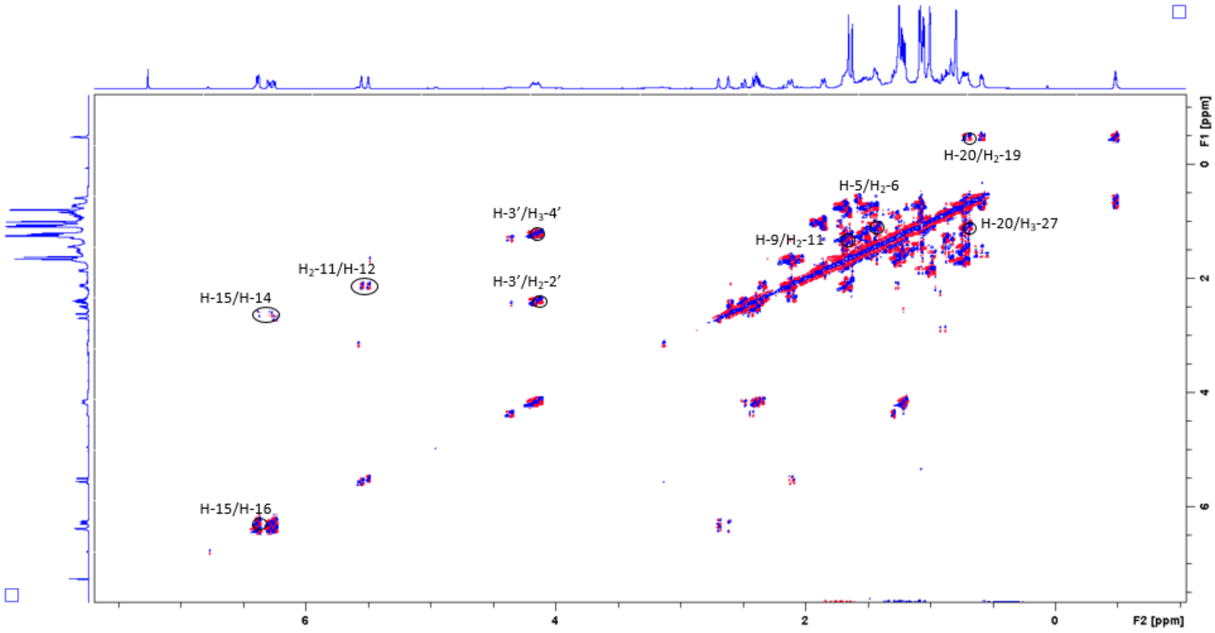

Figure S-18. ^{1}H-^{1}H COSY spectrum of compound **3** ($CDCl_3$, 600 MHz).

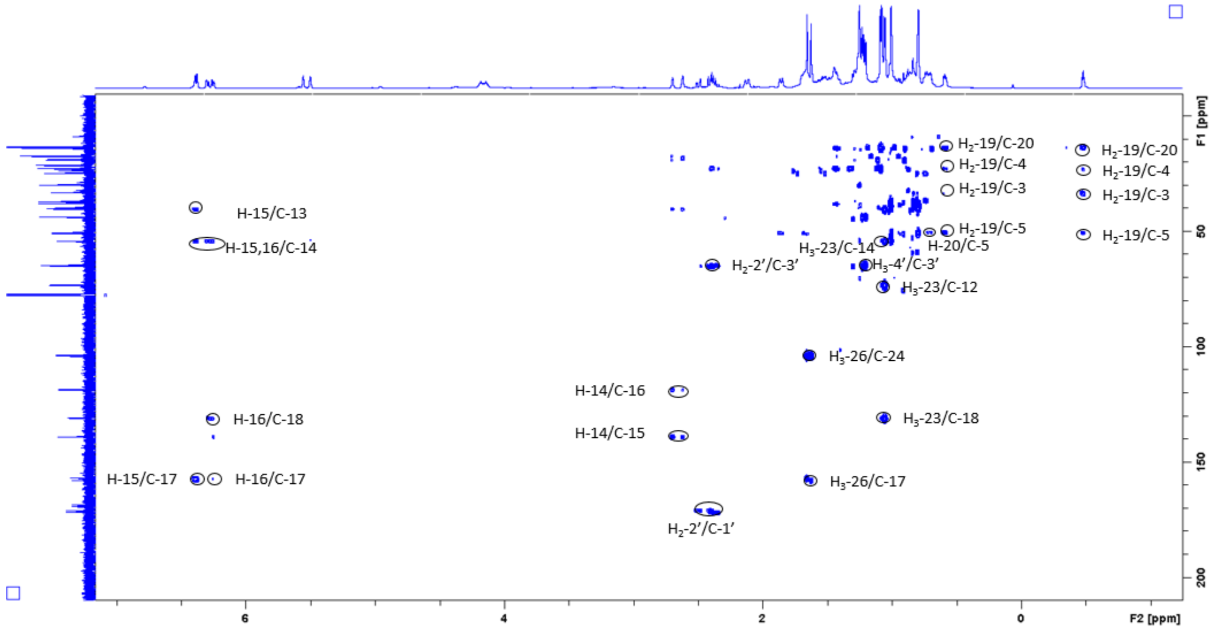

Figure S-19. HMBC spectrum of compound **3** ($CDCl_3$, 600 MHz).

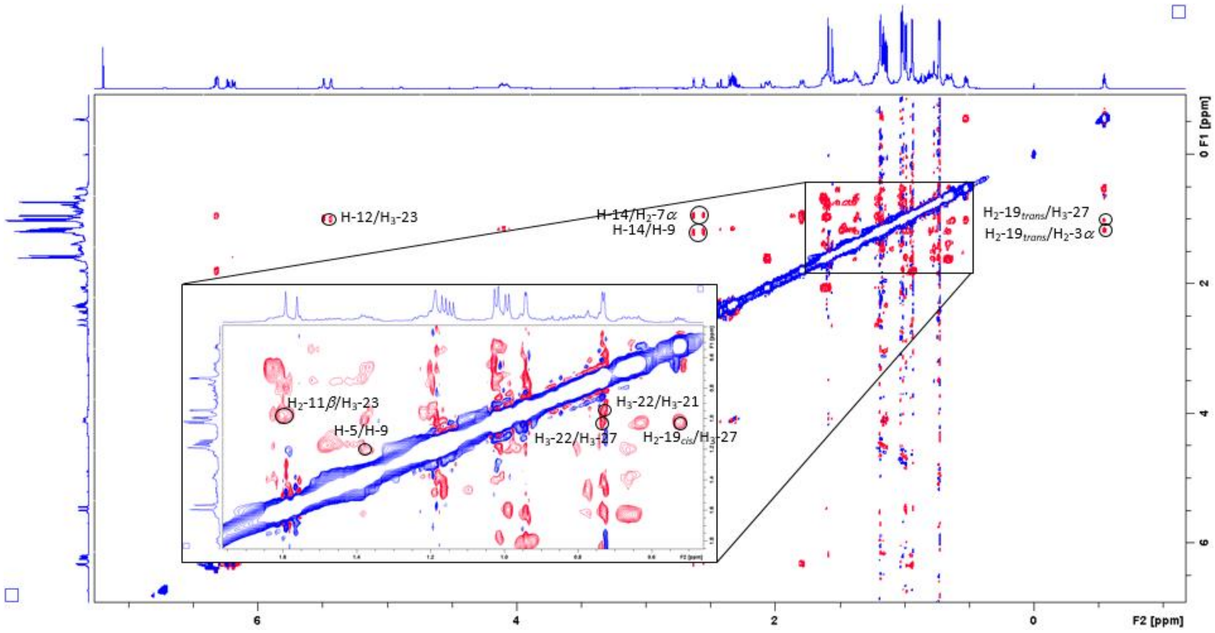

Figure S-20. NOESY spectrum of compound **3** ($CDCl_3$, 600 MHz).

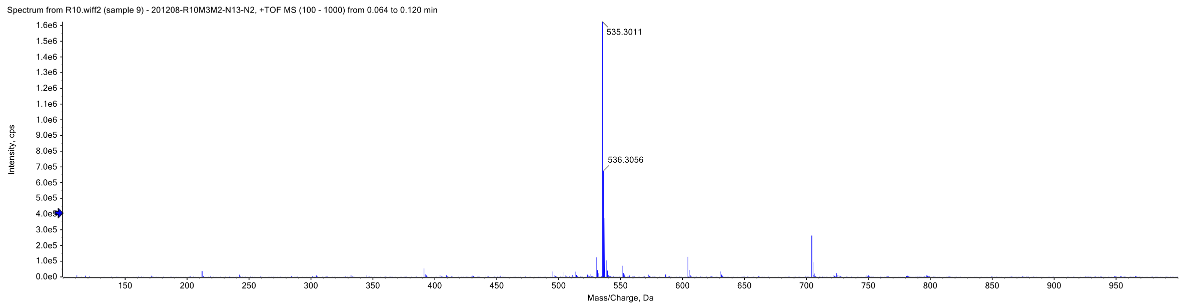

Figure S-21. HRMS spectrum of compound **3**.

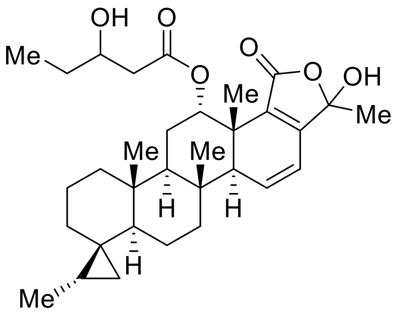

4

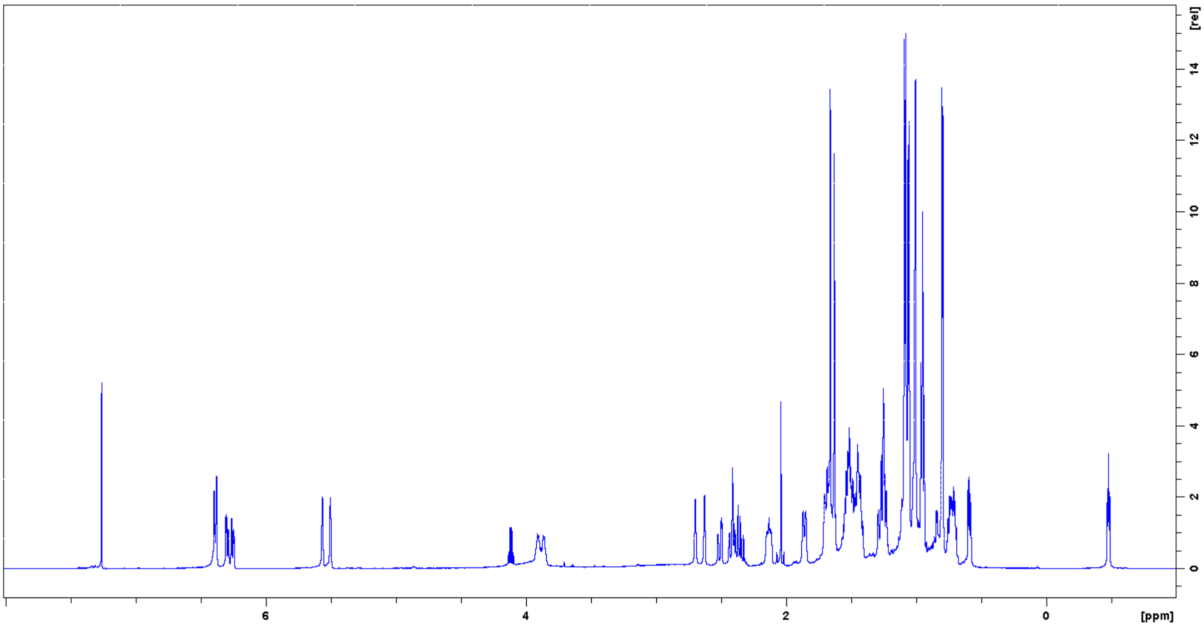

Figure S-22. ^{1}H NMR spectrum of compound **4** ($CDCl_3$, 600 MHz).

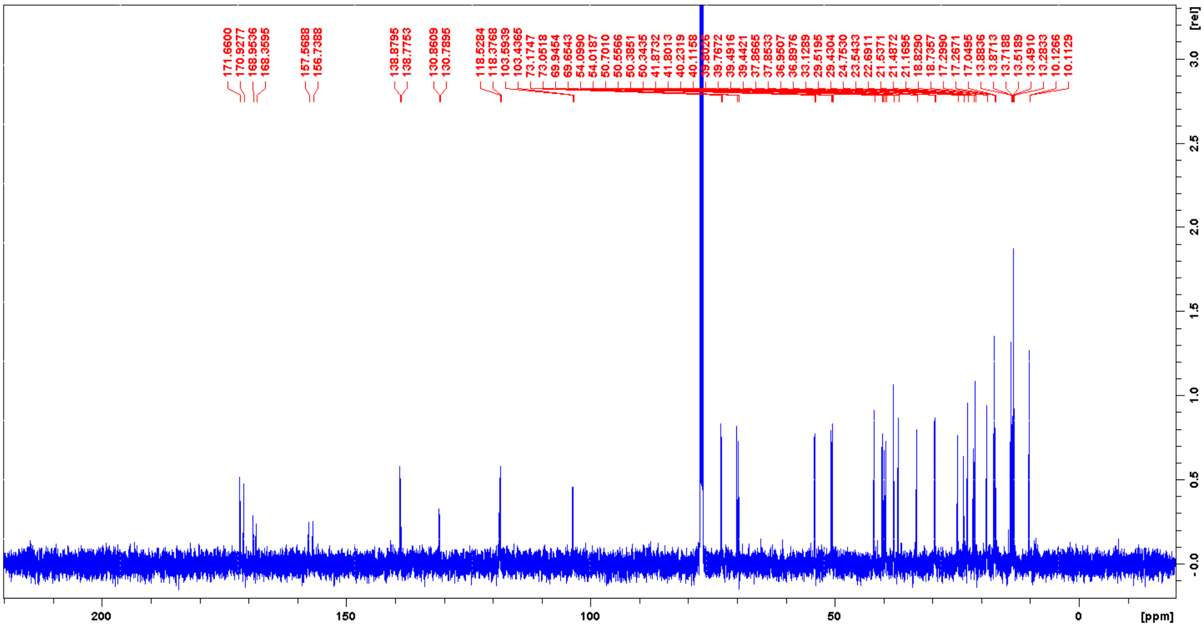

Figure S-23. ^{13}C NMR spectrum of compound **4** ($CDCl_3$, 150 MHz).

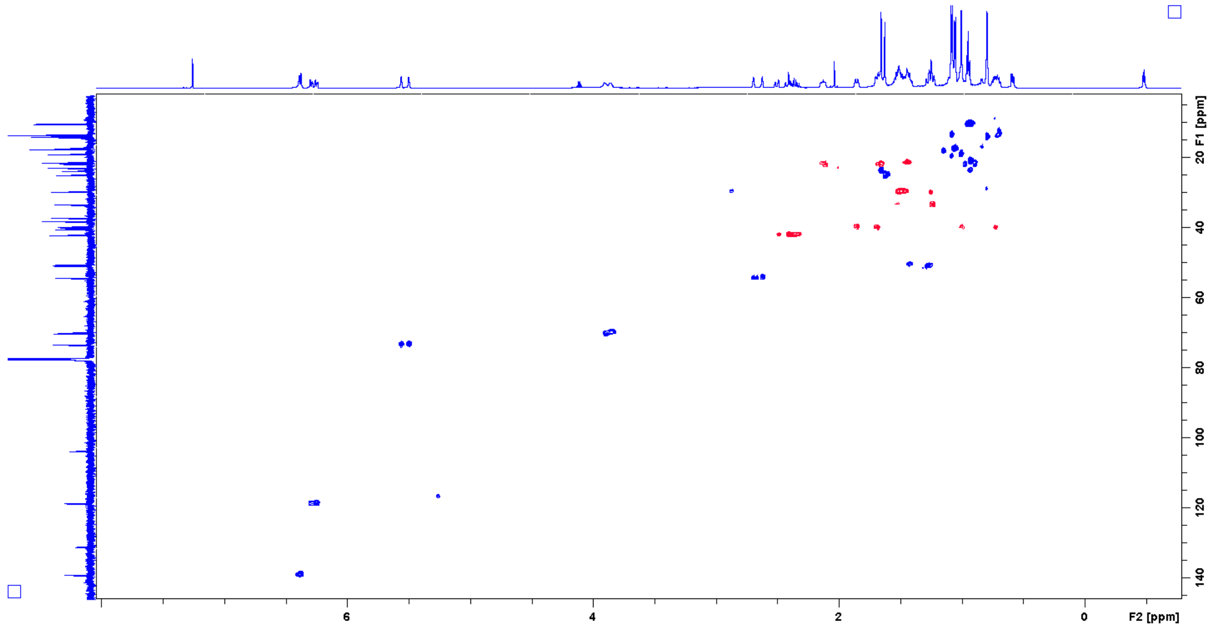

Figure S-24. HSQC spectrum of compound **4** ($CDCl_3$, 600 MHz).

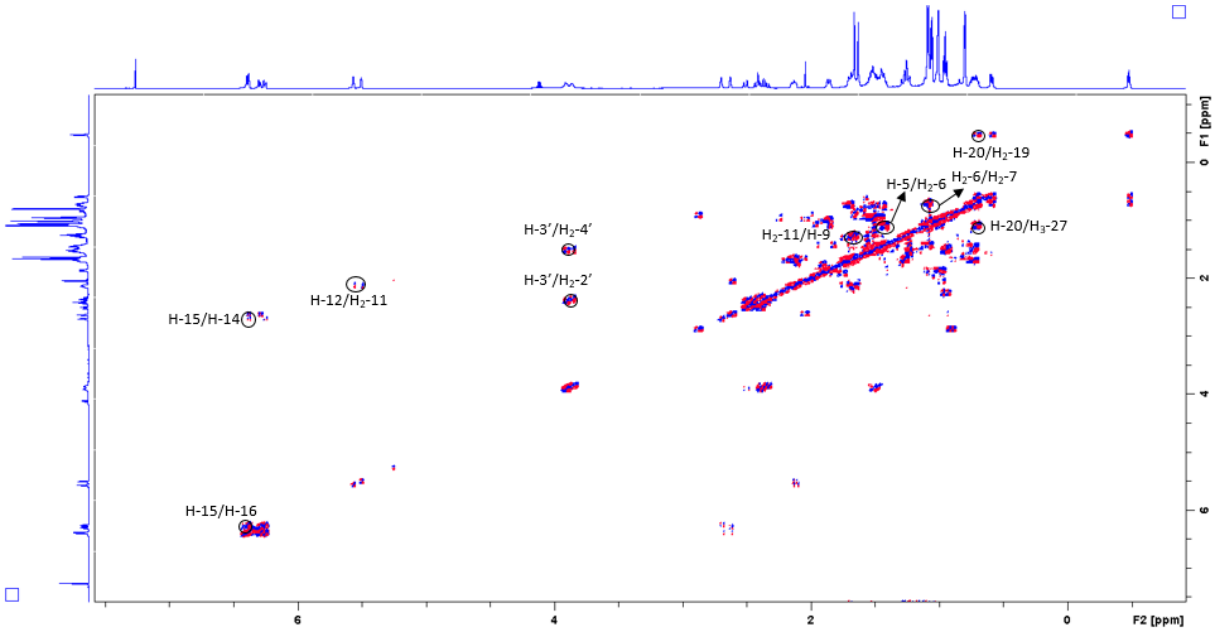

Figure S-25. ^{1}H-^{1}H COSY spectrum of compound **4** ($CDCl_3$, 600 MHz).

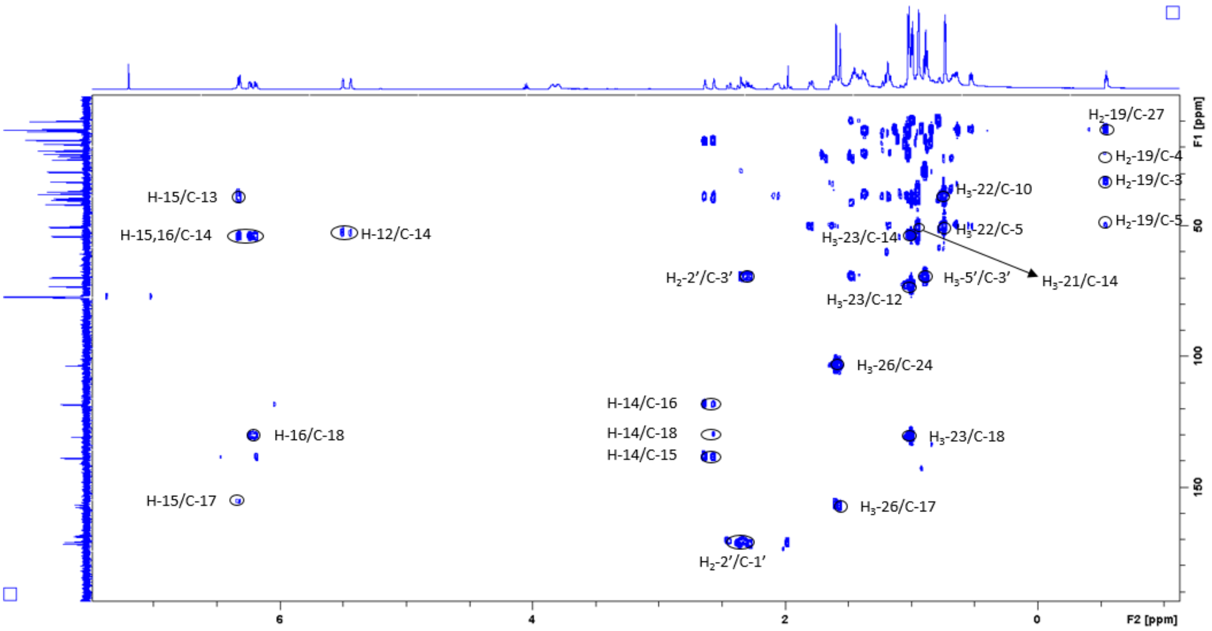

Figure S-26. HMBC spectrum of compound **4** ($CDCl_3$, 600 MHz).

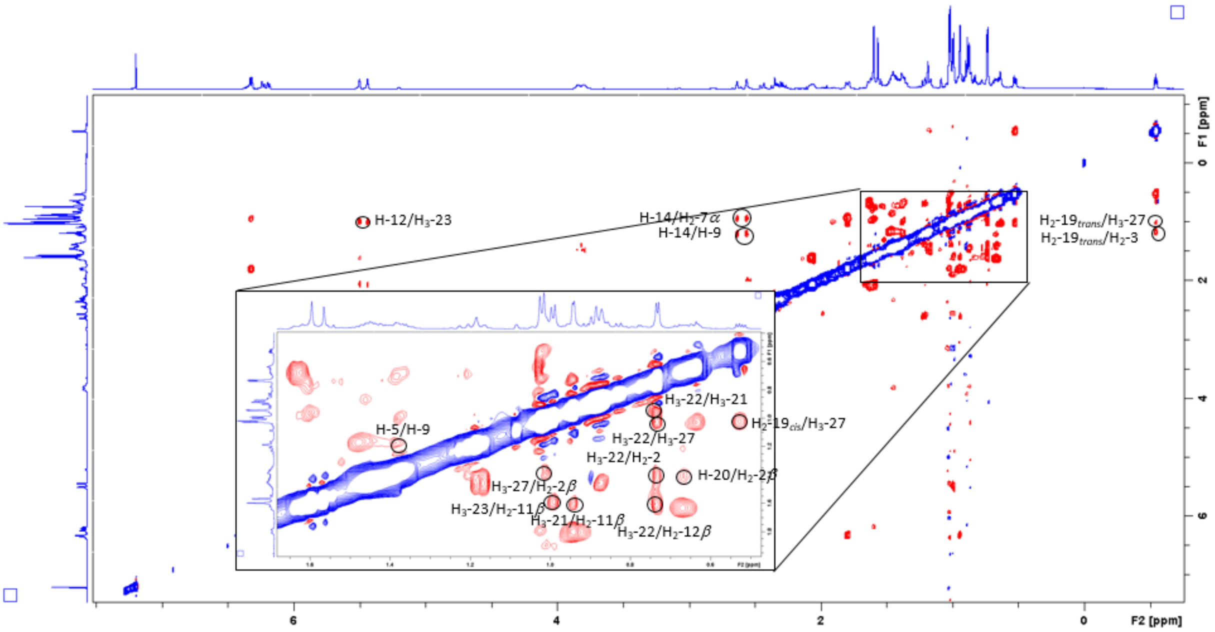

Figure S-27. NOESY spectrum of compound **4** ($CDCl_3$, 600 MHz).

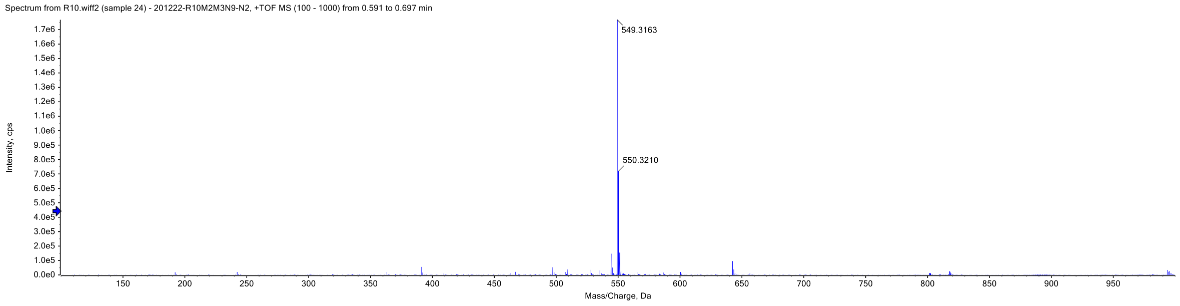

Figure S-28. HRMS spectrum of compound **4**.

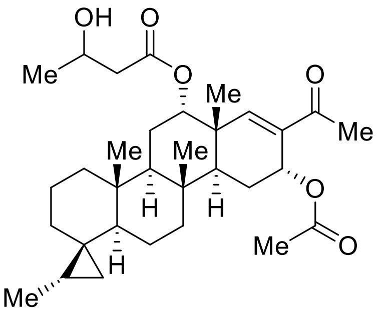

5

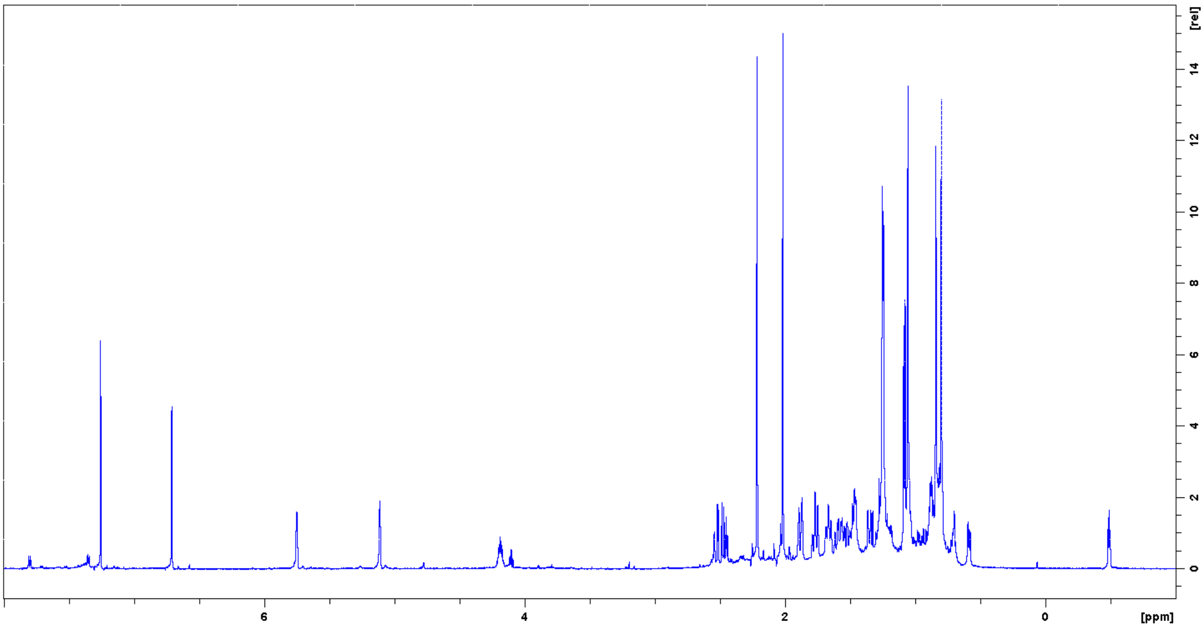

Figure S-29. ¹H NMR spectrum of compound **5** ($CDCl_3$, 600 MHz).

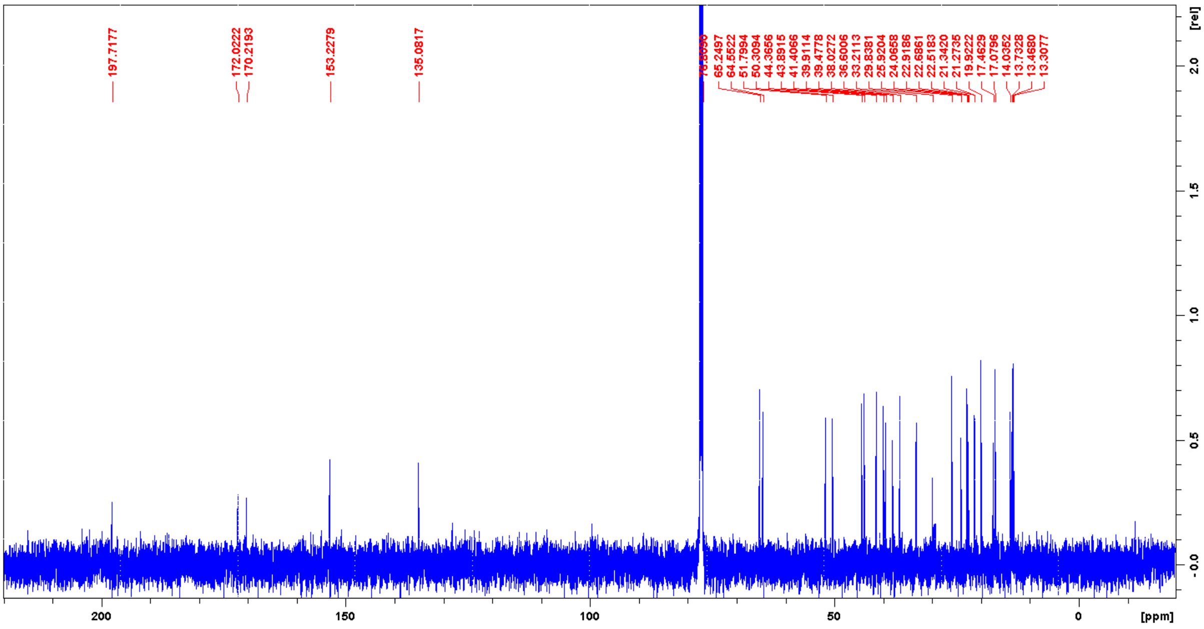

Figure S-30. ¹³C NMR spectrum of compound **5** ($CDCl_3$, 150 MHz).

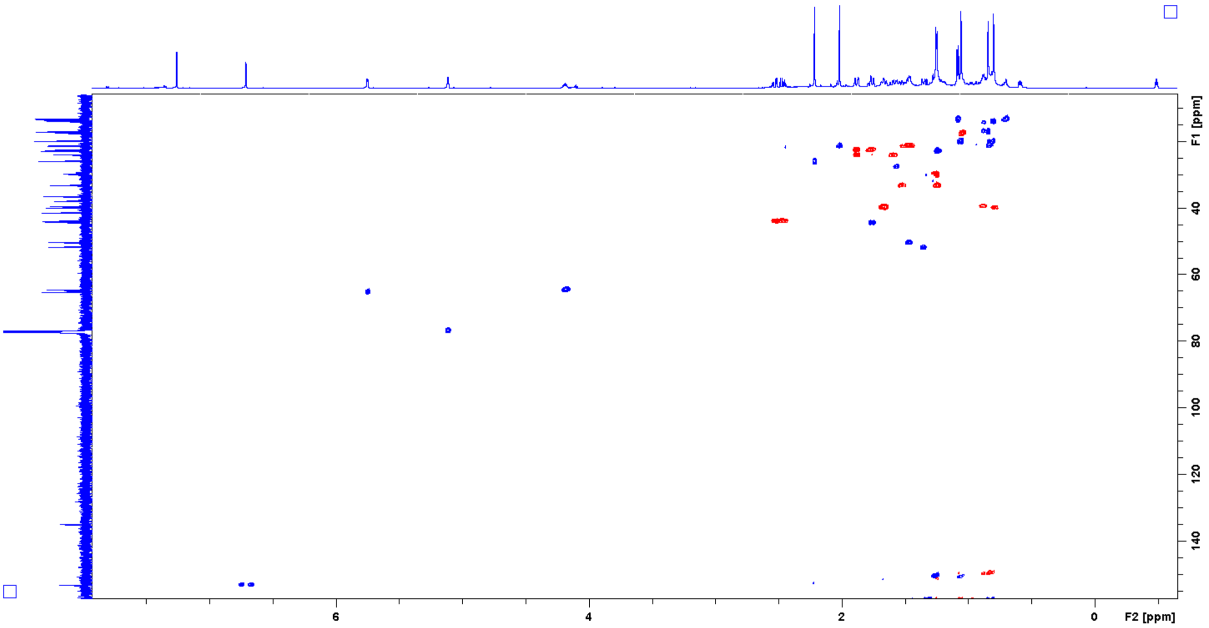

Figure S-31. HSQC spectrum of compound **5** ($CDCl_3$, 600 MHz).

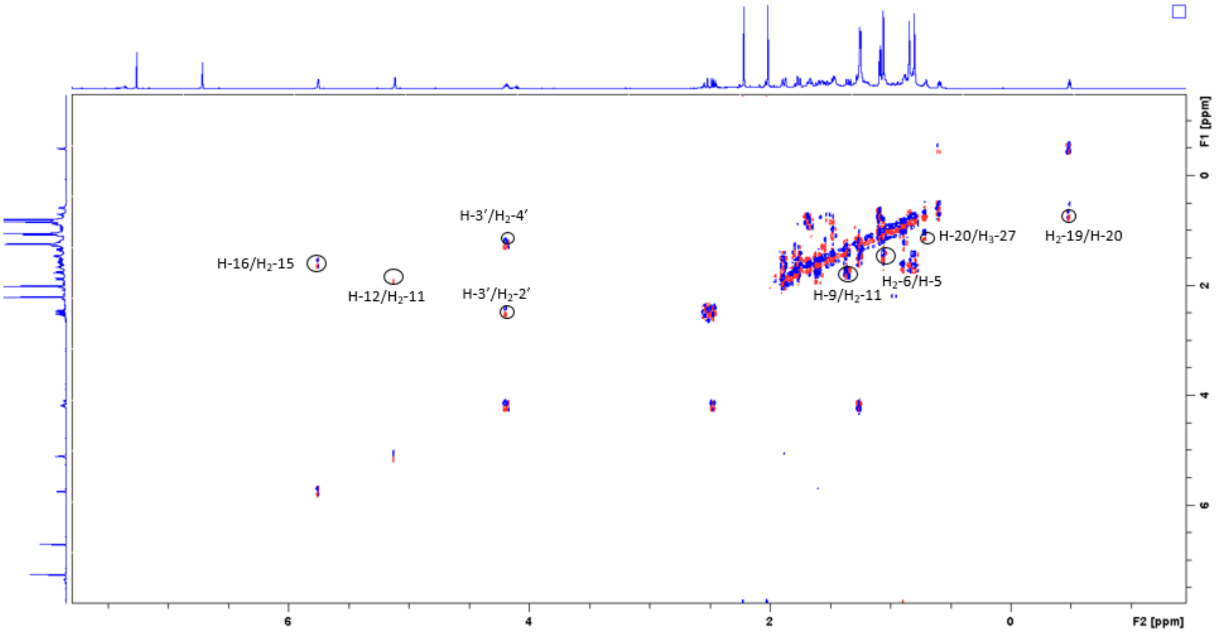

Figure S-32. ^{1}H-^{1}H COSY spectrum of compound **5** ($CDCl_3$, 600 MHz).

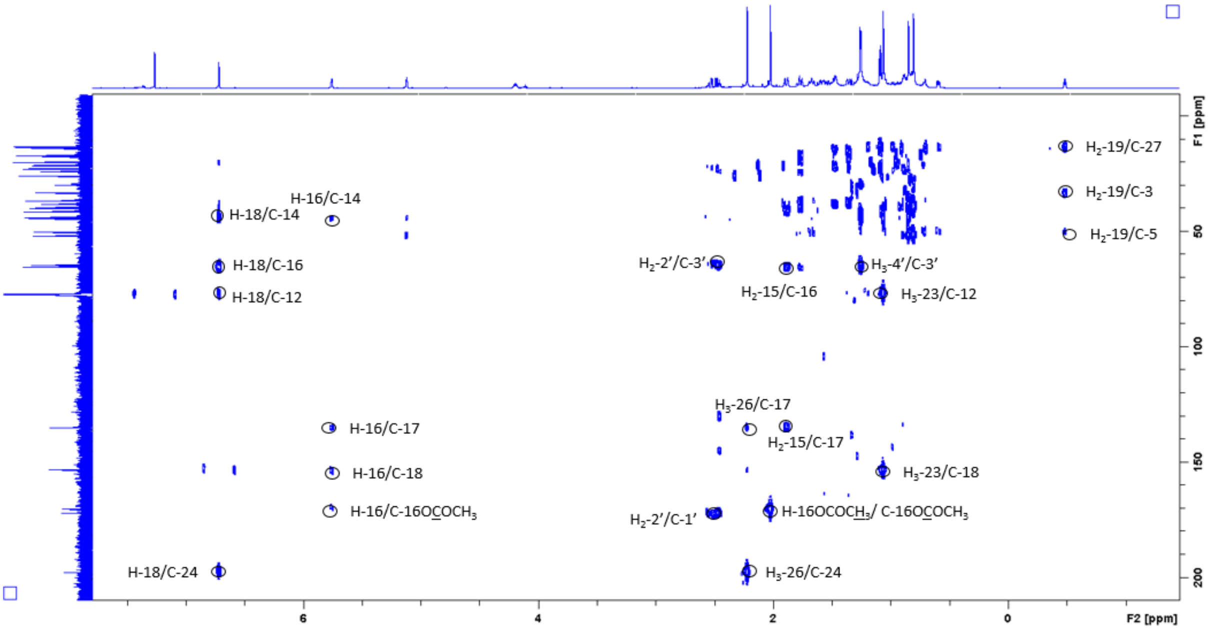

Figure S-33. HMBC spectrum of compound **5** ($CDCl_3$, 600 MHz).

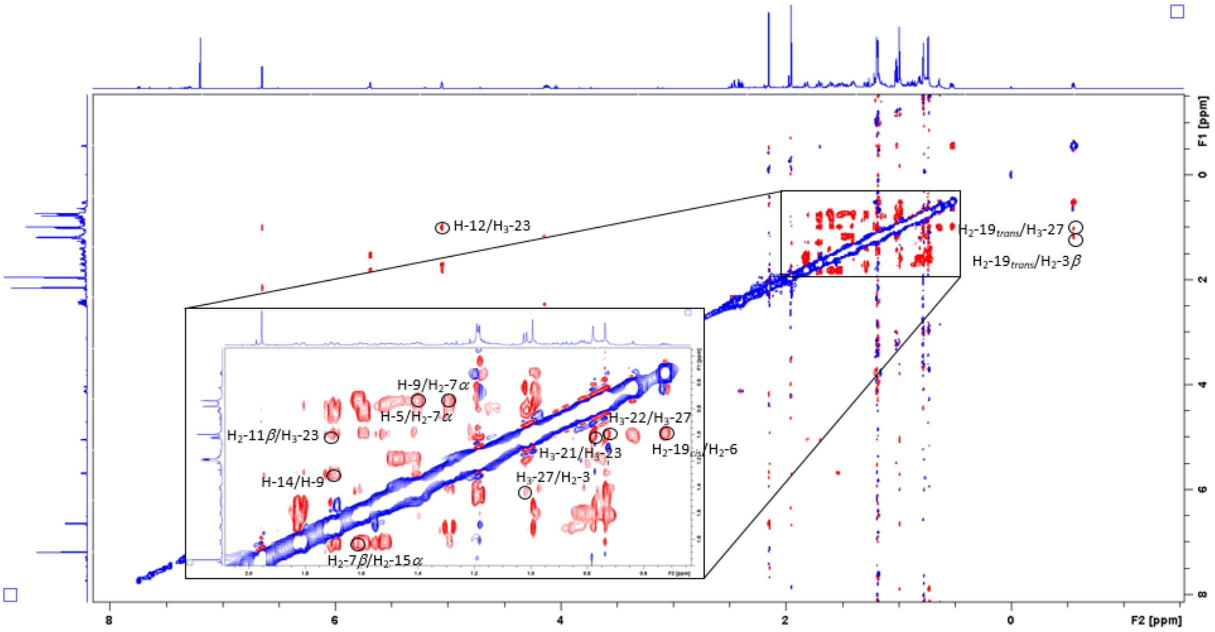

Figure S-34. NOESY spectrum of compound **5** ($CDCl_3$, 600 MHz).

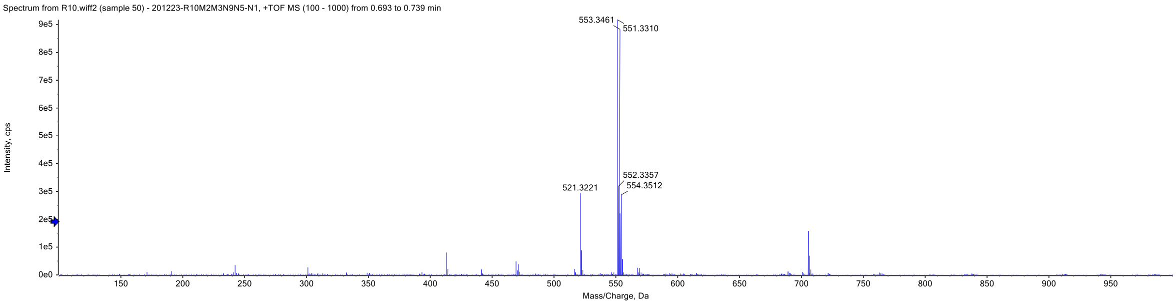

Figure S-35. HRMS spectrum of compound **5**.

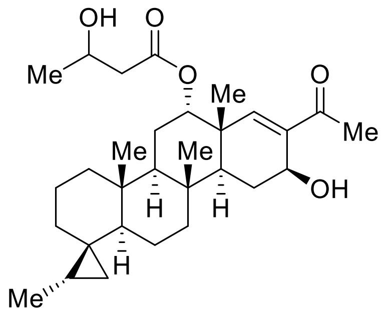

6

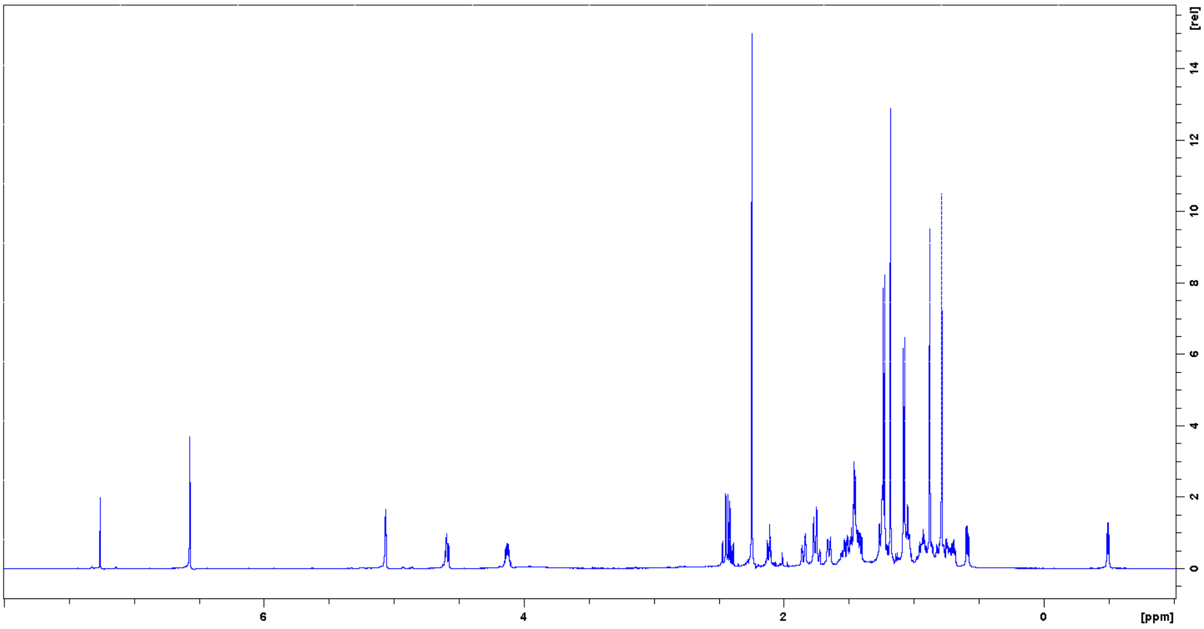

Figure S-36. ^{1}H NMR spectrum of compound **6** ($CDCl_3$, 600 MHz).

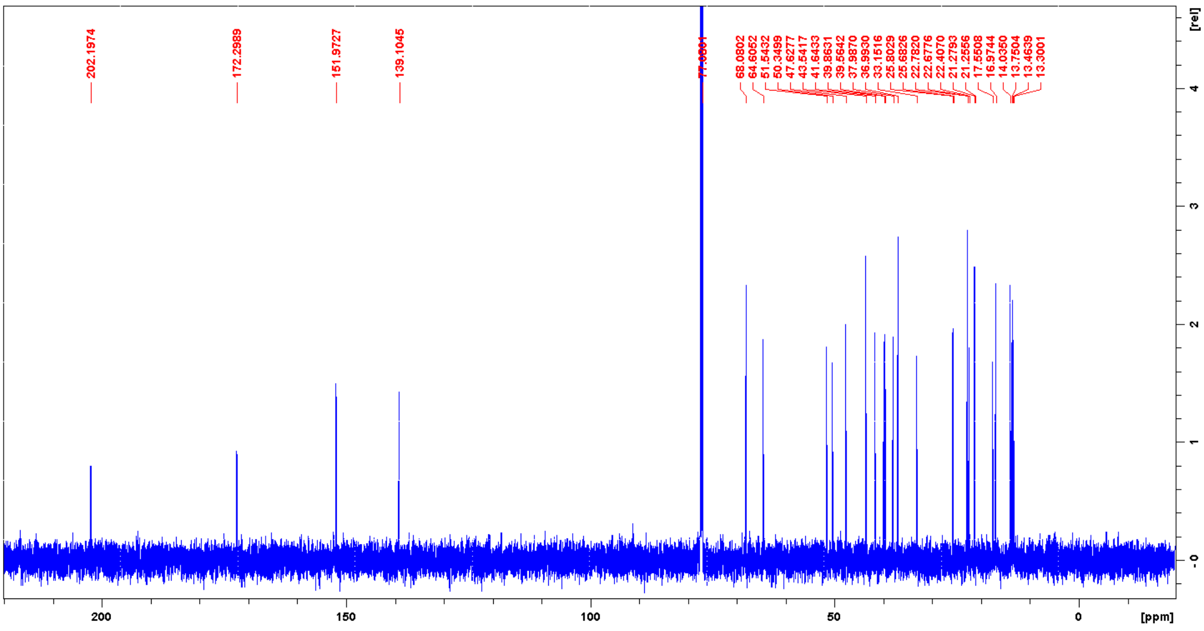

Figure S-37. ^{13}C NMR spectrum of compound **6** ($CDCl_3$, 150 MHz).

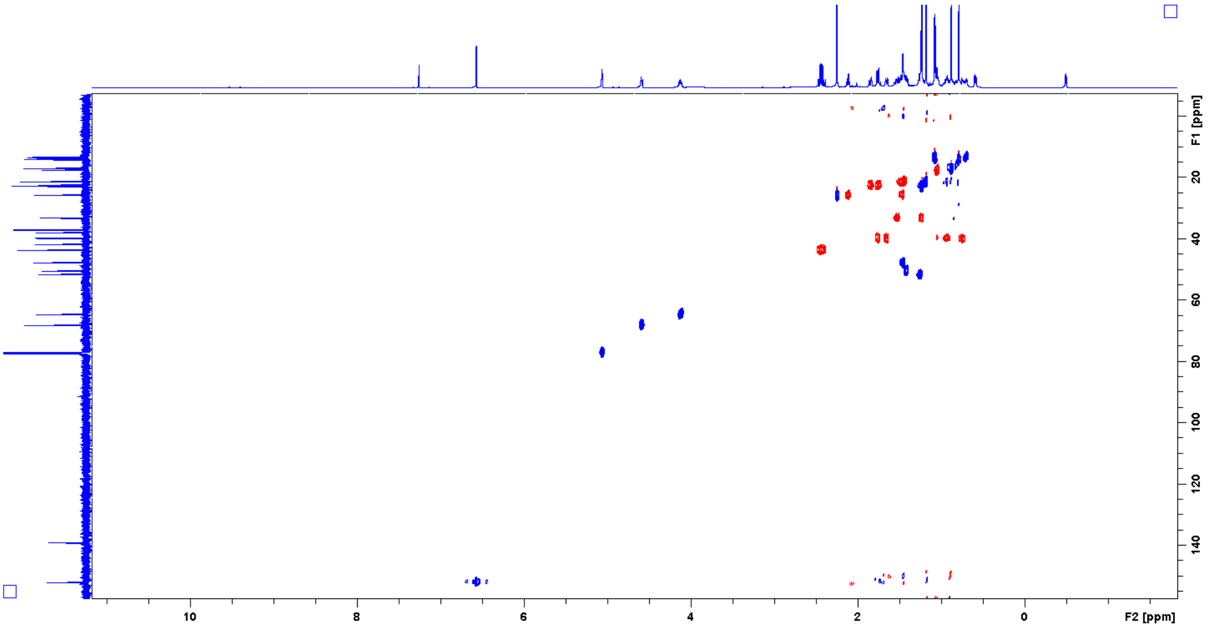

Figure S-38. HSQC spectrum of compound **6** ($CDCl_3$, 600 MHz).

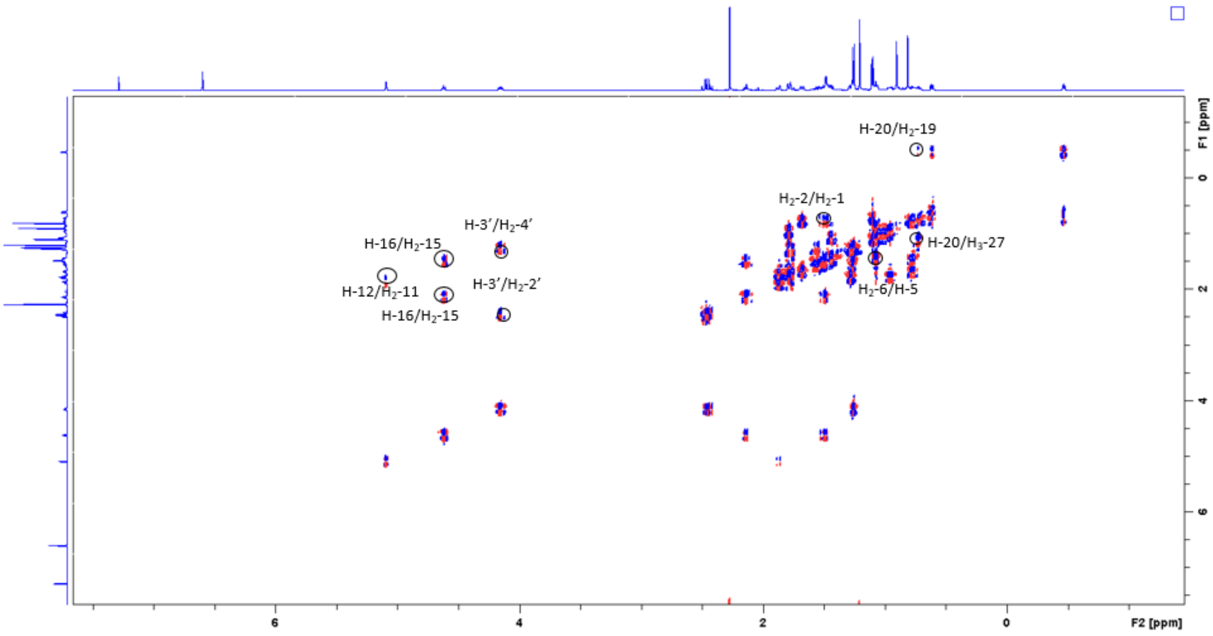

Figure S-39. ^{1}H-^{1}H COSY spectrum of compound **6** ($CDCl_3$, 600 MHz).

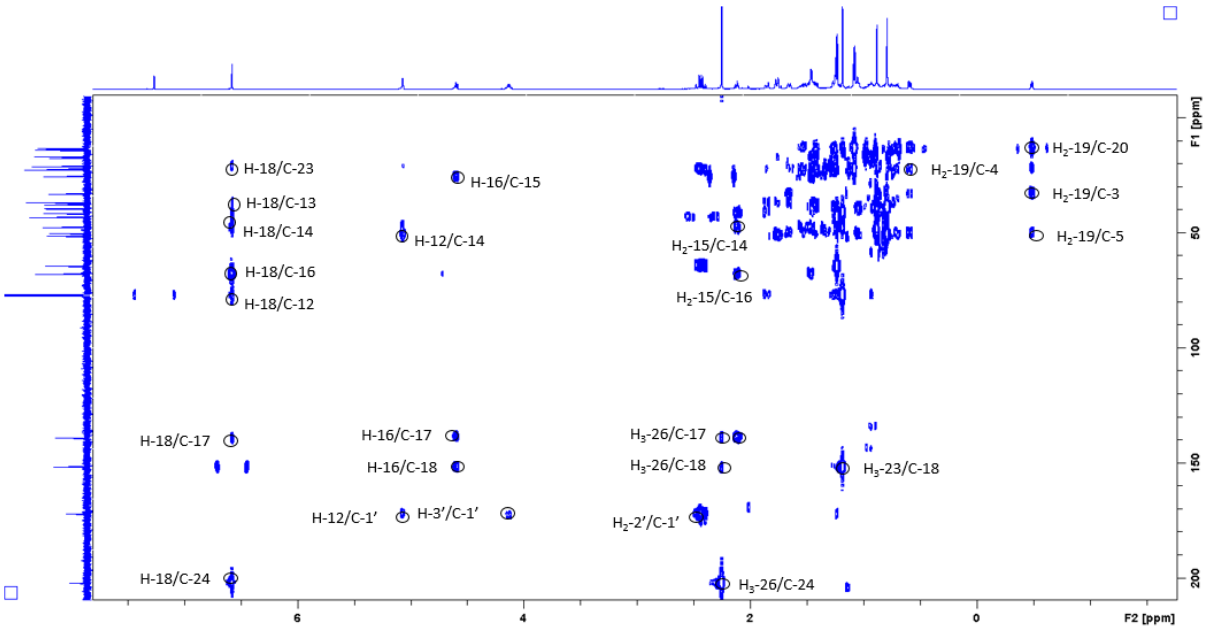

Figure S-40. HMBC spectrum of compound **6** ($CDCl_3$, 600 MHz).

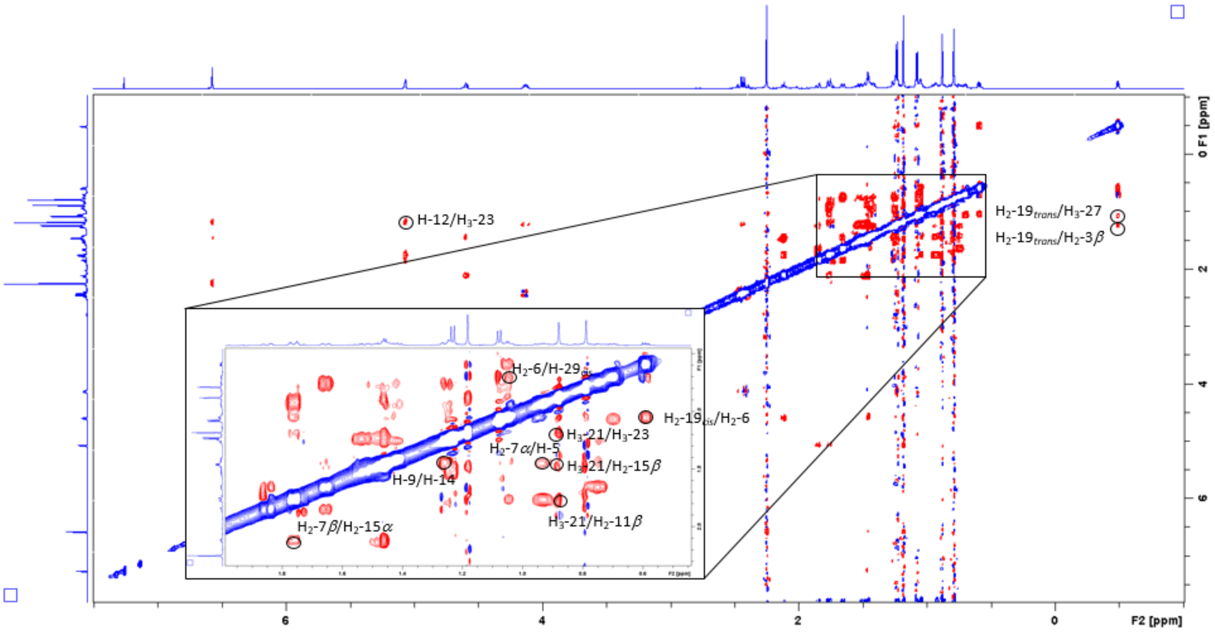

Figure S-41. NOESY spectrum of compound **6** ($CDCl_3$, 600 MHz).

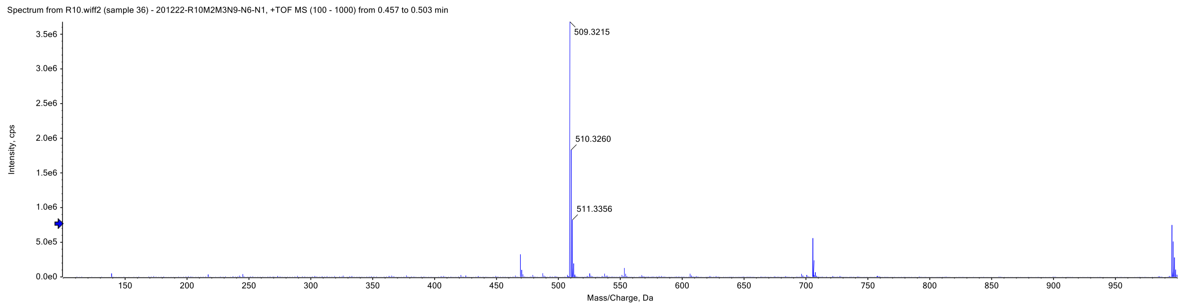

Figure S-42. HRMS spectrum of compound **6**.

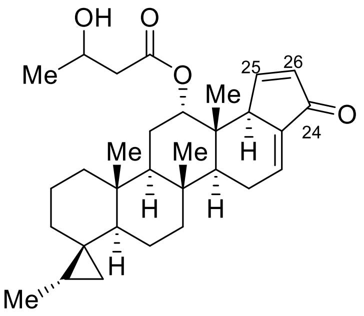

7

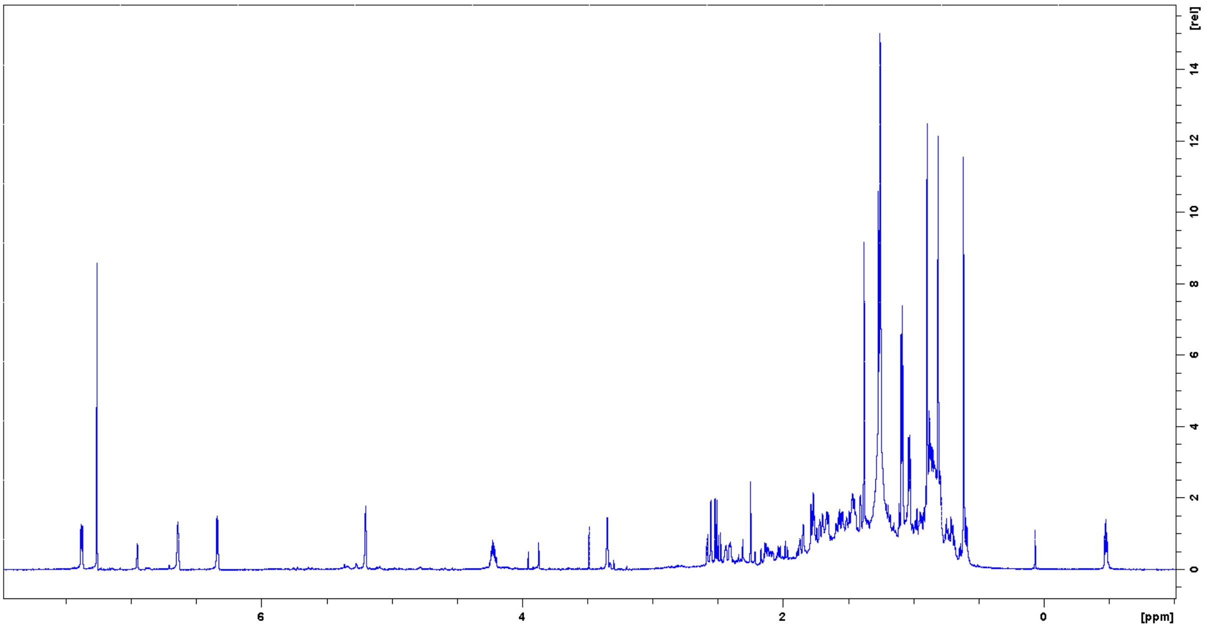

Figure S-43. ^{1}H NMR spectrum of compound **7** ($CDCl_3$, 600 MHz).

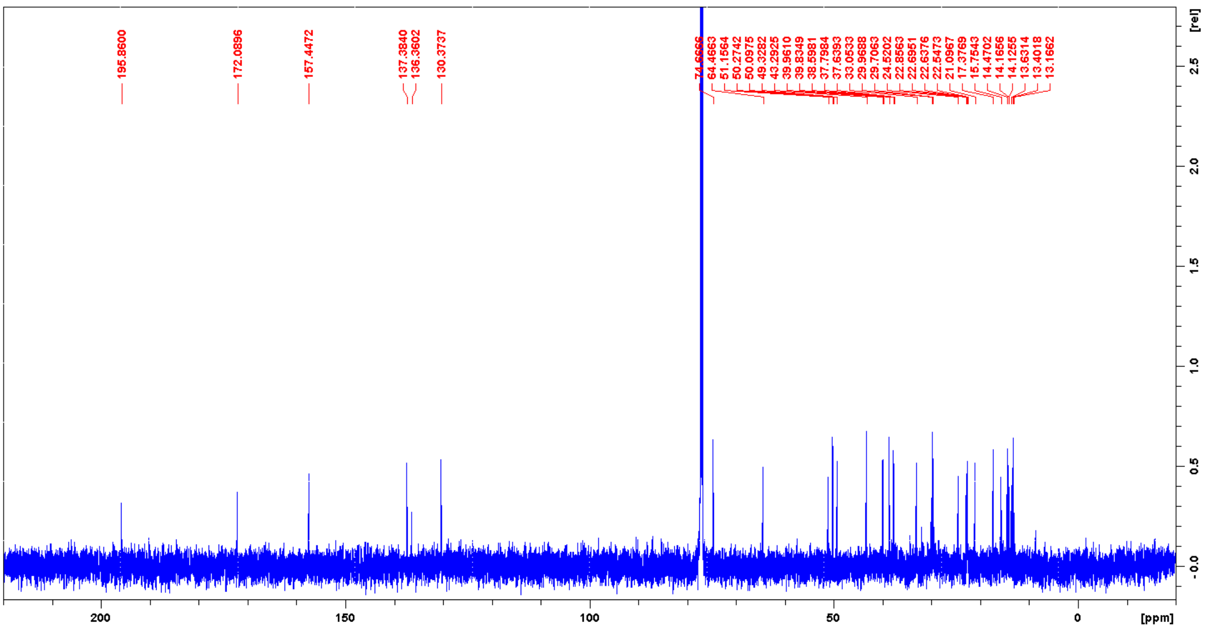

Figure S-44. ^{13}C NMR spectrum of compound **7** ($CDCl_3$, 150 MHz).

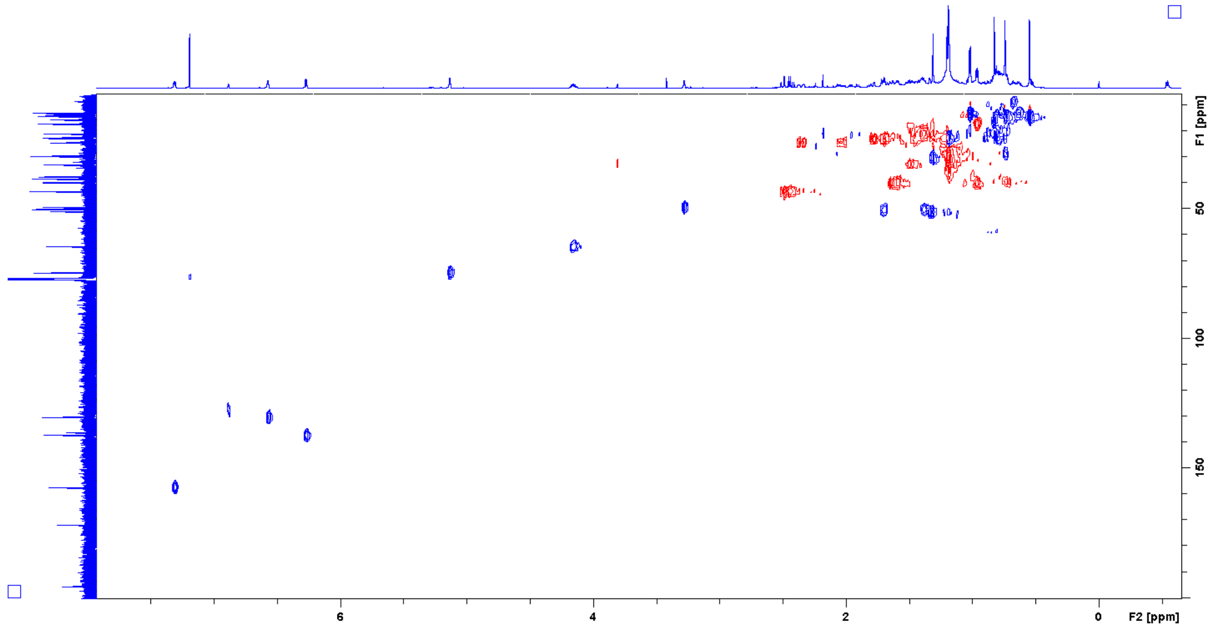

Figure S-45. HSQC spectrum of compound **7** ($CDCl_3$, 600 MHz).

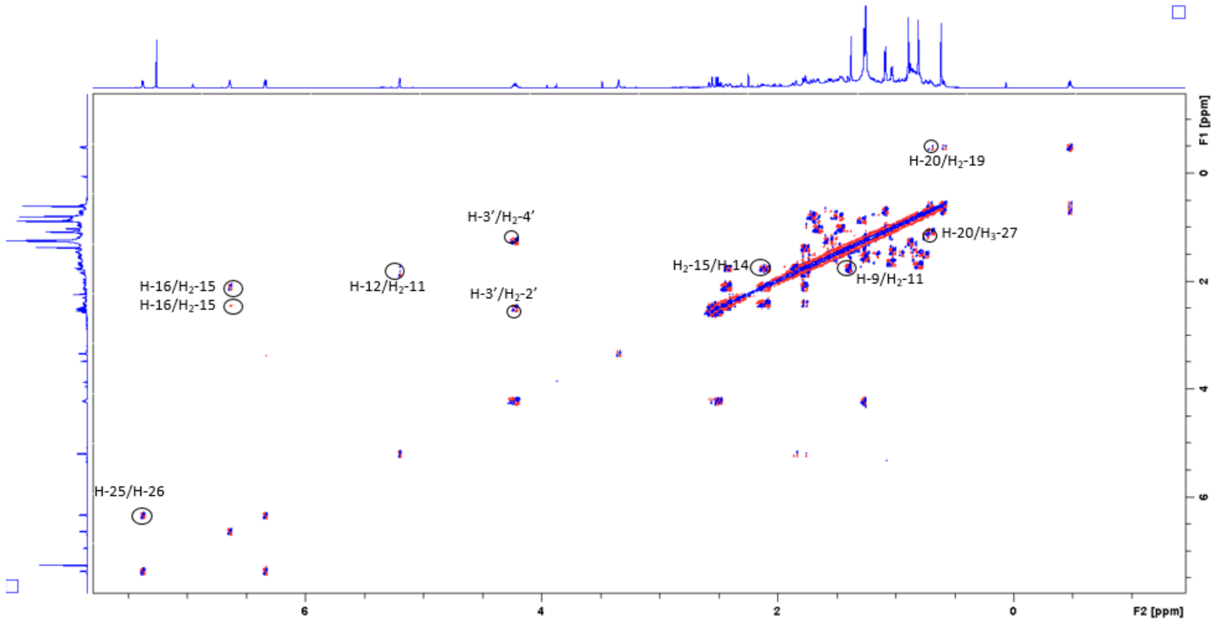

Figure S-46. ^1H-^1H COSY spectrum of compound **7** ($CDCl_3$, 600 MHz).

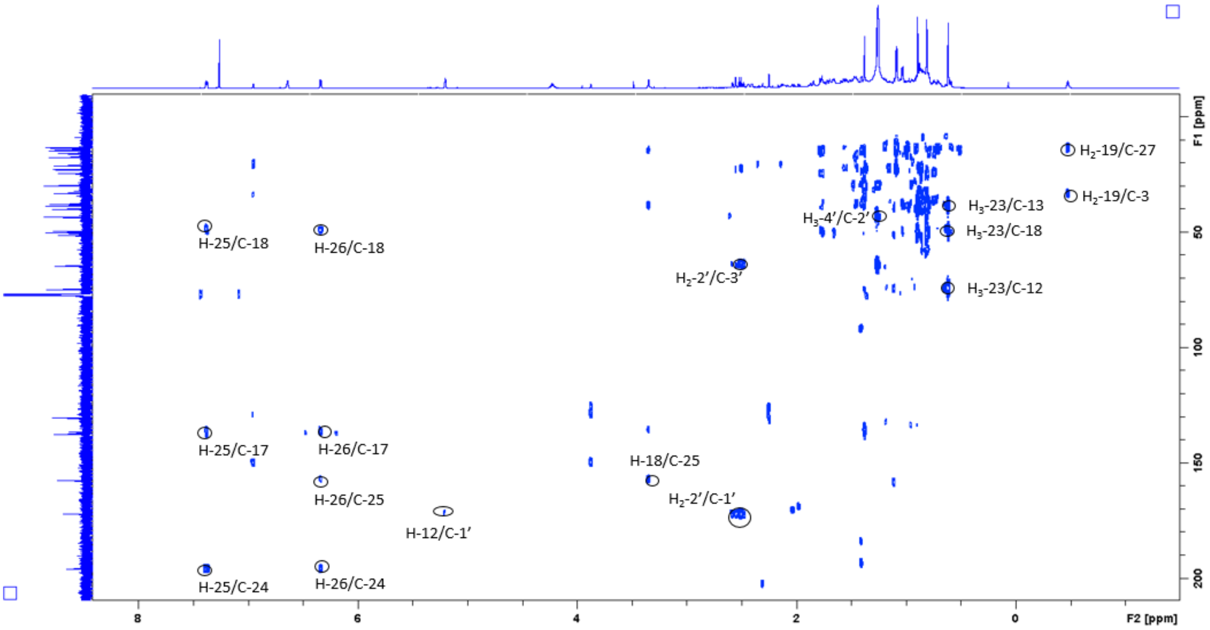

Figure S-47. HMBC spectrum of compound **7** ($CDCl_3$, 600 MHz).

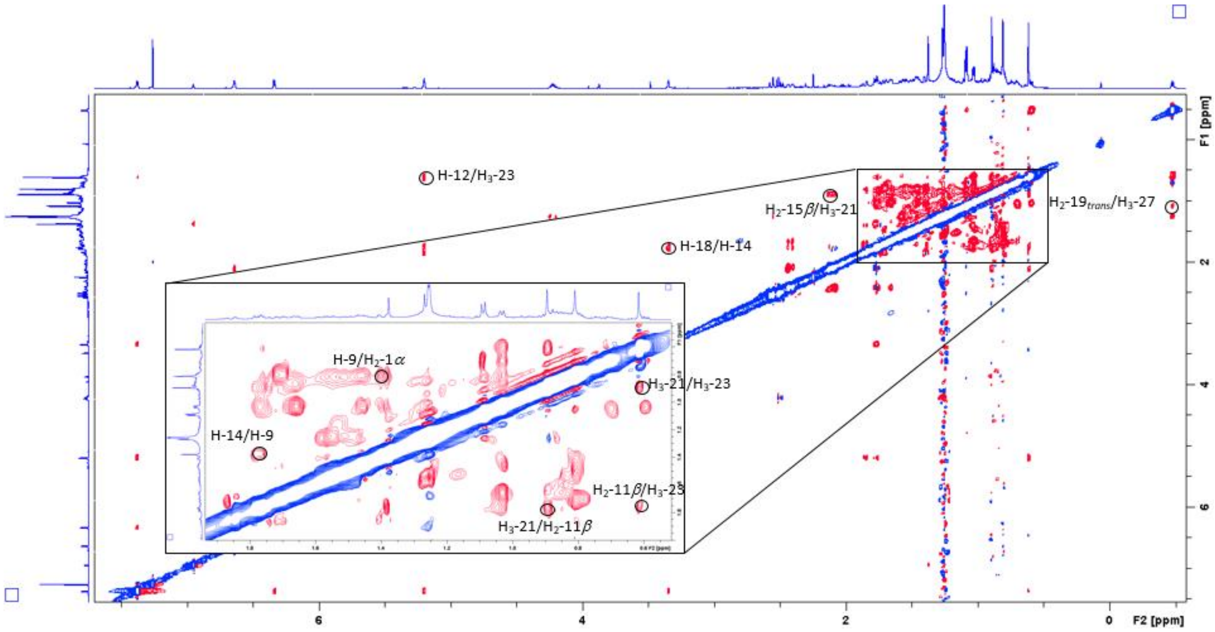

Figure S-48. NOESY spectrum of compound **7** ($CDCl_3$, 600 MHz).

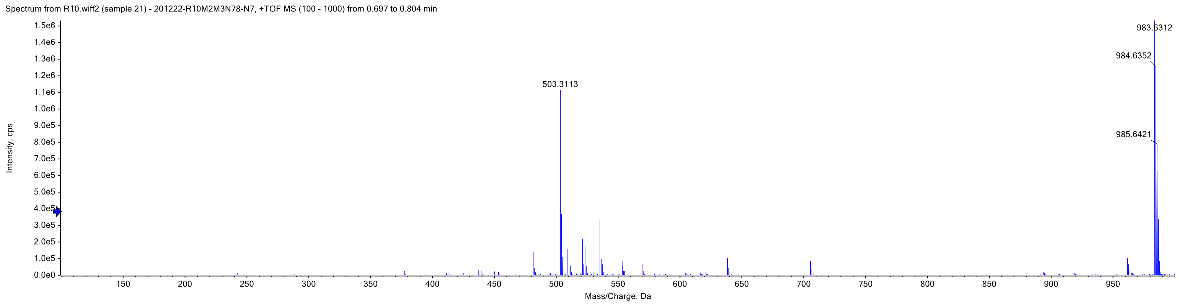

Figure S-49. HRMS spectrum of compound **7**.

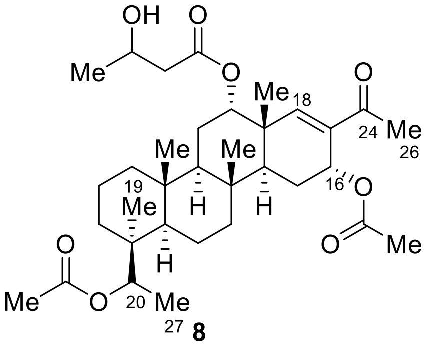

8

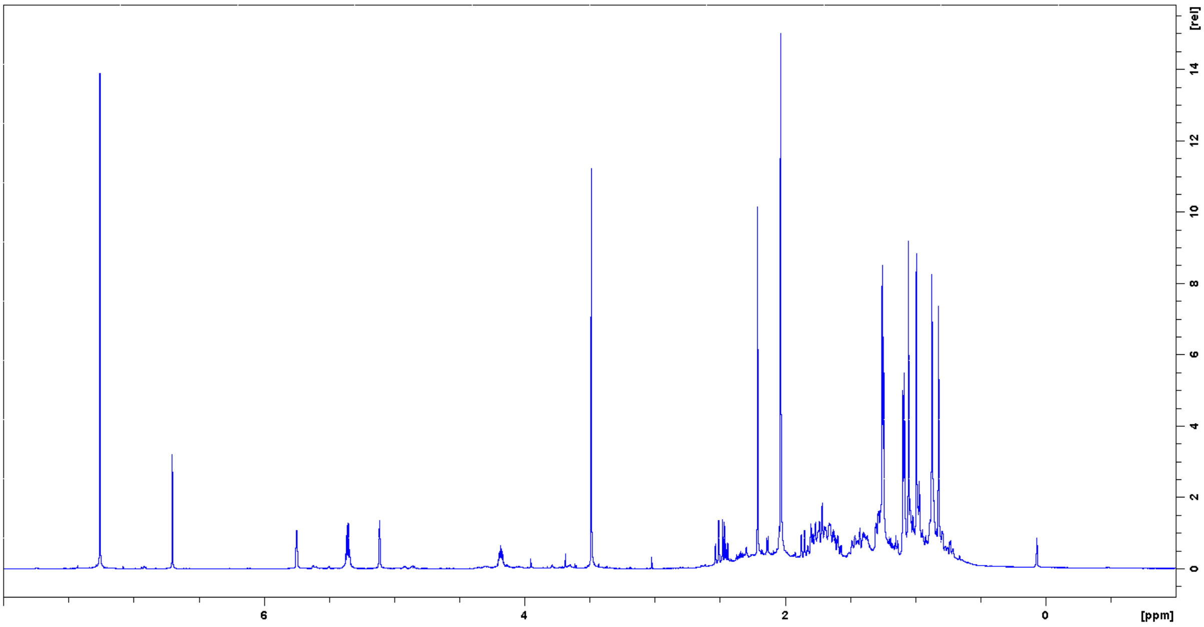

Figure S-50. ^{1}H NMR spectrum of compound **8** ($CDCl_3$, 600 MHz).

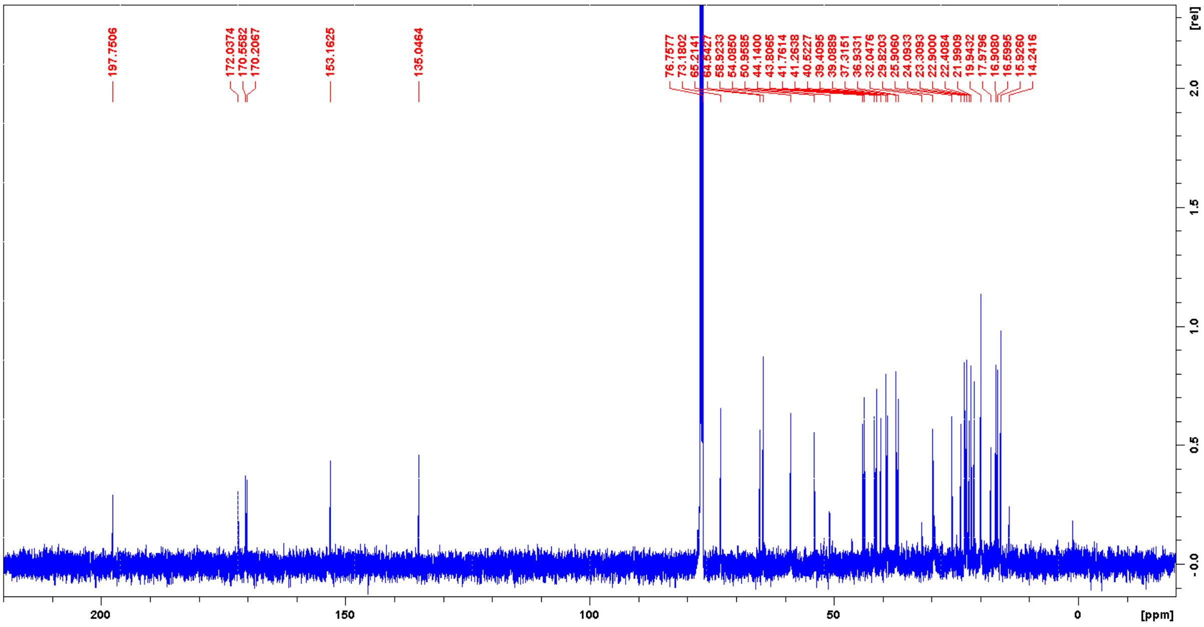

Figure S-51. ^{13}C NMR spectrum of compound **8** ($CDCl_3$, 150 MHz).

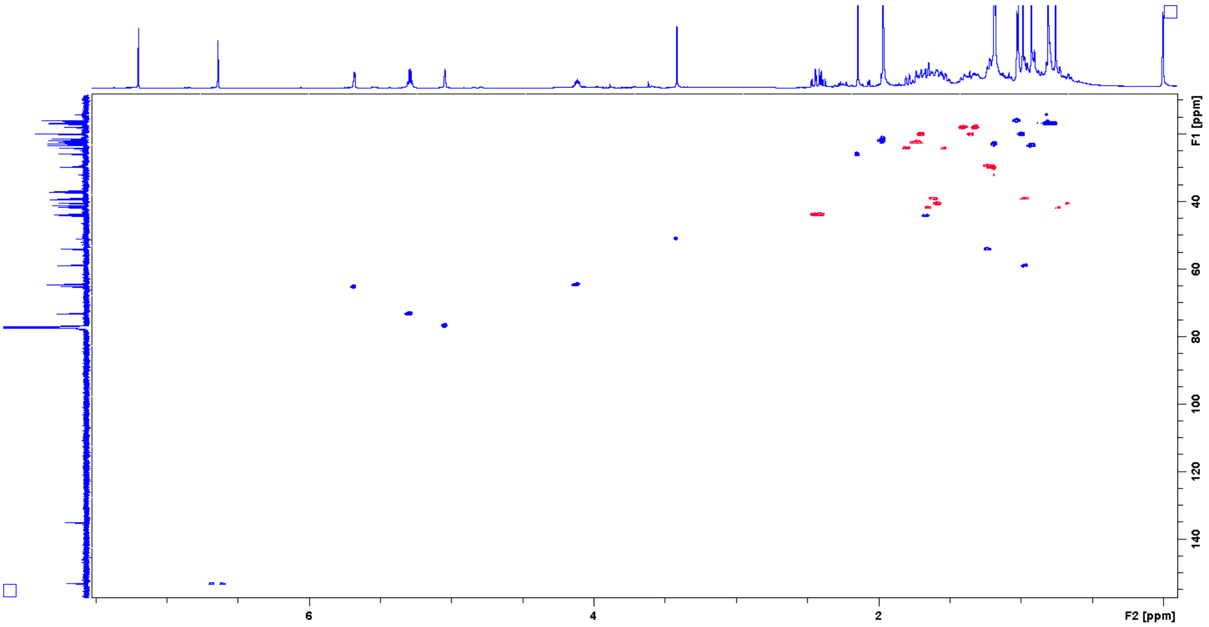

Figure S-52. HSQC spectrum of compound **8** ($CDCl_3$, 600 MHz).

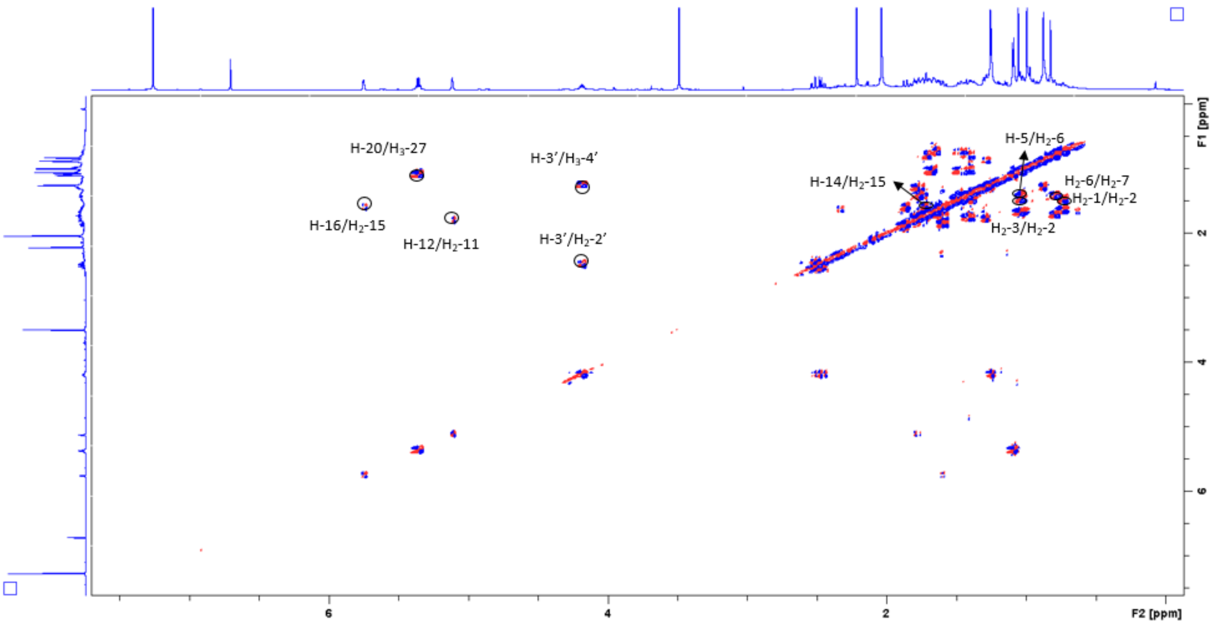

Figure S-53. ^1H-^1H COSY spectrum of compound **8** ($CDCl_3$, 600 MHz).

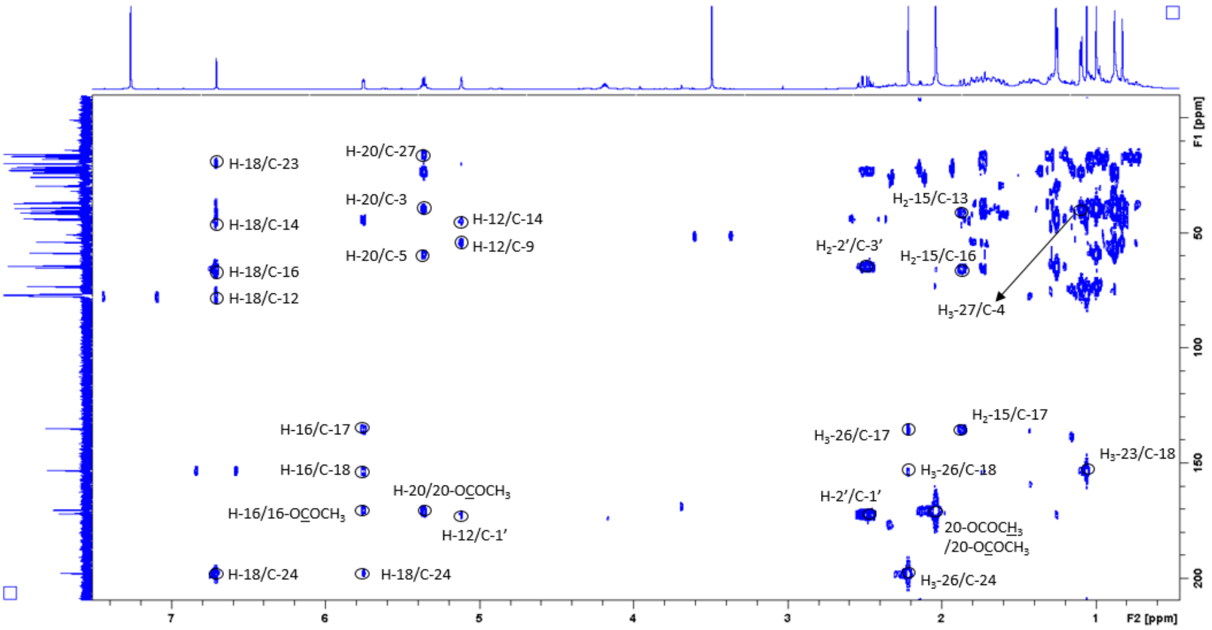

Figure S-54. HMBC spectrum of compound **8** ($CDCl_3$, 600 MHz).

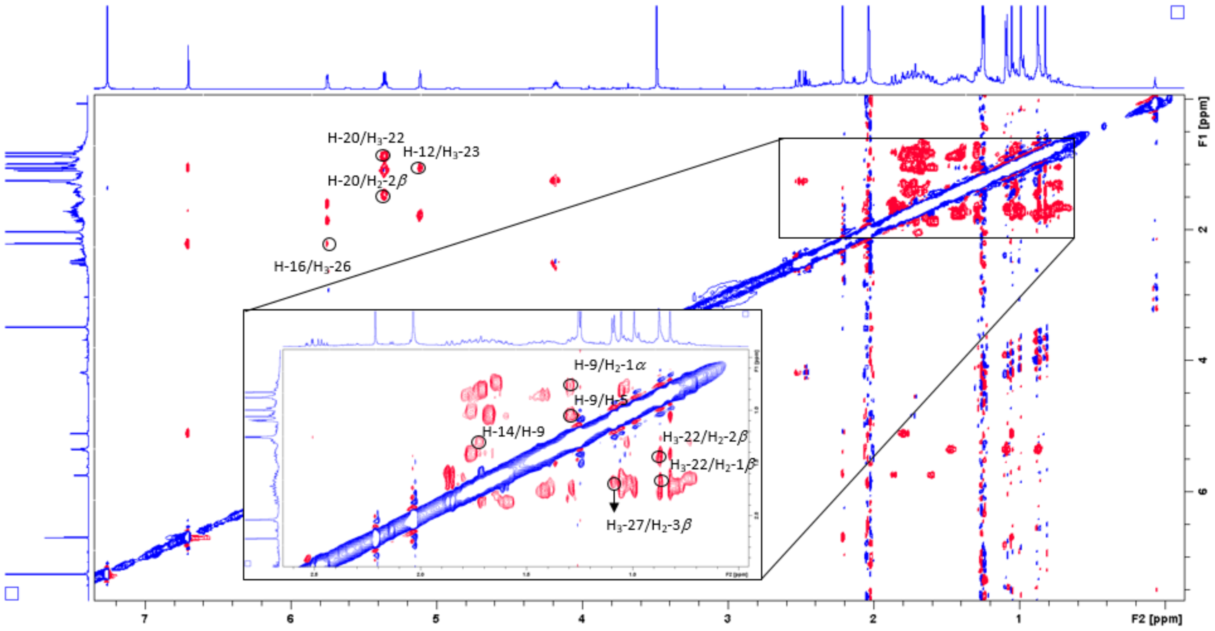

Figure S-55. NOESY spectrum of compound **8** ($CDCl_3$, 600 MHz).

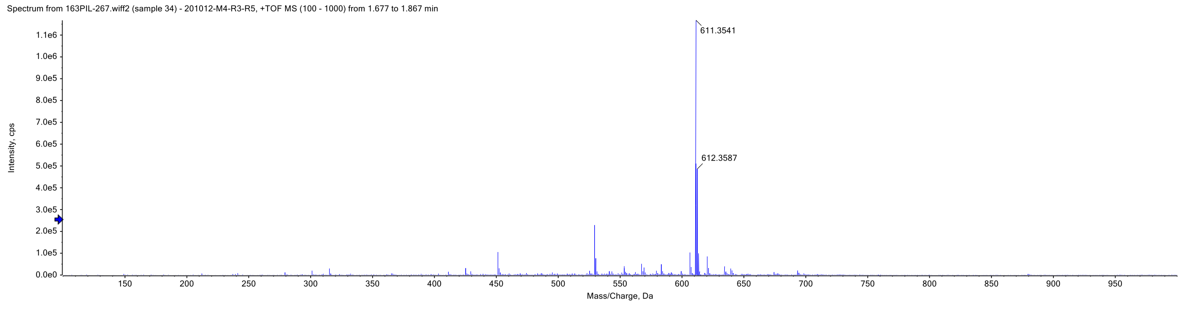

Figure S-56. HRMS of compound **8**.

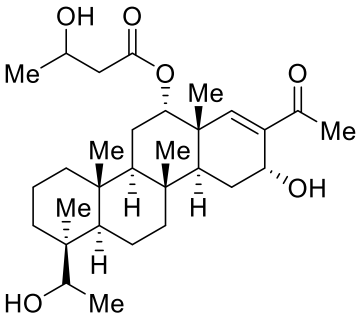

9

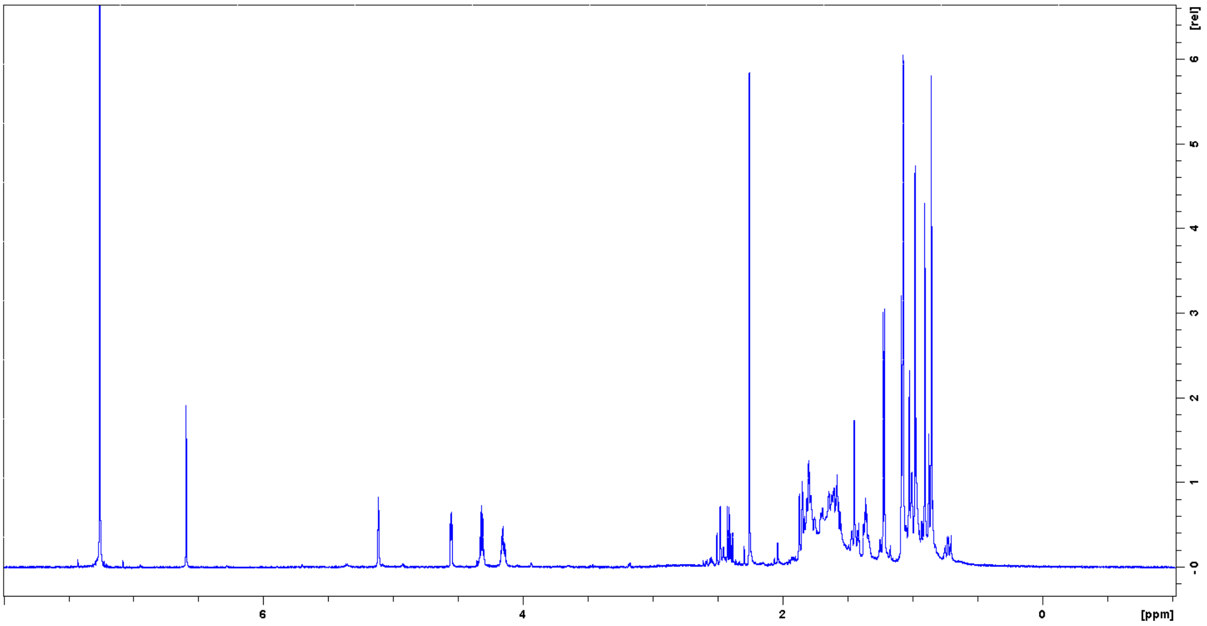

Figure S-57. ^{1}H NMR spectrum of compound **9** ($CDCl_3$, 600 MHz).

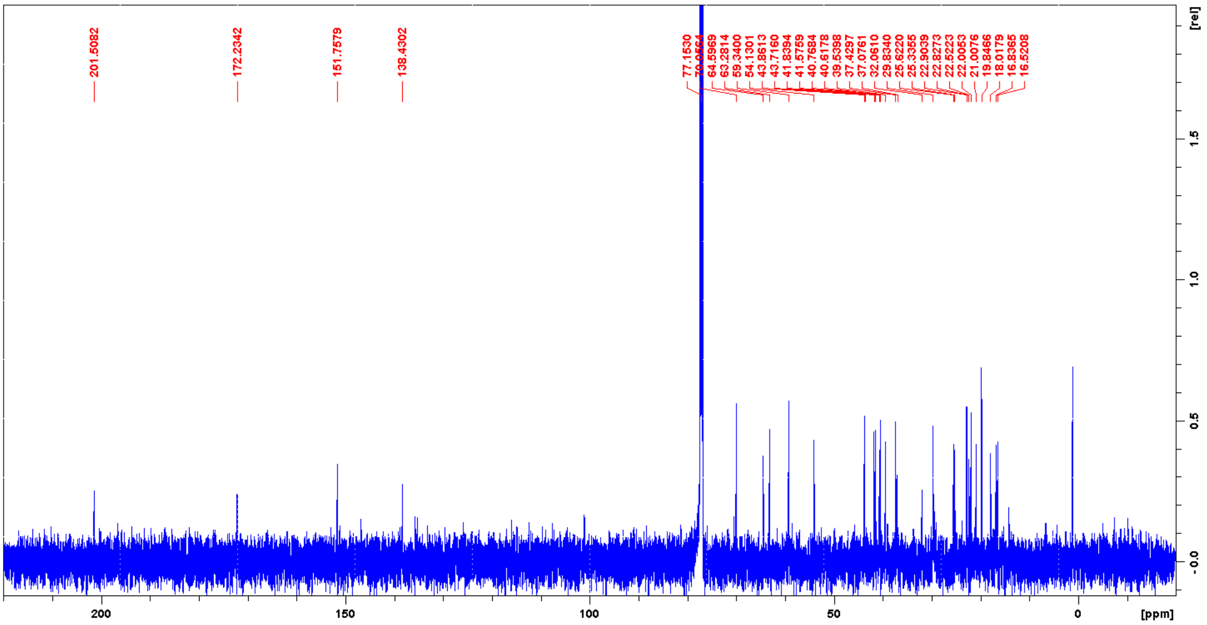

Figure S-58. ^{13}C NMR spectrum of compound **9** ($CDCl_3$, 150 MHz).

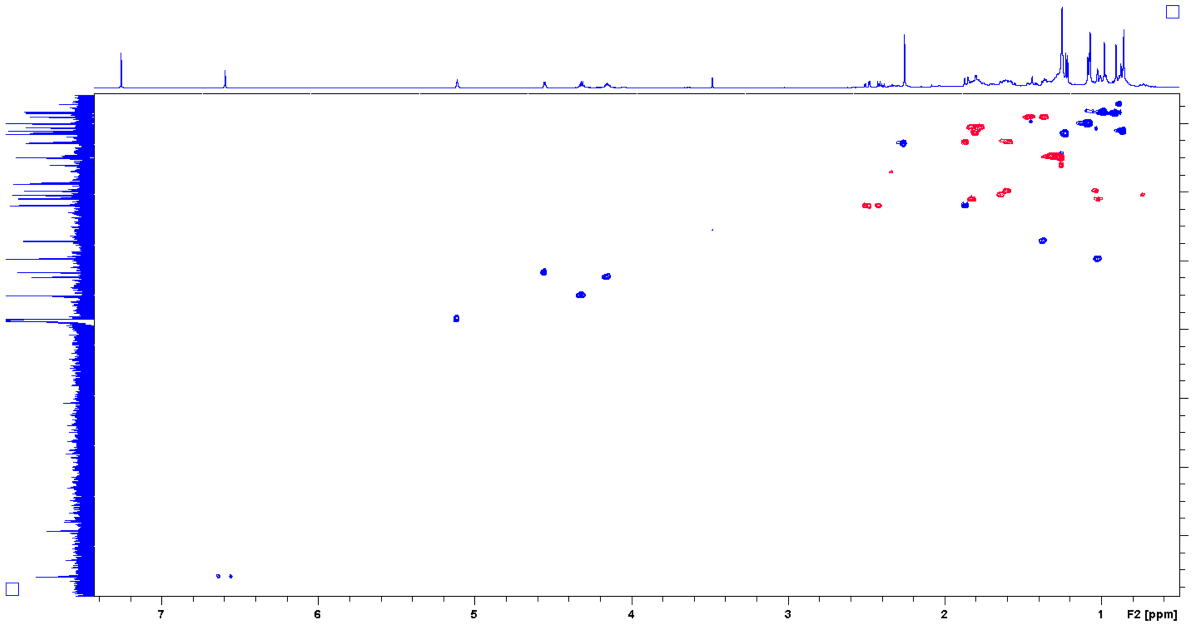

Figure S-59. HSQC spectrum of compound **9** ($CDCl_3$, 600 MHz).

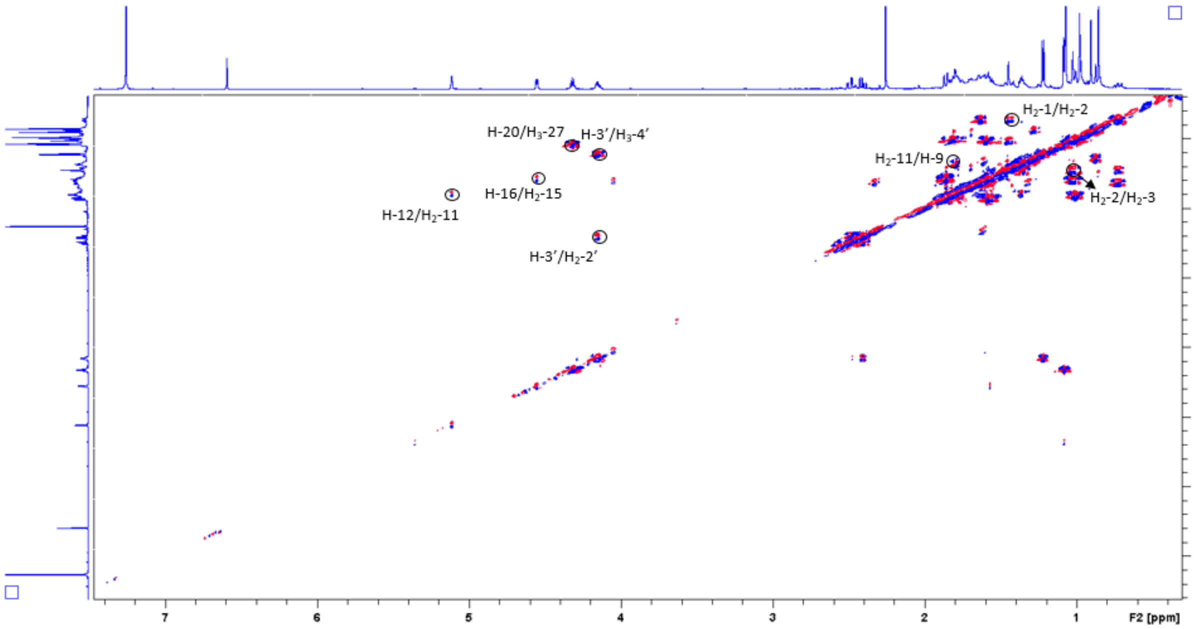

Figure S-60. ^1H-^1H COSY spectrum of compound **9** ($CDCl_3$, 600 MHz).

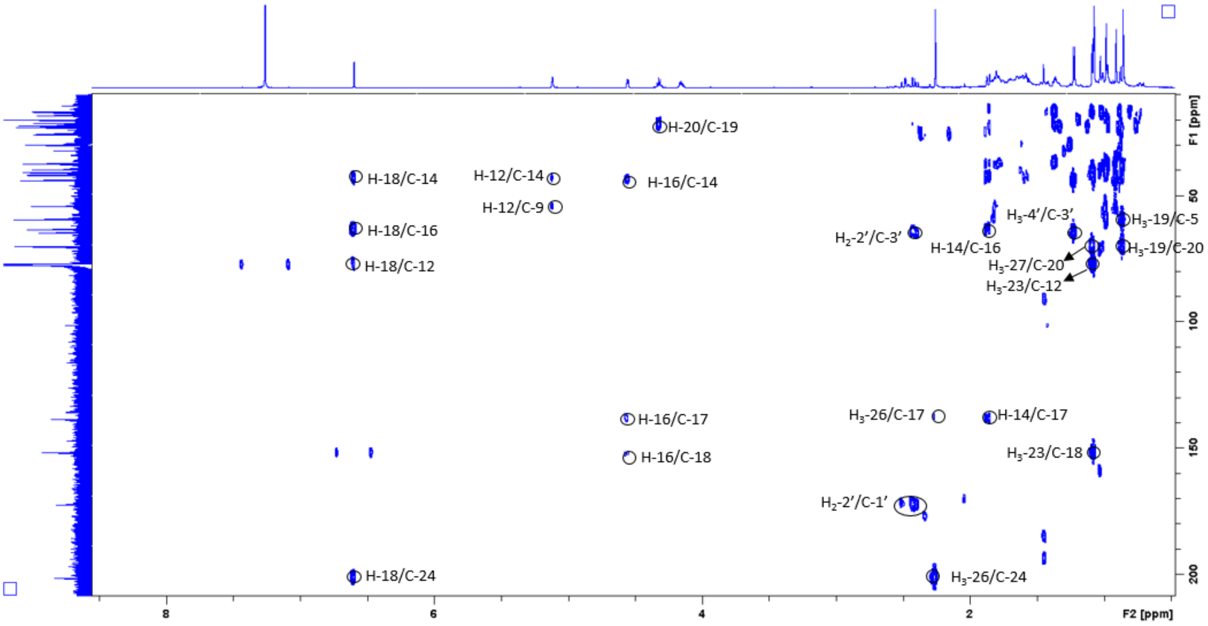

Figure S-61. HMBC spectrum of compound **9** ($CDCl_3$, 600 MHz).

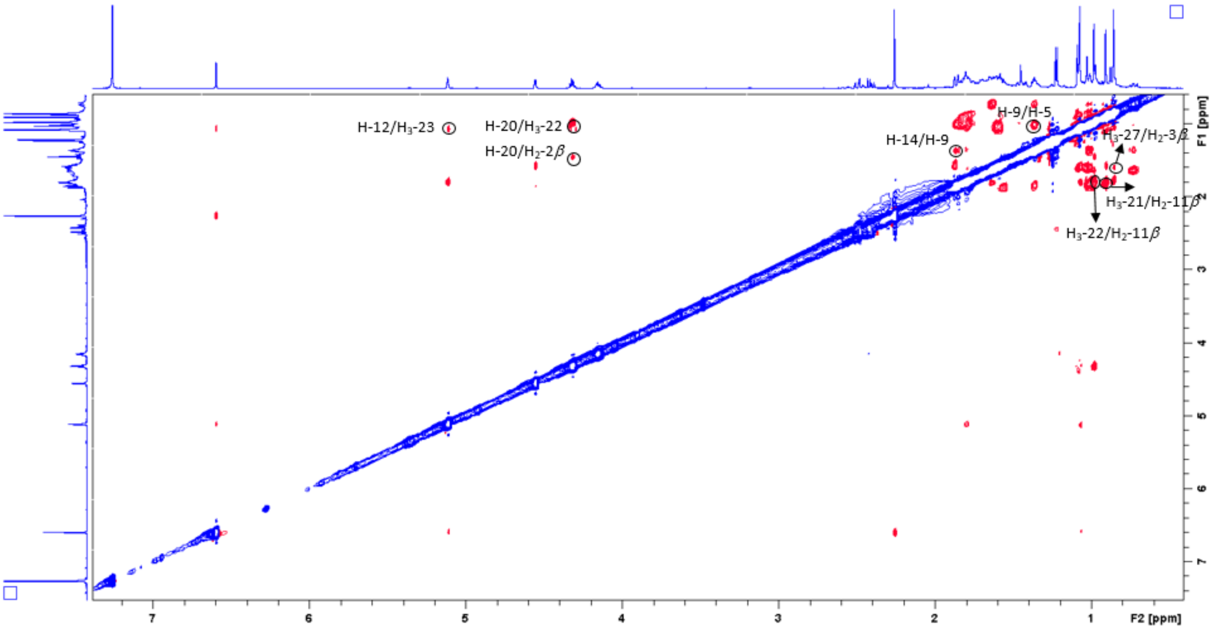

Figure S-62. NOESY spectrum of compound **9** ($CDCl_3$, 600 MHz).

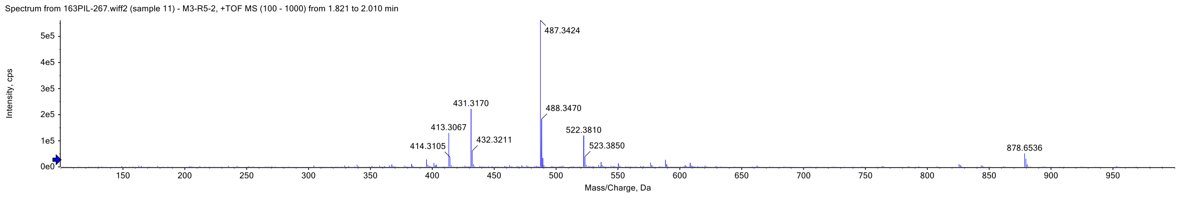

Figure S-63. HRMS spectrum of compound **9**.

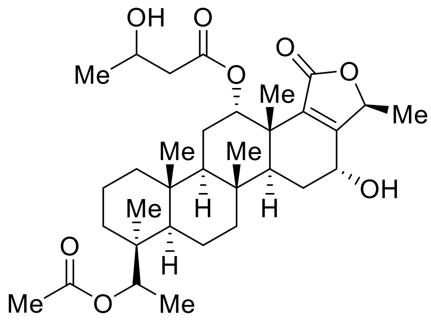

10

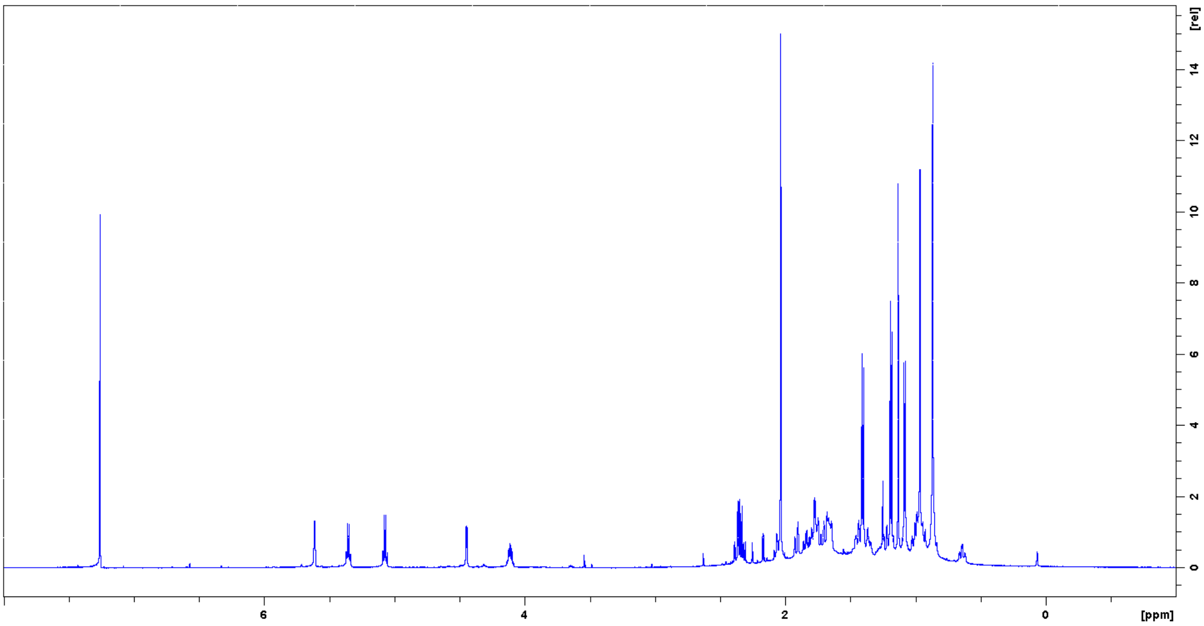

Figure S-64. ^{1}H NMR spectrum of compound **10** ($CDCl_3$, 600 MHz).

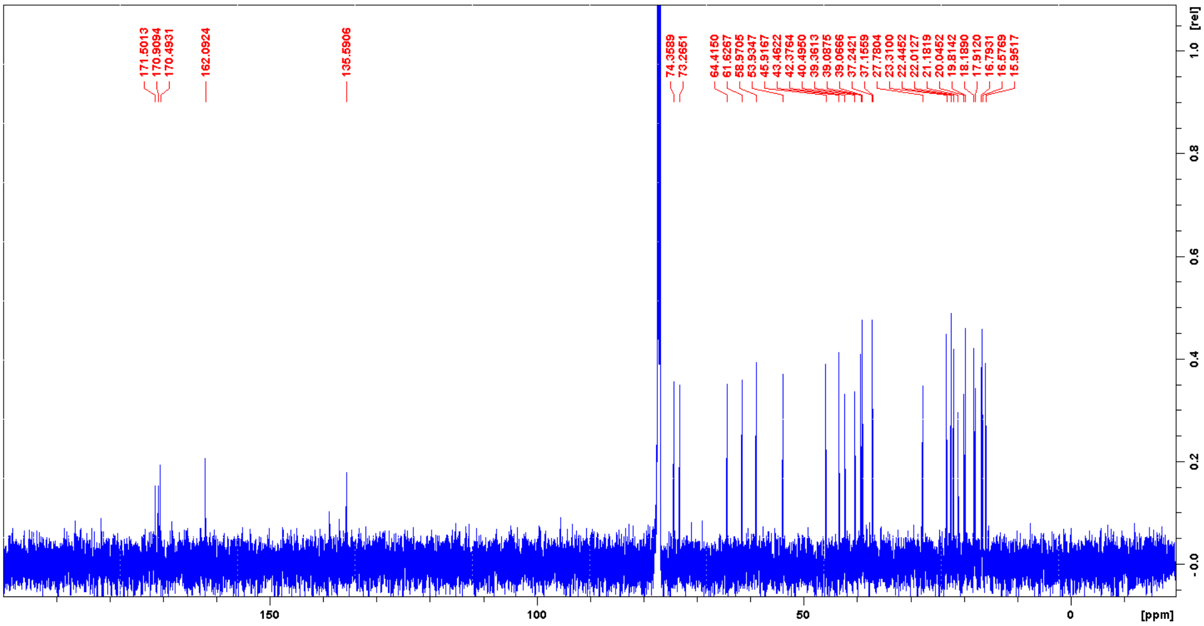

Figure S-65. ^{13}C NMR spectrum of compound **10** ($CDCl_3$, 150 MHz).

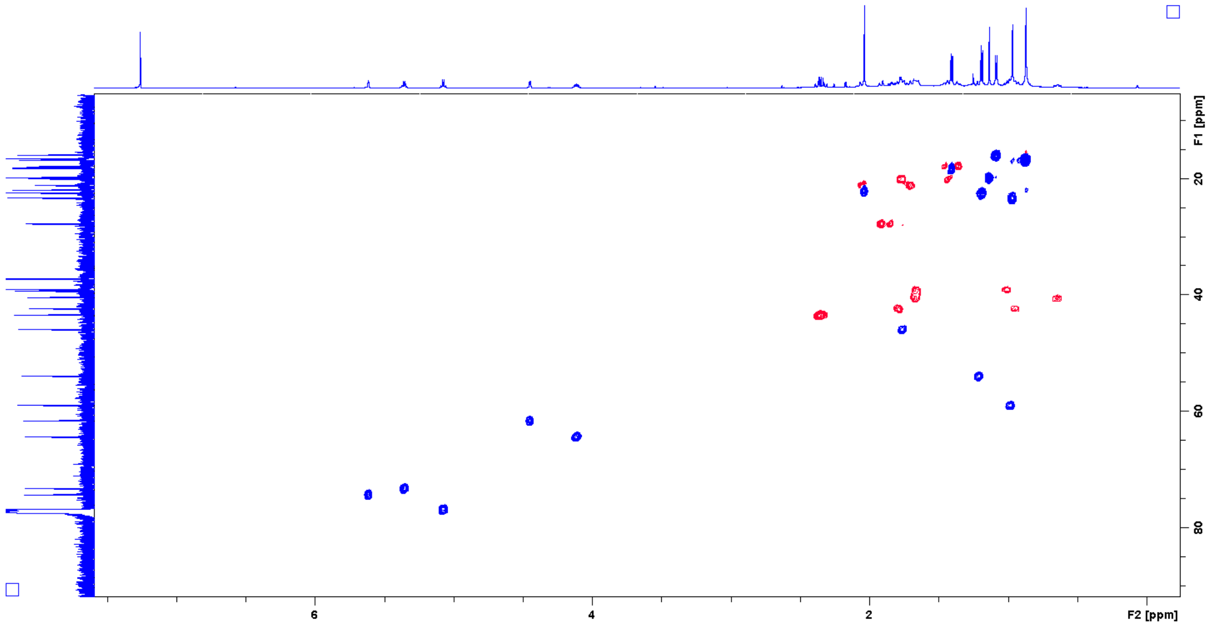

Figure S-66. HSQC spectrum of compound **10** ($CDCl_3$, 600 MHz).

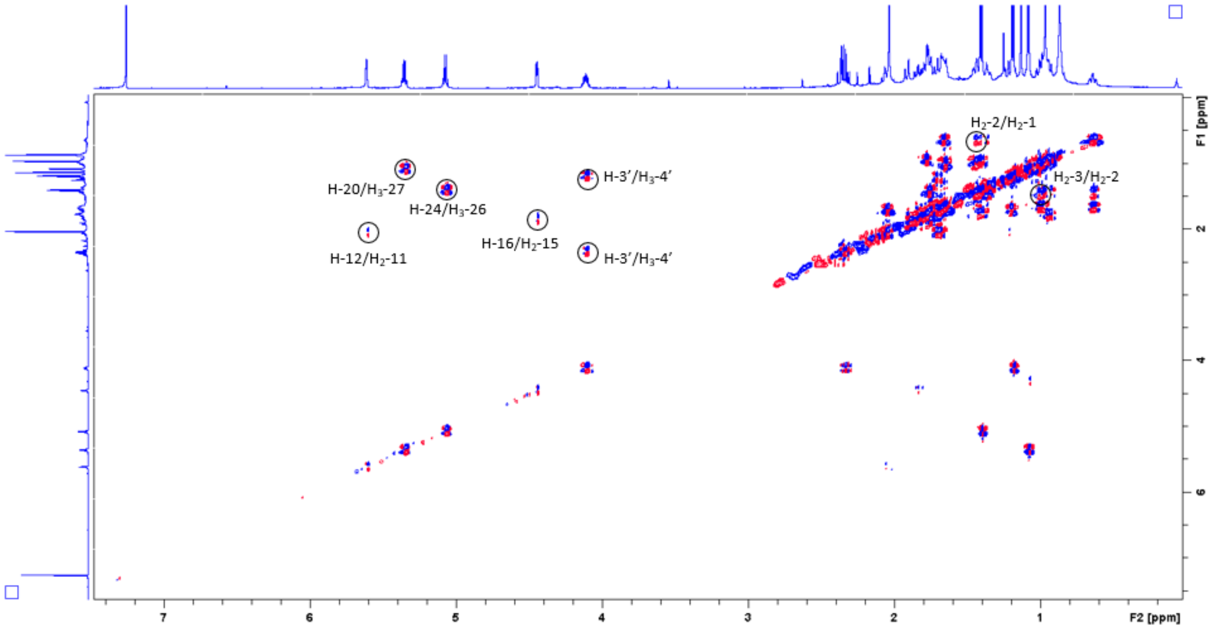

Figure S-67. ^1H-^1H COSY spectrum of compound **10** ($CDCl_3$, 600 MHz).

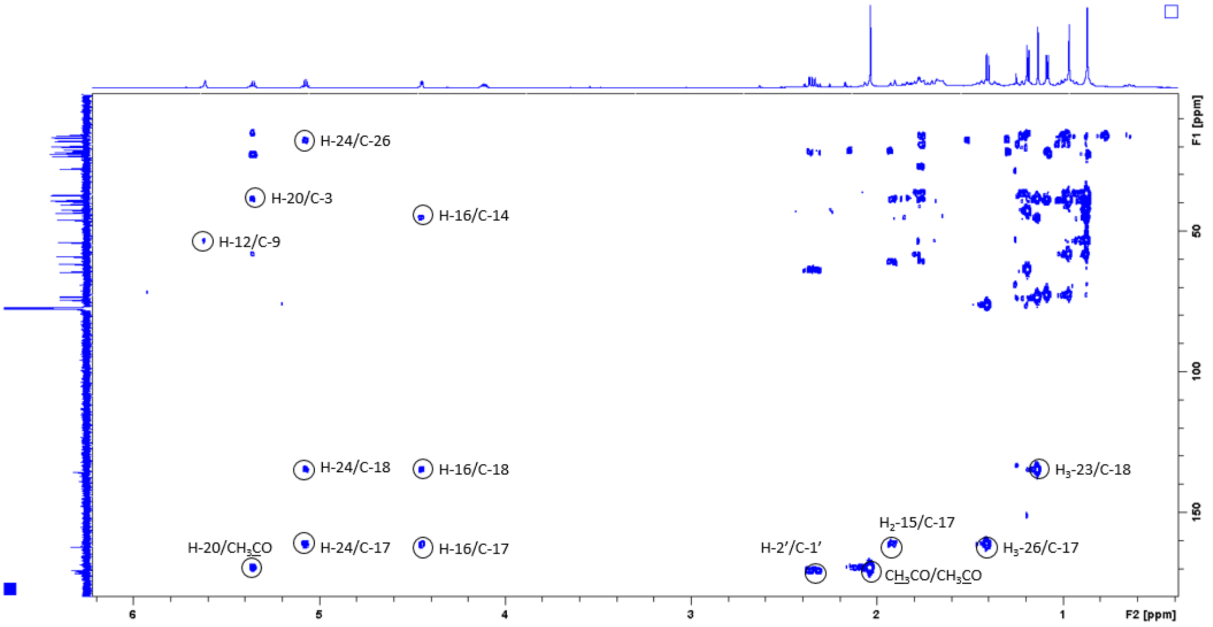

Figure S-68. HMBC spectrum of compound **10** ($CDCl_3$, 600 MHz).

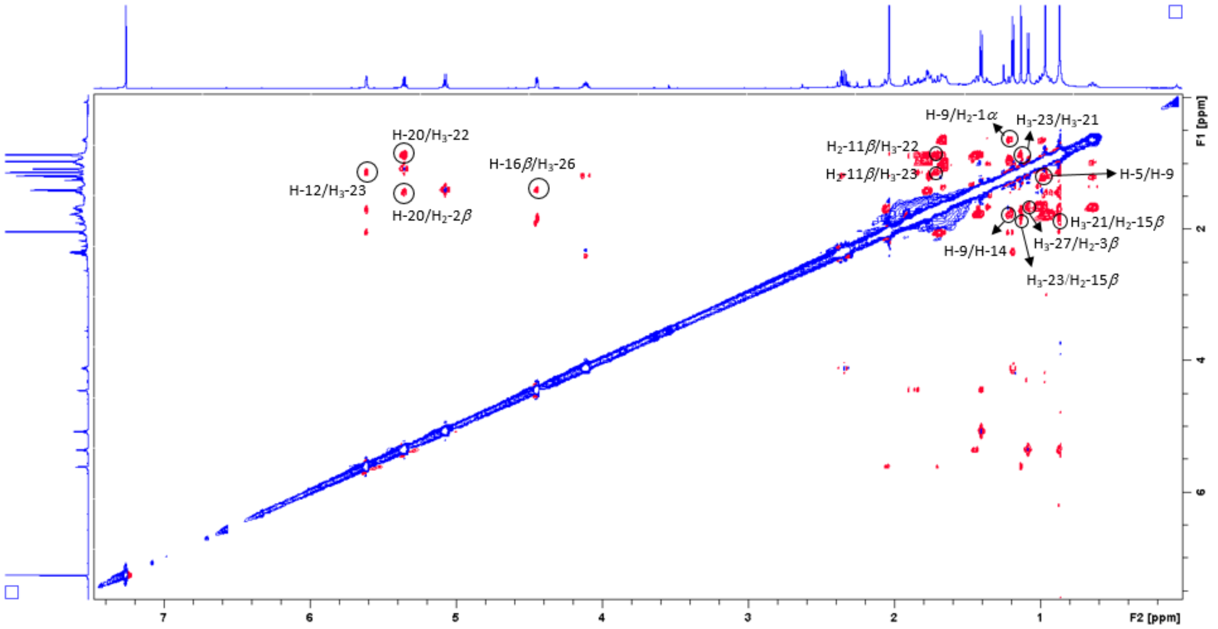

Figure S-69. NOESY spectrum of compound **10** ($CDCl_3$, 600 MHz).

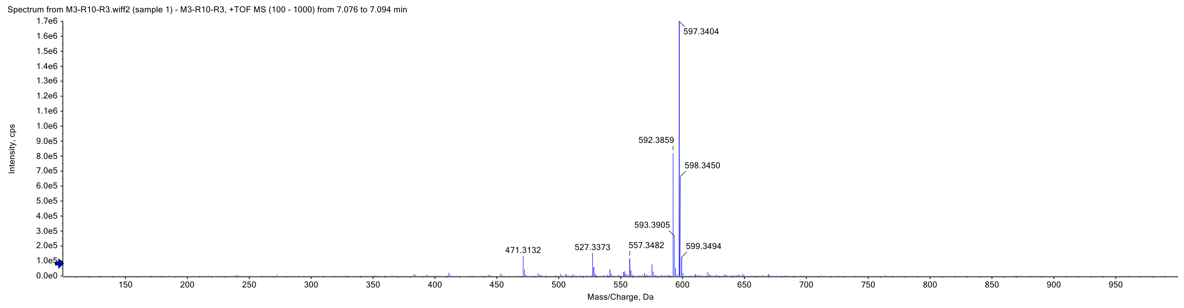

Figure S-70. HRMS spectrum of compound **10**.

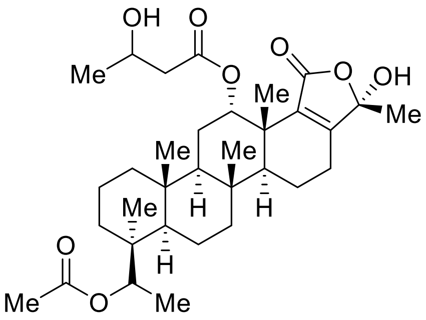

11

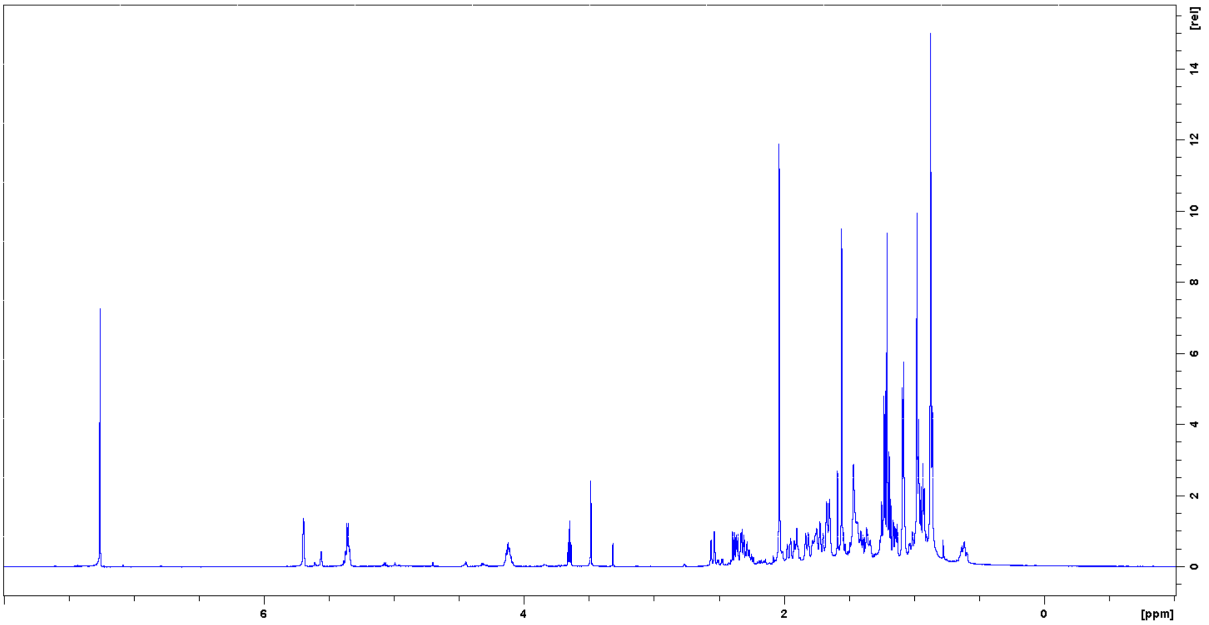

Figure S-71. ^{1}H NMR spectrum of compound **11** ($CDCl_3$, 600 MHz).

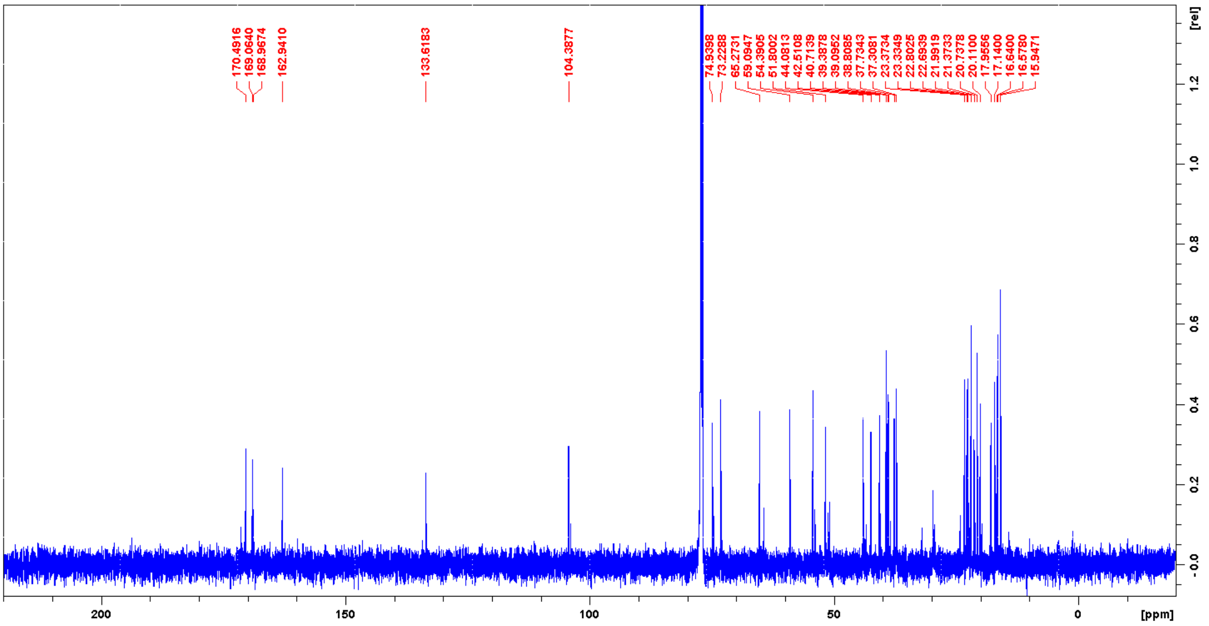

Figure S-72. ^{13}C NMR spectrum of compound **11** ($CDCl_3$, 150 MHz).

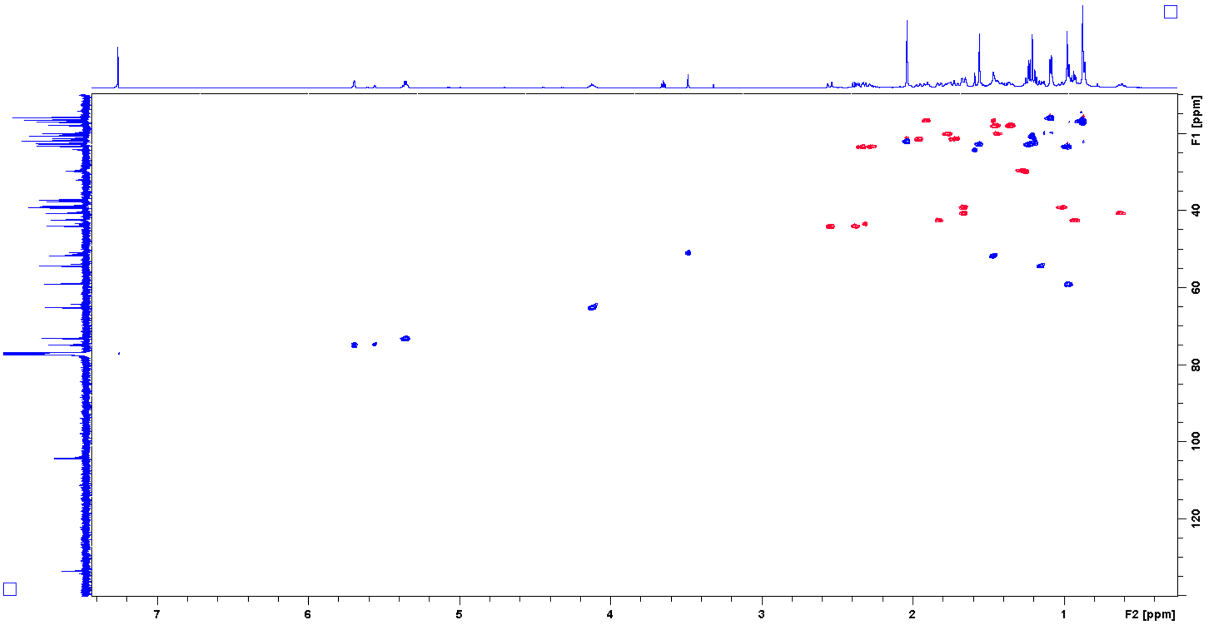

Figure S-73. HSQC spectrum of compound **11** ($CDCl_3$, 600 MHz).

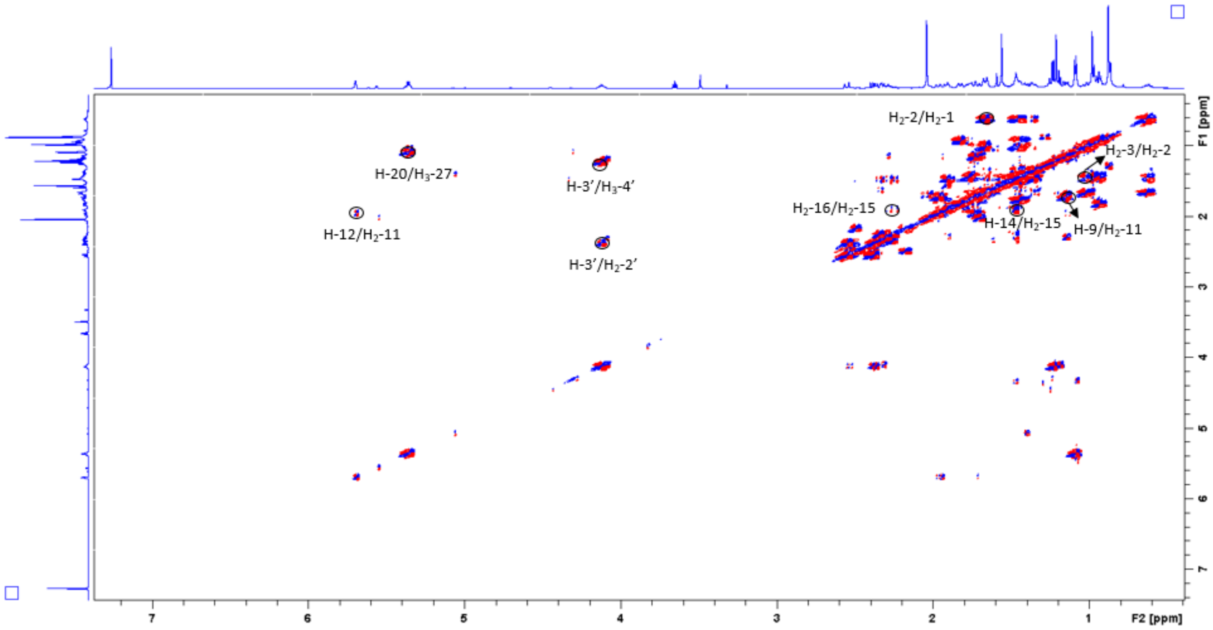

Figure S-74. ^{1}H-^{1}H COSY spectrum of compound **11** ($CDCl_3$, 600 MHz).

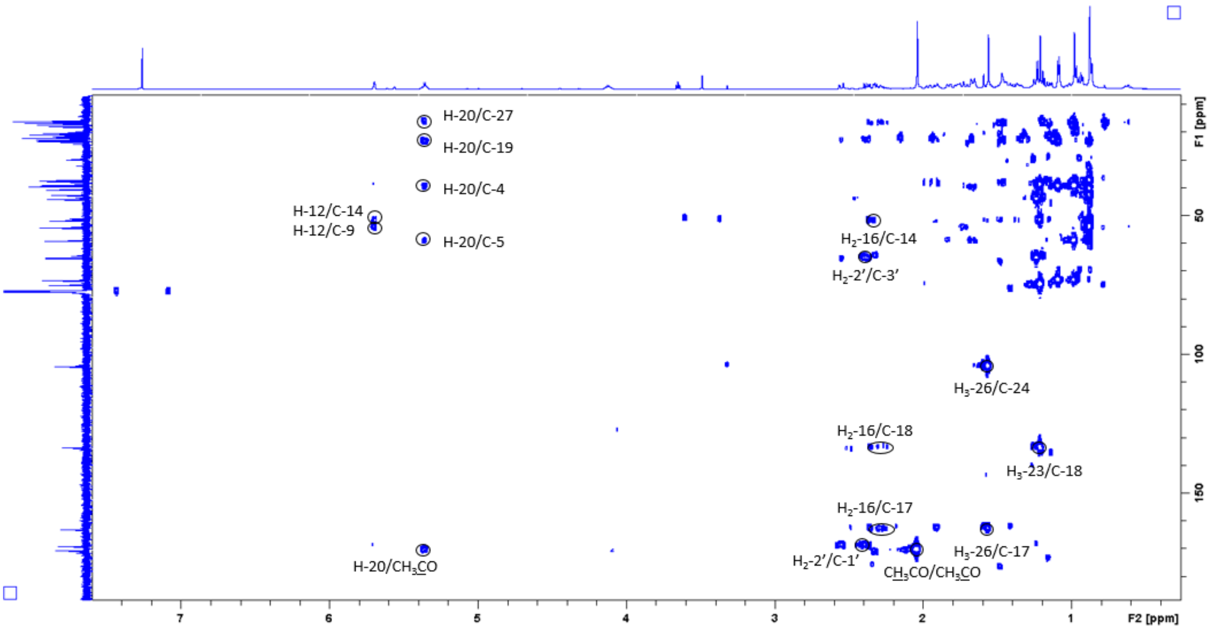

Figure S-75. HMBC spectrum of compound **11** ($CDCl_3$, 600 MHz).

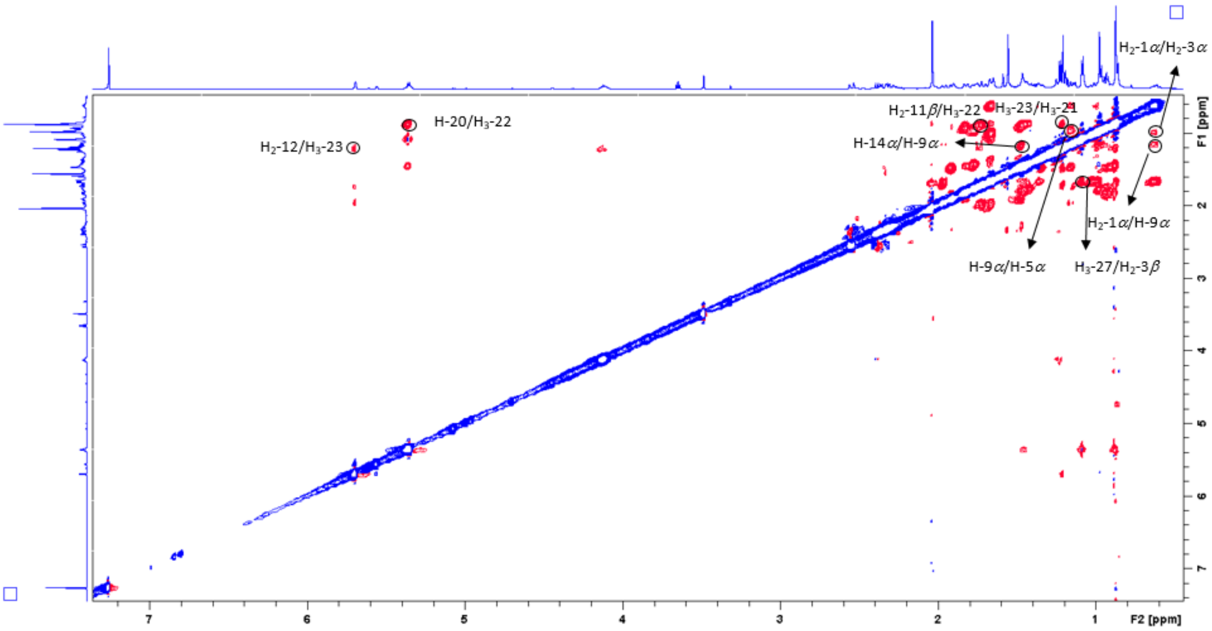

Figure S-76. NOESY spectrum of compound **11** ($CDCl_3$, 600 MHz).

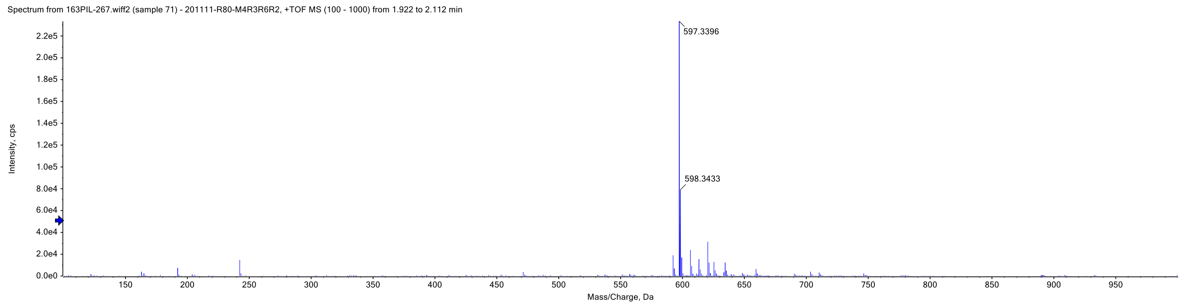

Figure S-77. HRMS spectrum of compound **11**.

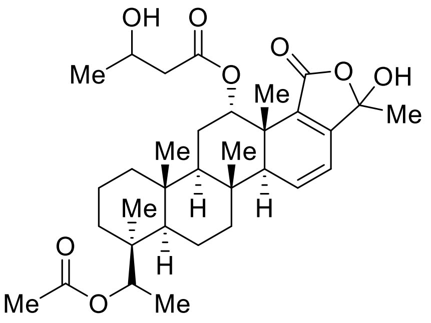

12

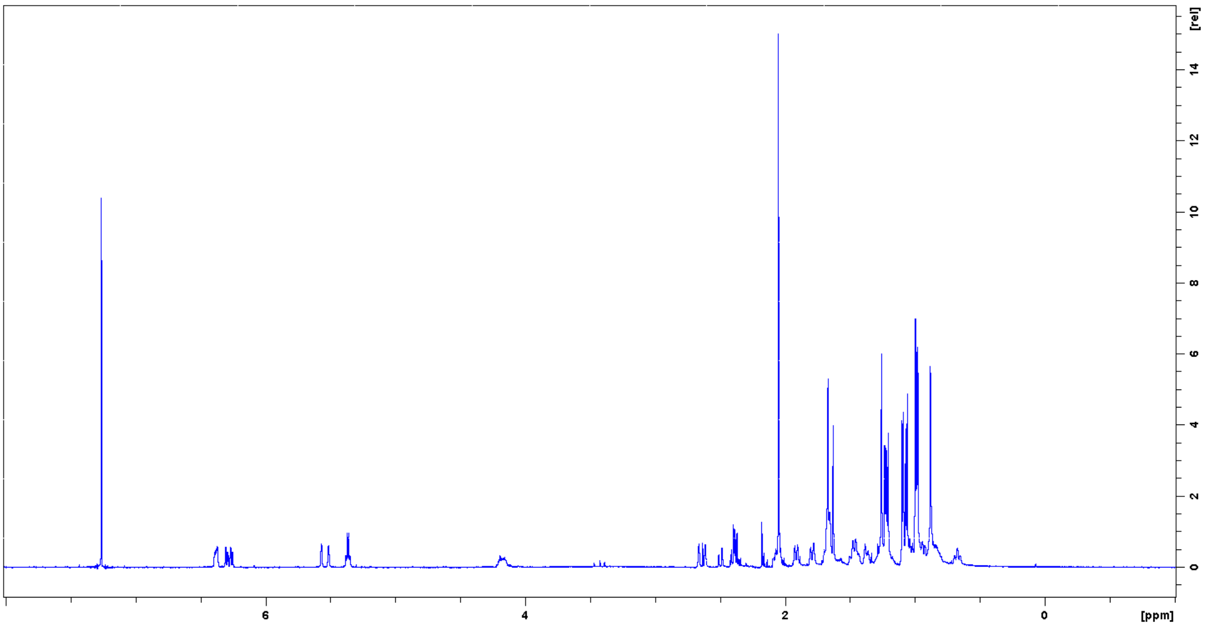

Figure S-78. ^{1}H NMR spectrum of compound **12** ($CDCl_3$, 600 MHz).

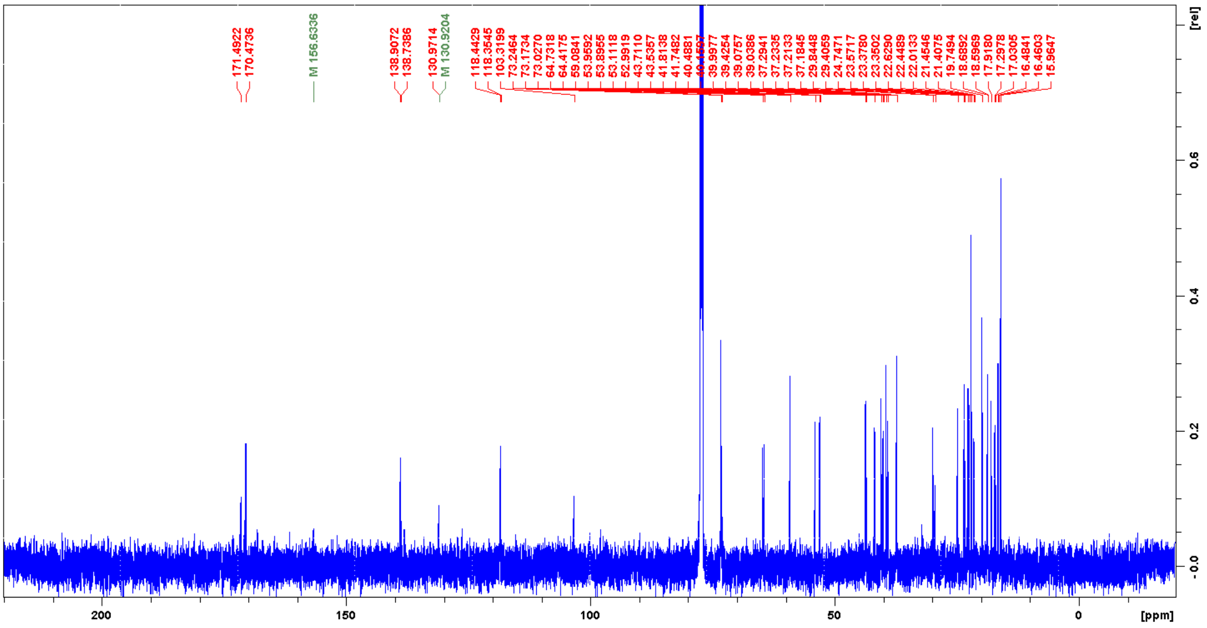

Figure S-79. ^{13}C NMR spectrum of compound **12** ($CDCl_3$, 150 MHz).

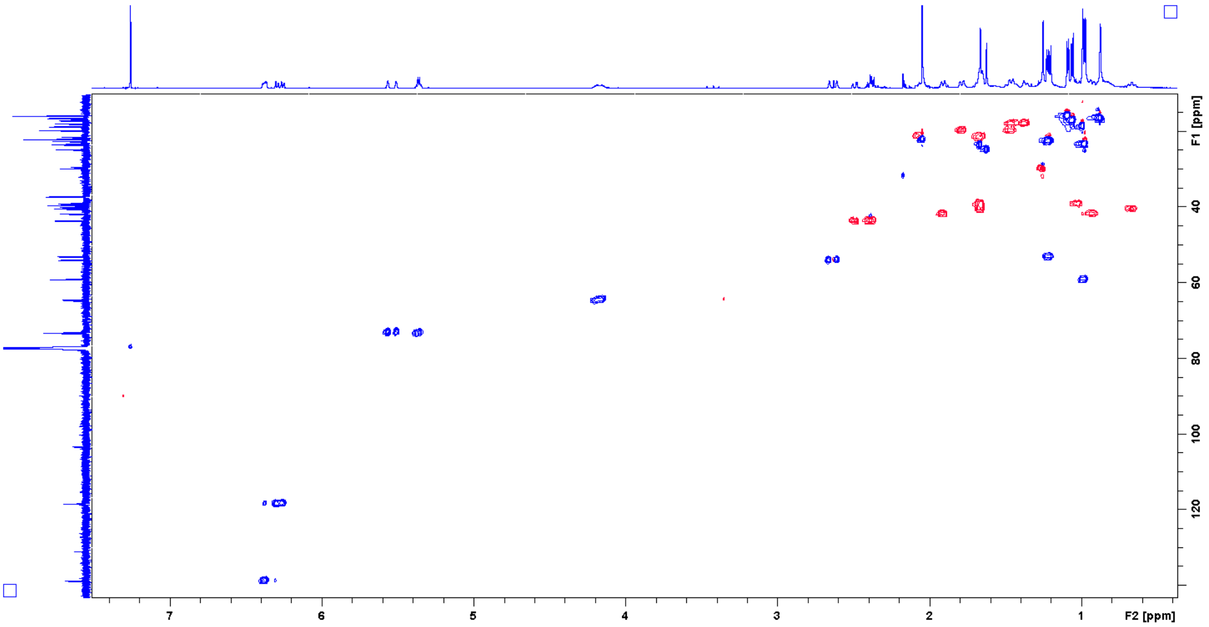

Figure S-80. HSQC spectrum of compound **12** ($CDCl_3$, 600 MHz).

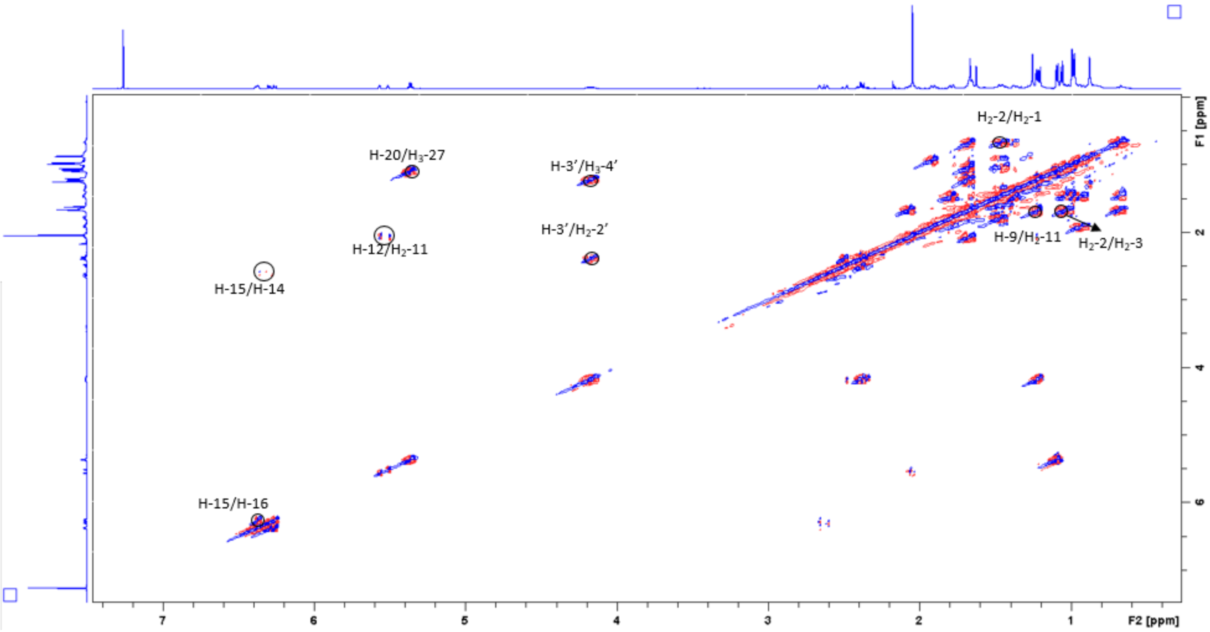

Figure S-81. ^{1}H-^{1}H COSY spectrum of compound **12** ($CDCl_3$, 600 MHz).

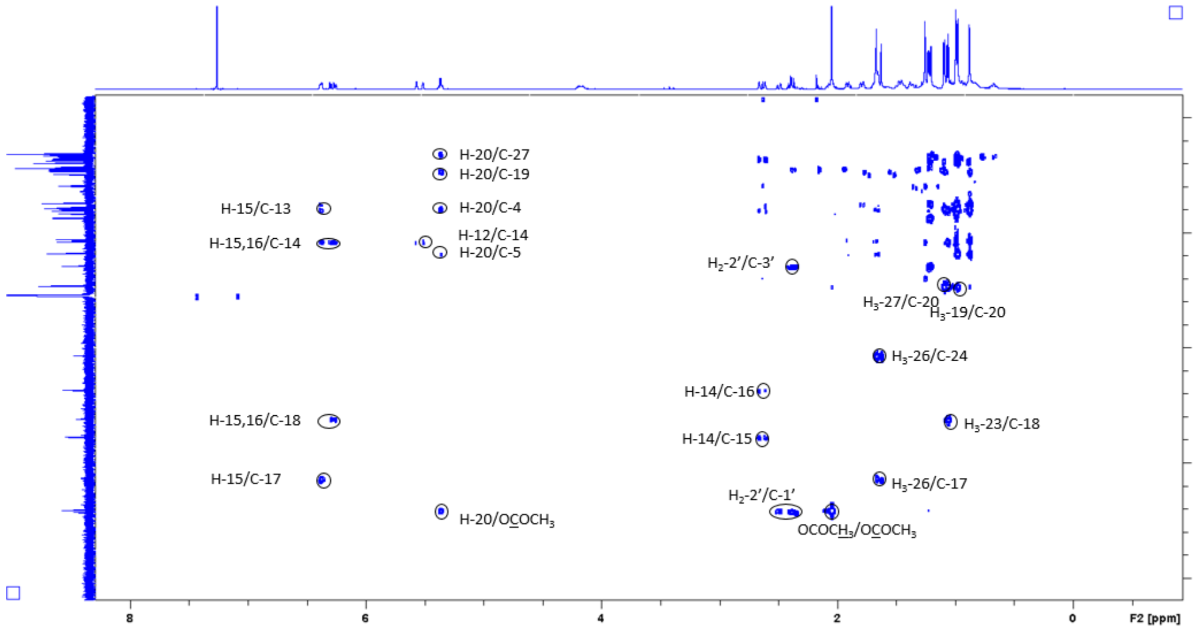

Figure S-82. HMBC spectrum of compound **12** ($CDCl_3$, 600 MHz).

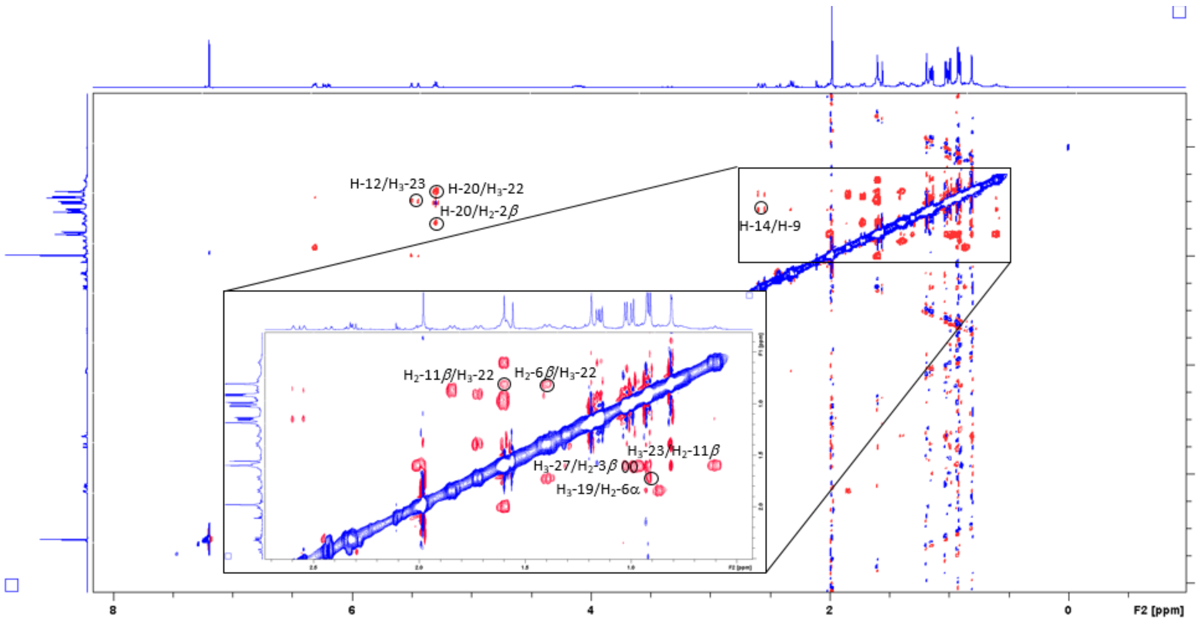

Figure S-83. NOESY spectrum of compound **12** ($CDCl_3$, 600 MHz).

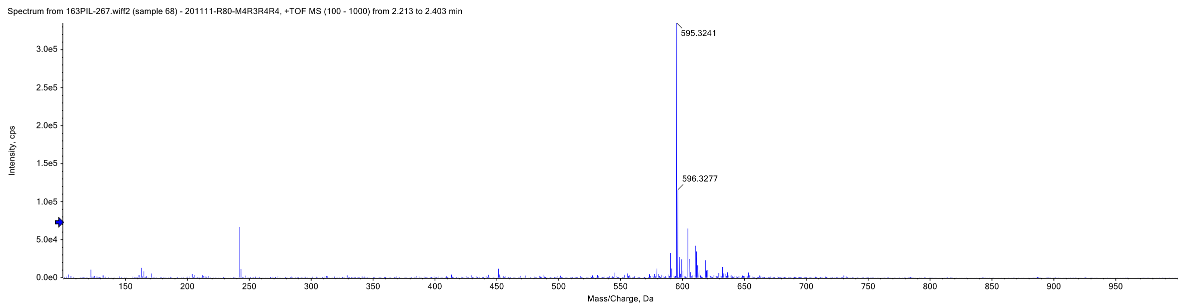

Figure S-84. HRMS spectrum of compound **12**.

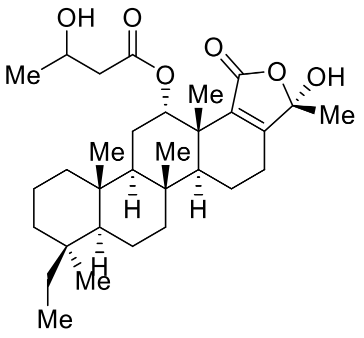

13

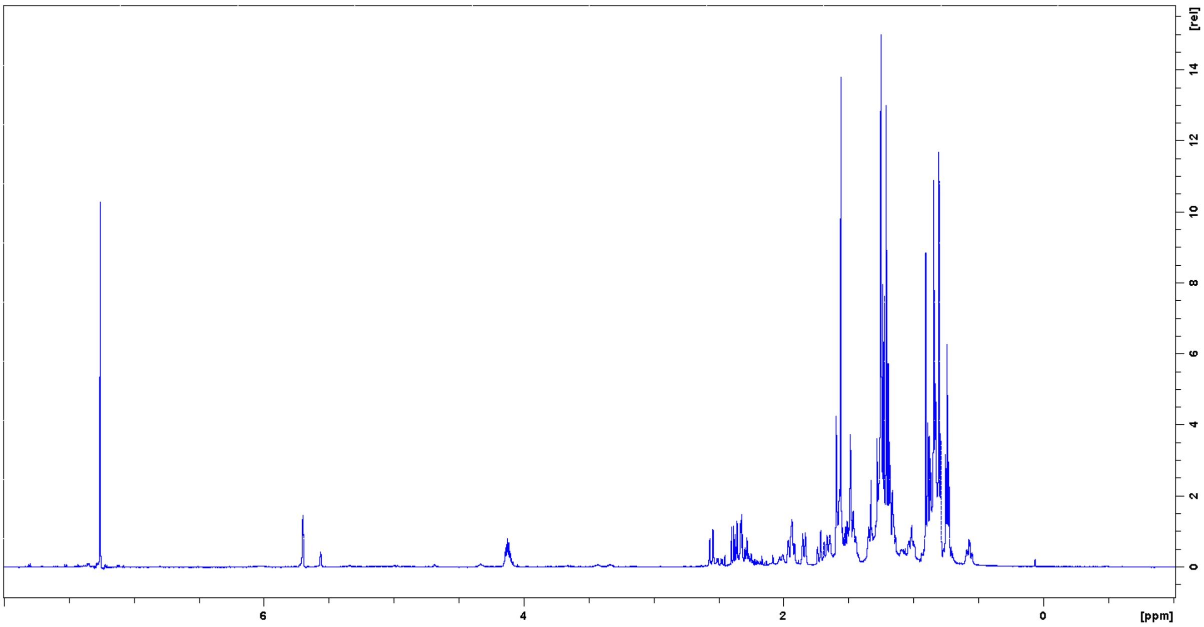

Figure S-85. ^{1}H NMR spectrum of compound **13** ($CDCl_3$, 600 MHz).

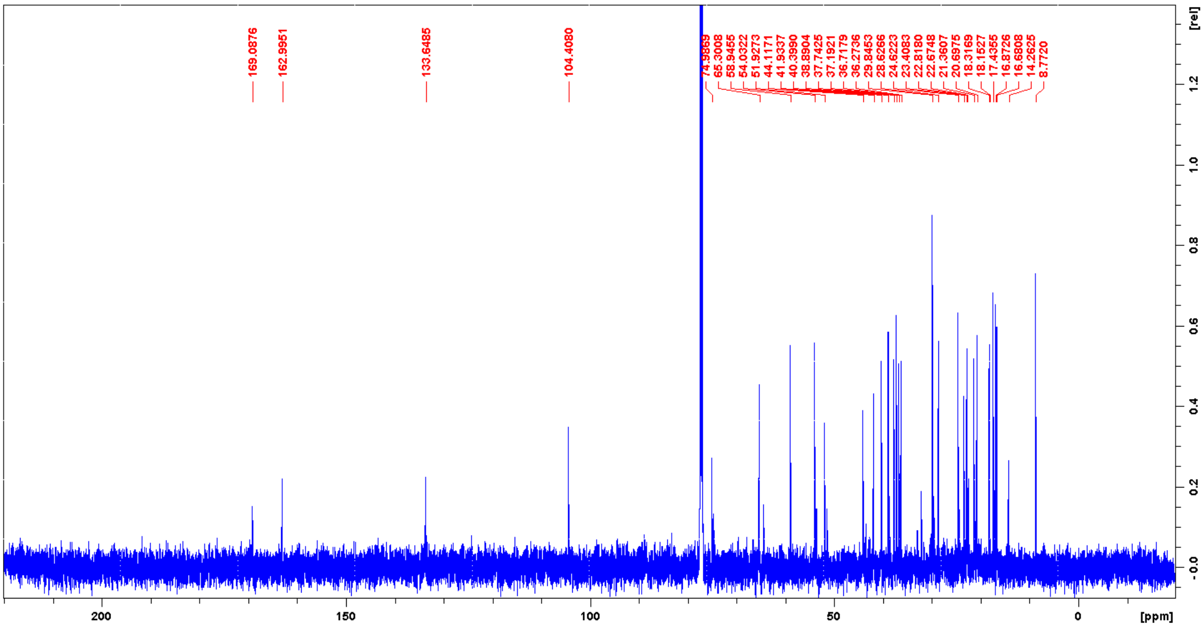

Figure S-86. ^{13}C NMR spectrum of compound **13** ($CDCl_3$, 150 MHz).

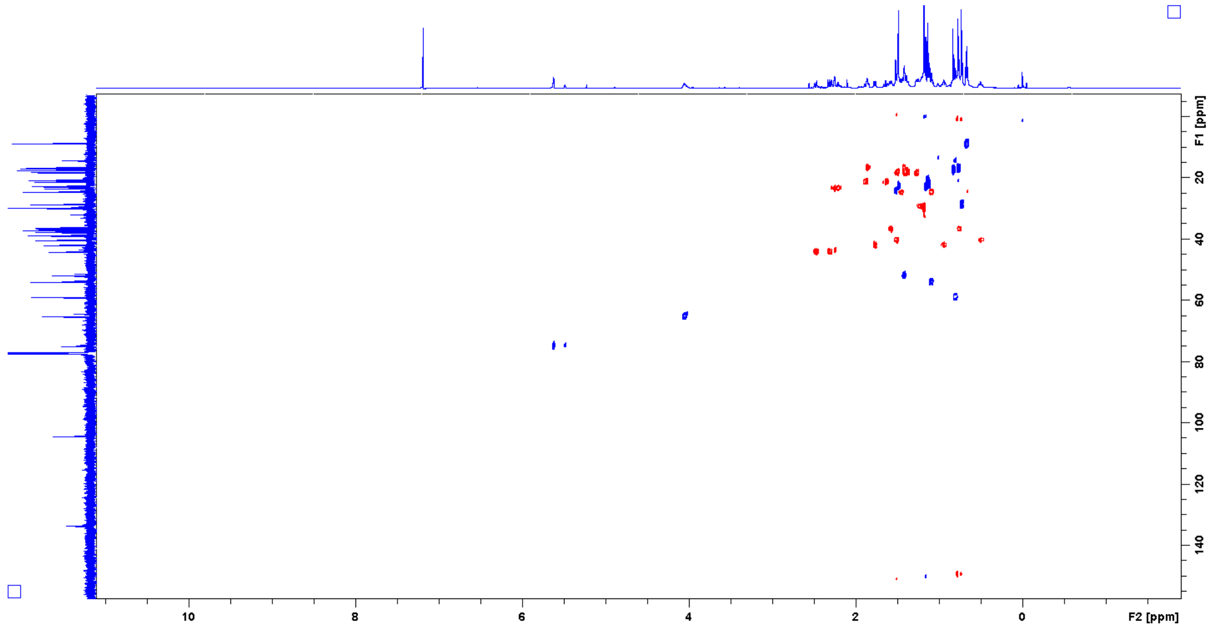

Figure S-87. HSQC spectrum of compound **13** ($CDCl_3$, 600 MHz).

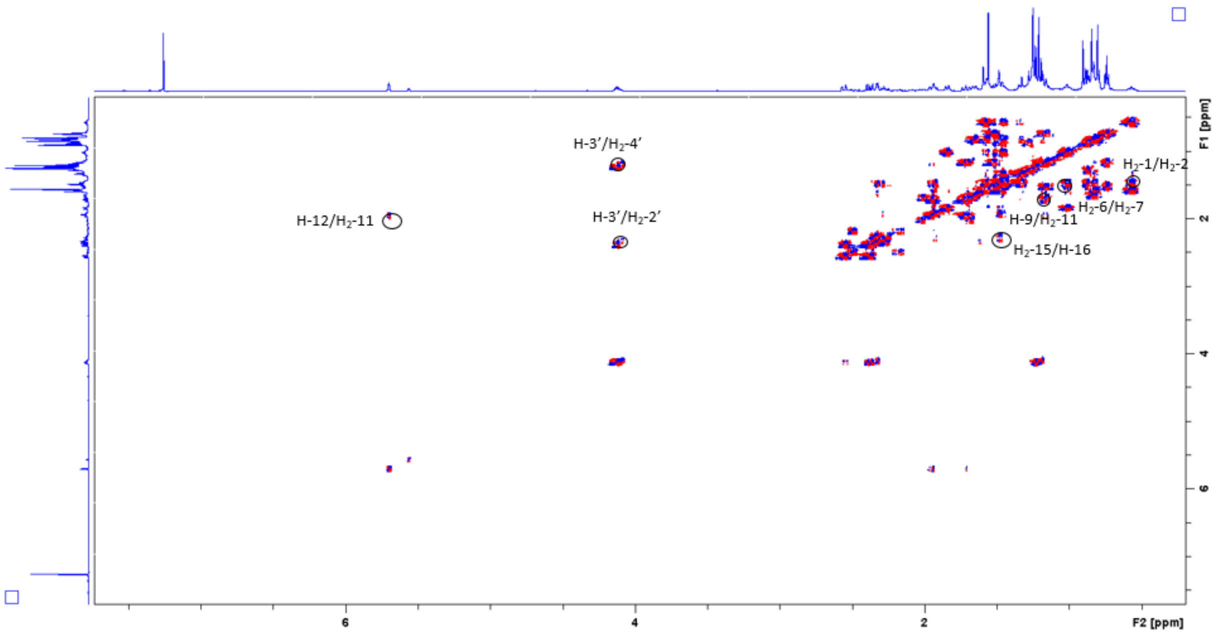

Figure S-88. ^1H-^1H COSY spectrum of compound **13** ($CDCl_3$, 600 MHz).

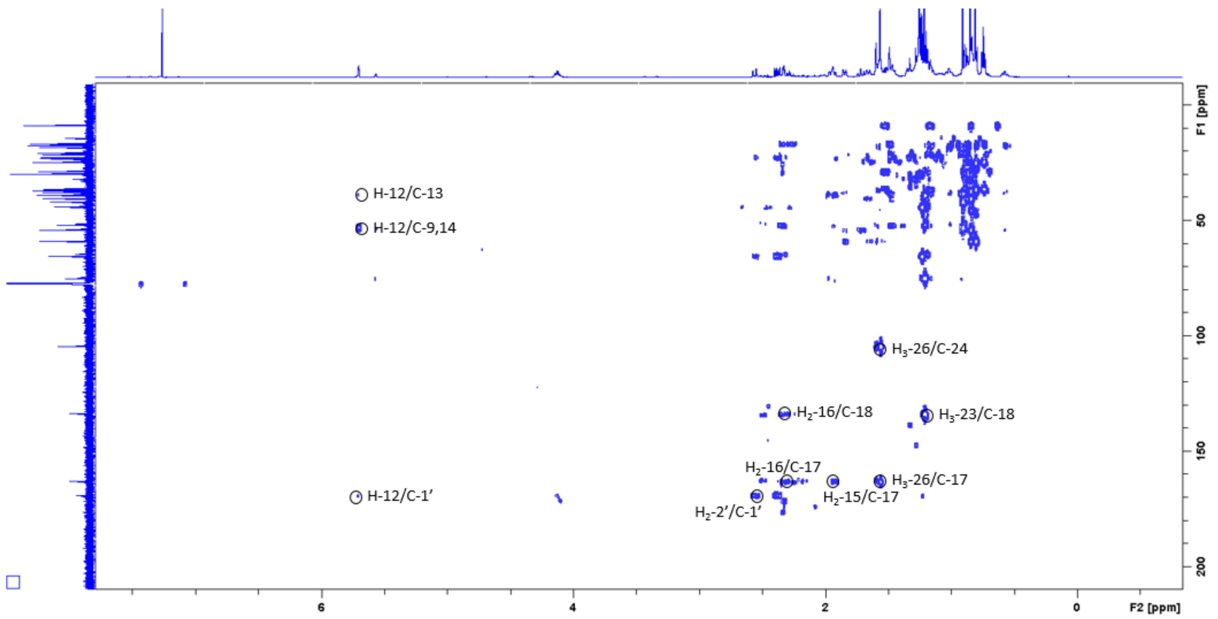

Figure S-89. HMBC spectrum of compound **13** ($CDCl_3$, 600 MHz).

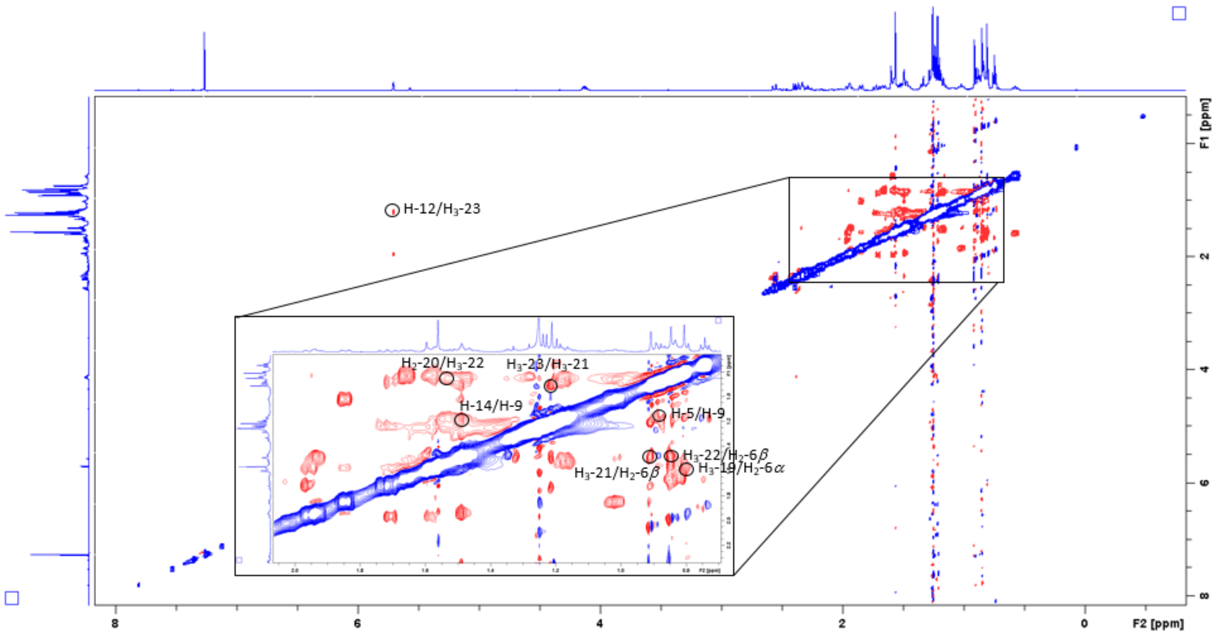

Figure S-90. NOESY spectrum of compound **13** ($CDCl_3$, 600 MHz).

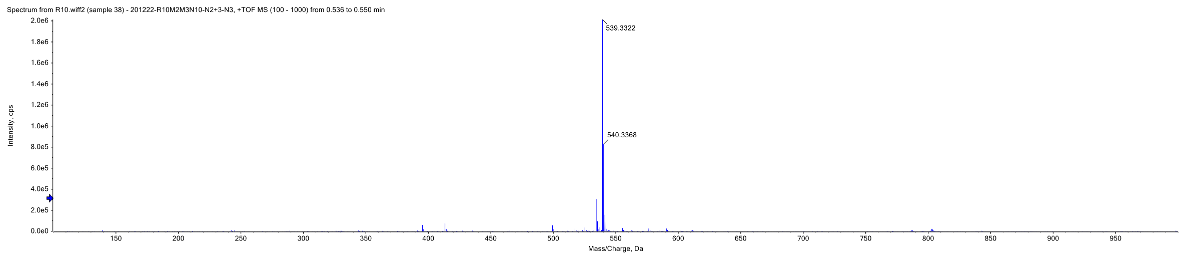

Figure S-91. HRMS spectrum of compound **13**.

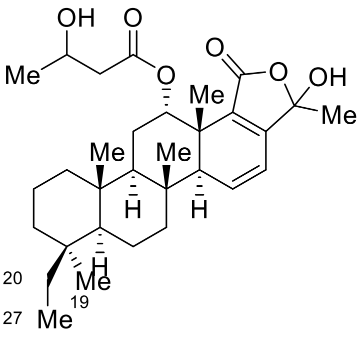

14

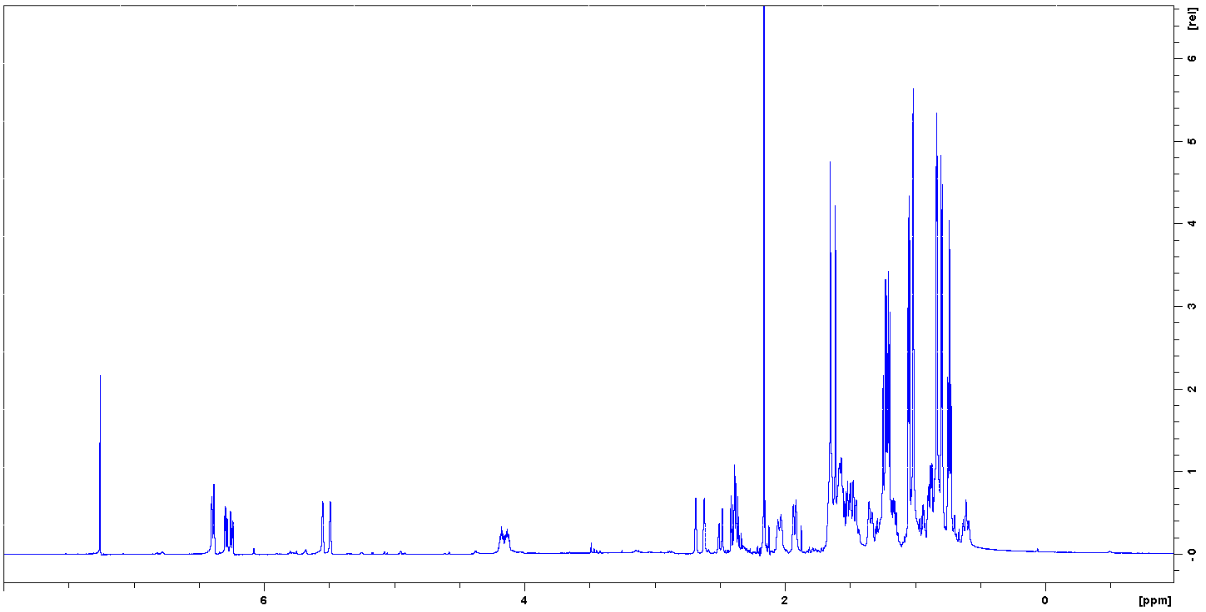

Figure S-92. ^{1}H NMR spectrum of compound **14** ($CDCl_3$, 600 MHz).

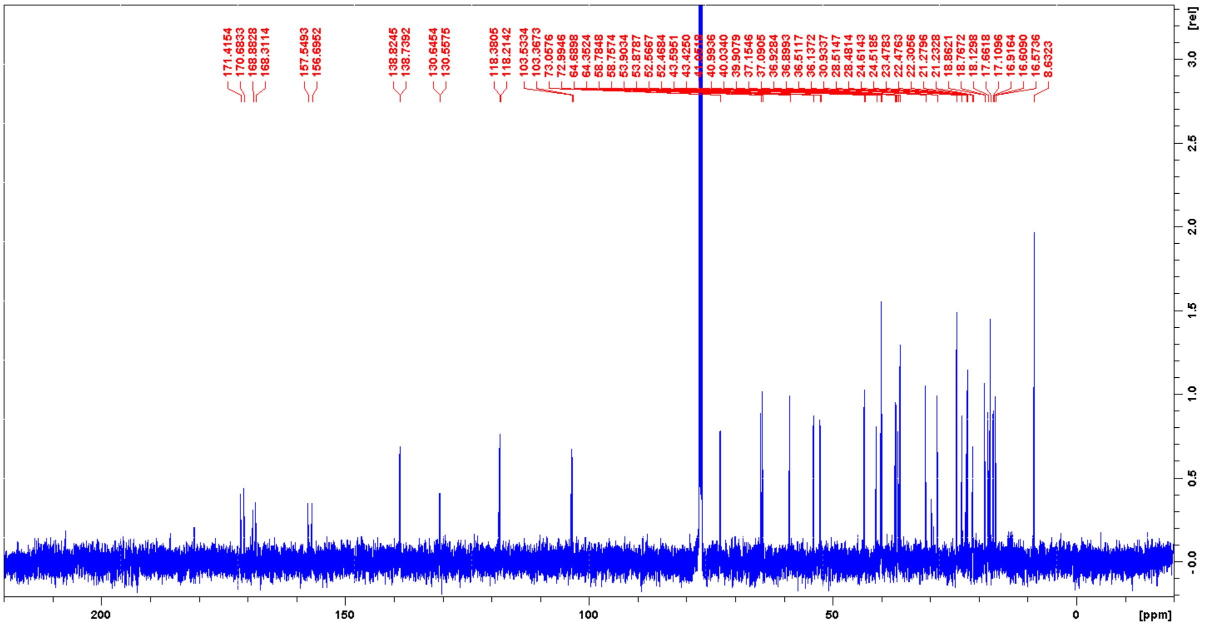

Figure S-93. ^{13}C NMR spectrum of compound **14** ($CDCl_3$, 150 MHz).

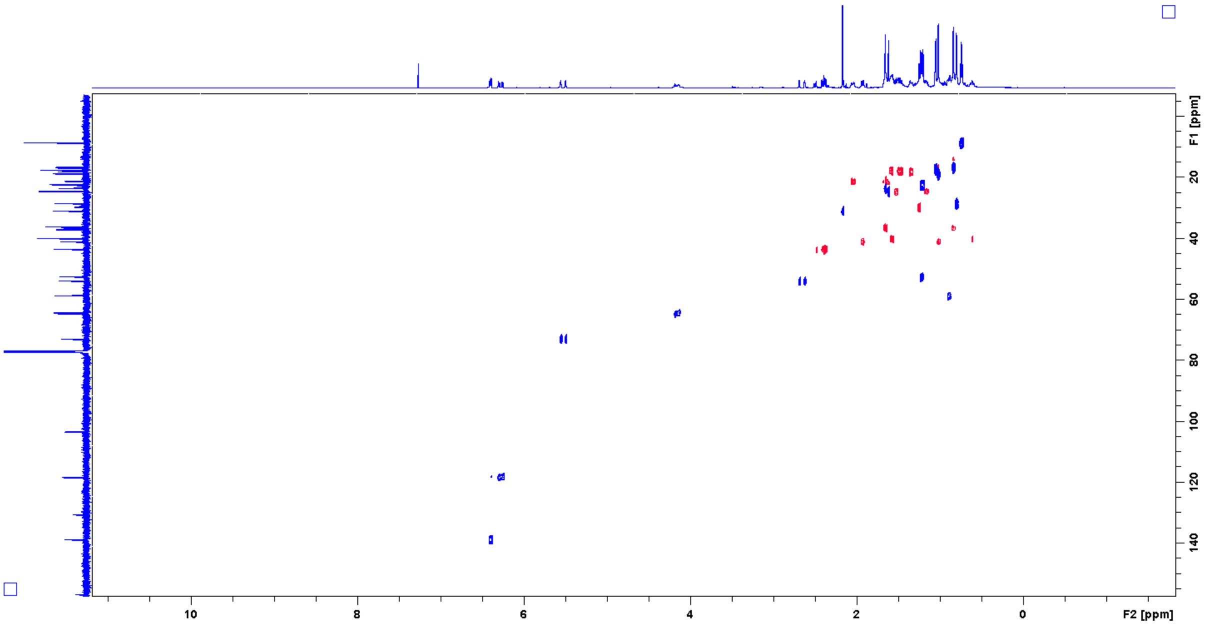

Figure S-94. HSQC spectrum of compound **14** ($CDCl_3$, 600 MHz).

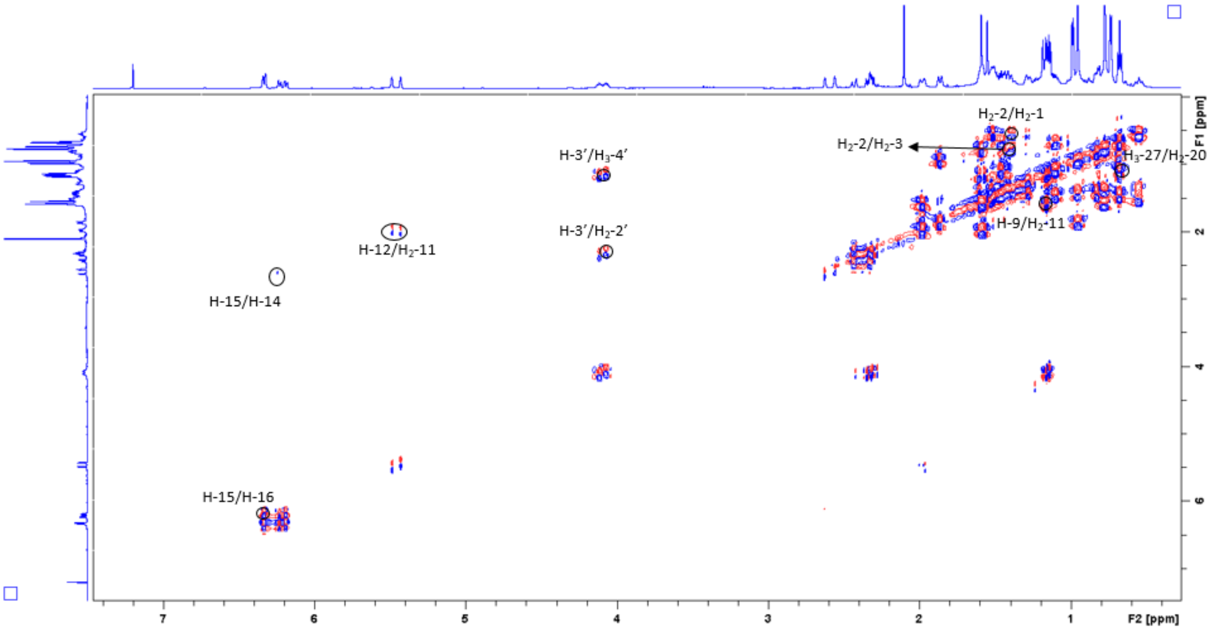

Figure S-95. ^1H-^1H COSY spectrum of compound **14** ($CDCl_3$, 600 MHz).

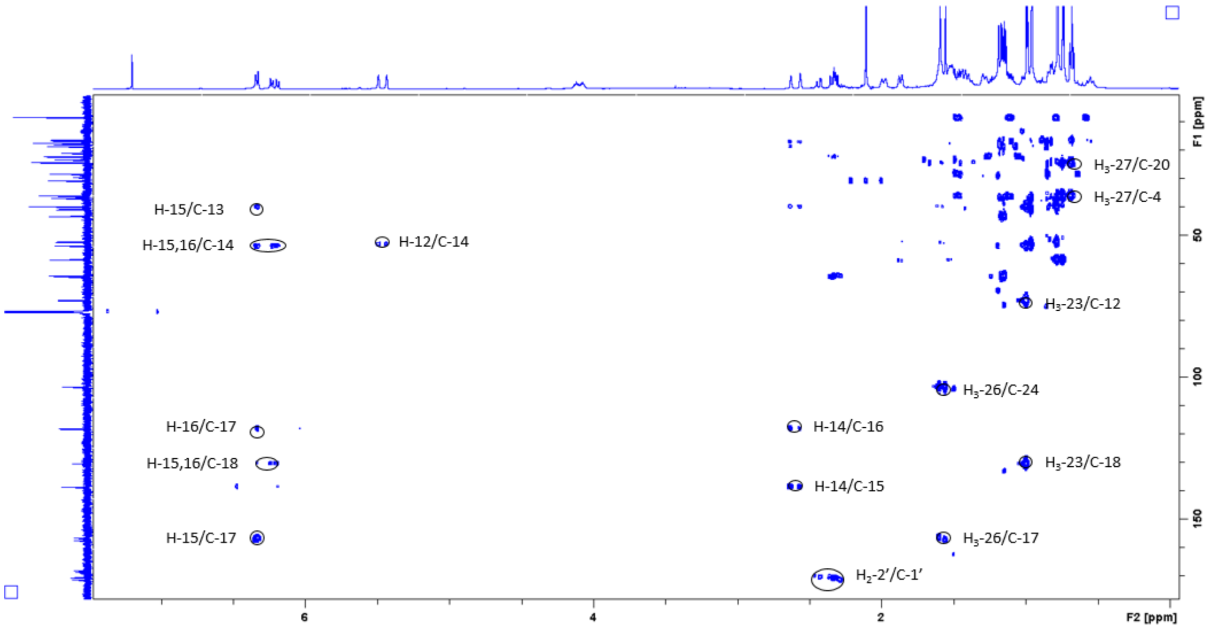

Figure S-96. HMBC spectrum of compound **14** ($CDCl_3$, 600 MHz).

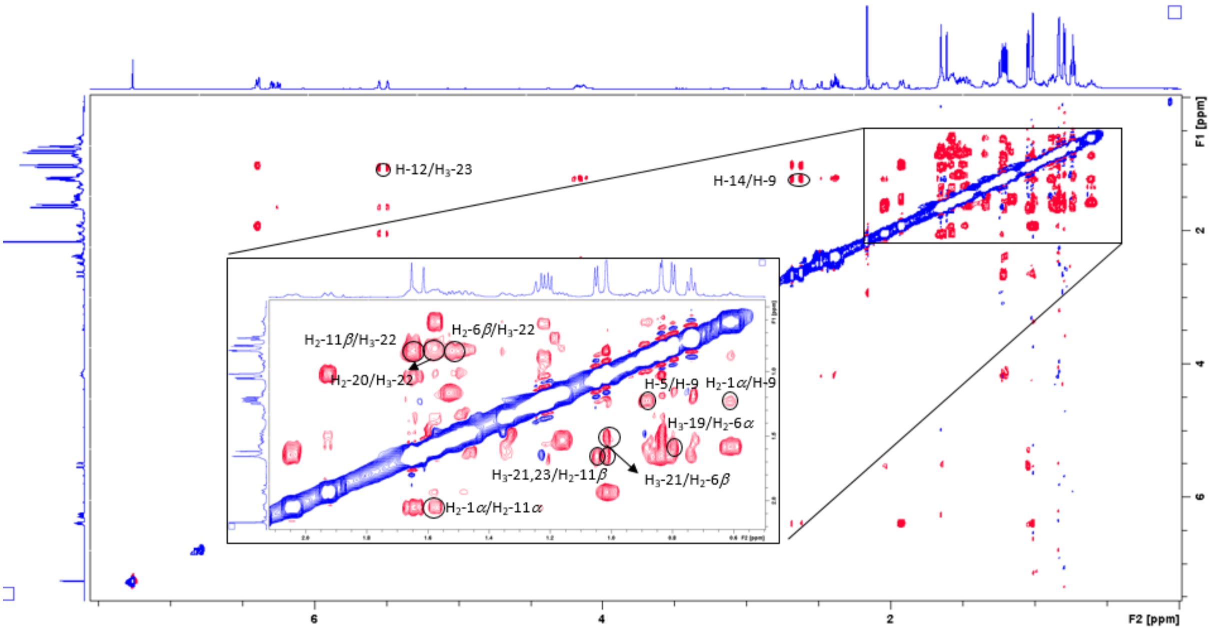

Figure S-97. NOESY spectrum of compound **14** ($CDCl_3$, 600 MHz).

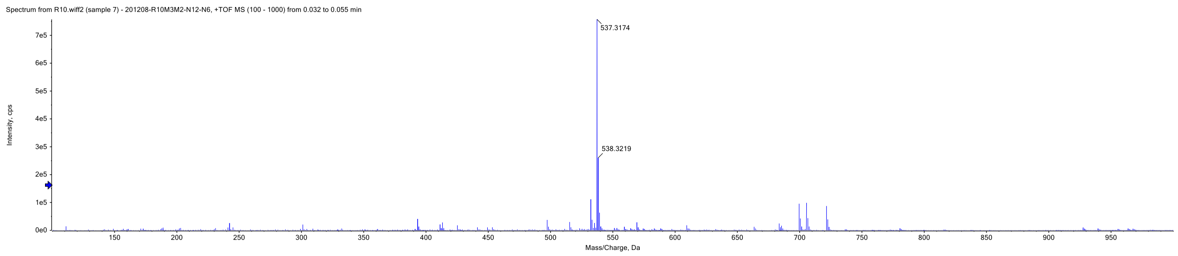

Figure S-98. HRMS spectrum of compound **14**.

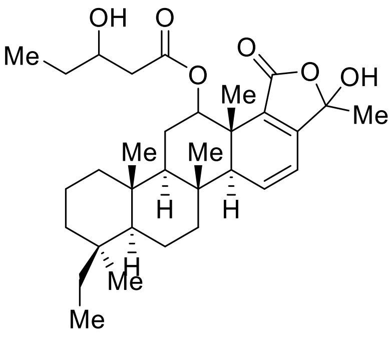

15

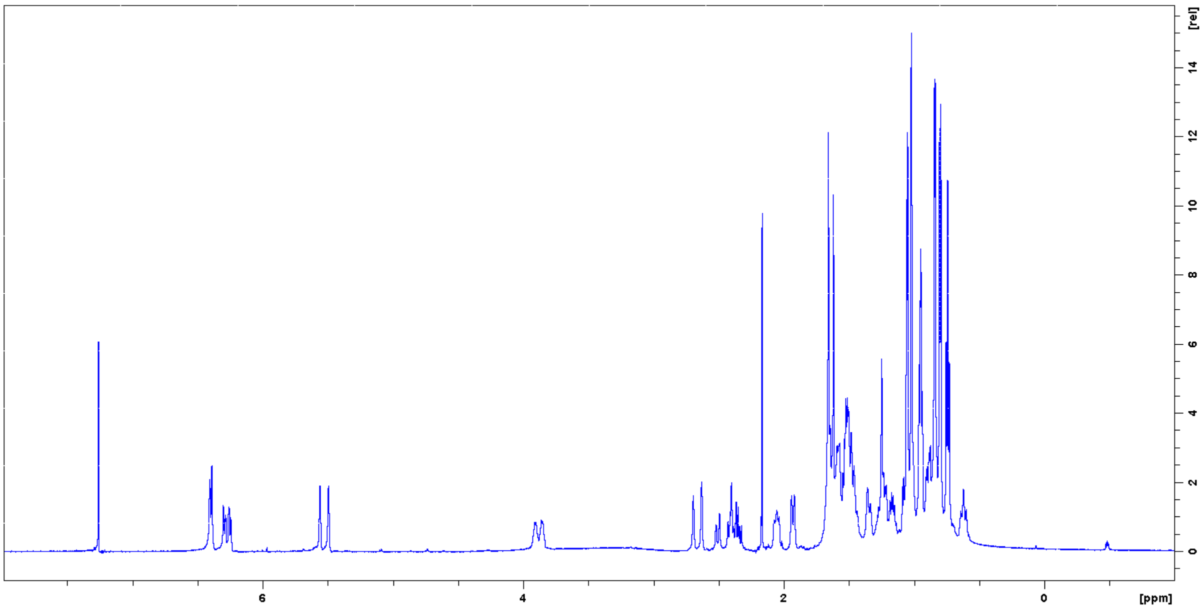

Figure S-99. ^{1}H NMR spectrum of compound **15** ($CDCl_3$, 600 MHz).

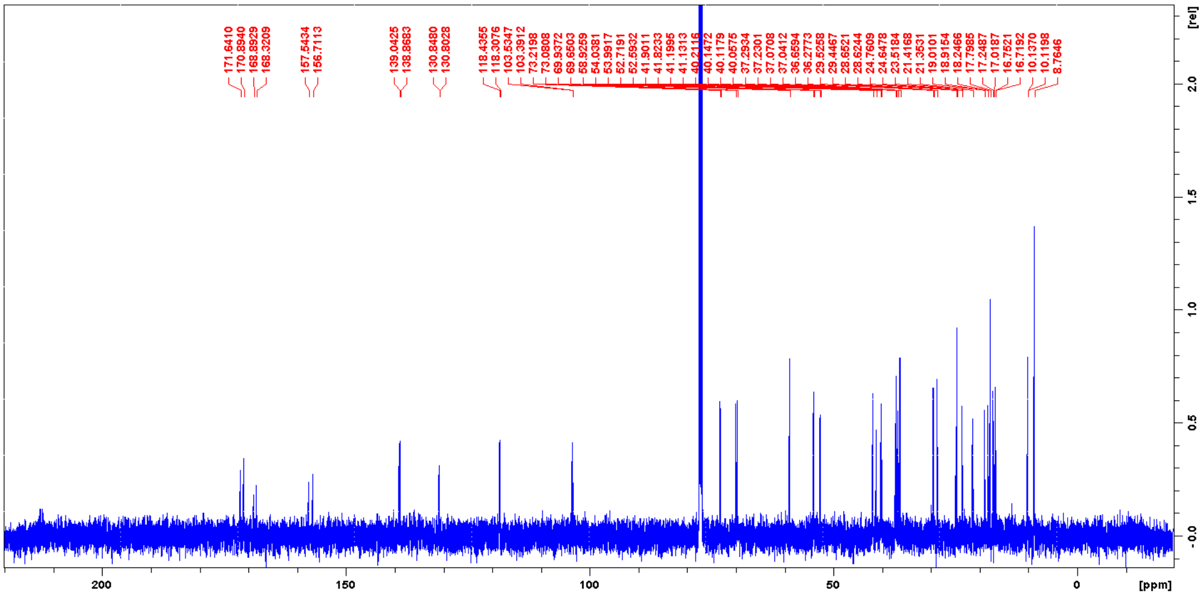

Figure S-100. ^{13}C NMR spectrum of compound **15** ($CDCl_3$, 150 MHz).

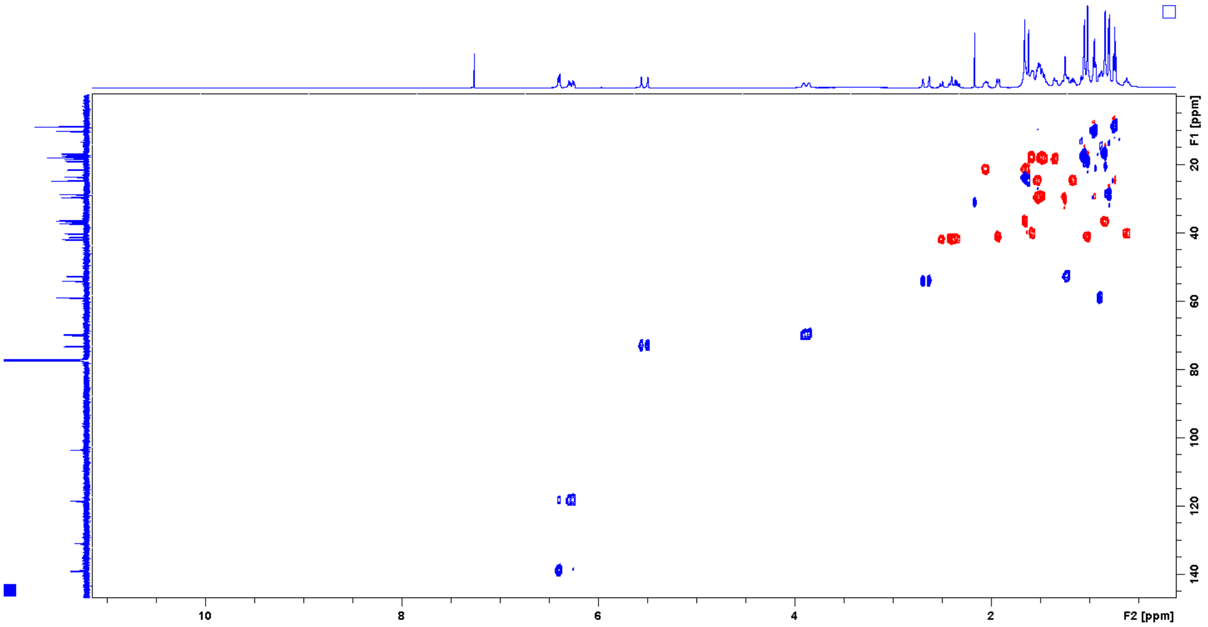

Figure S-101. HSQC spectrum of compound **15** ($CDCl_3$, 600 MHz).

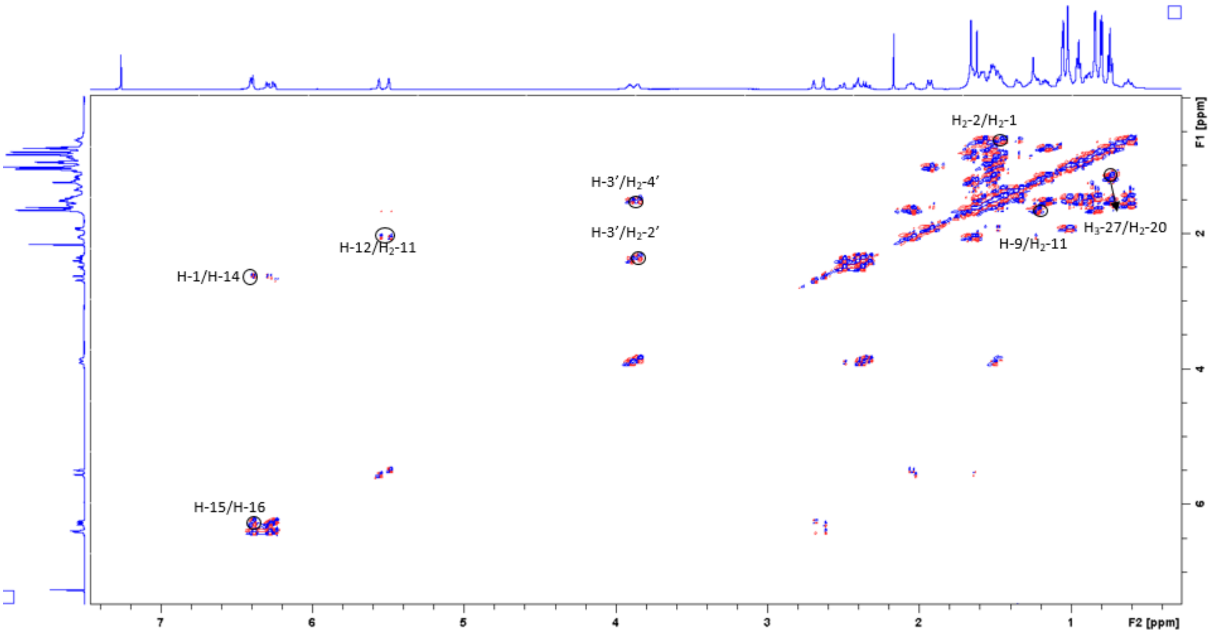

Figure S-102. ^{1}H-^{1}H COSY spectrum of compound **15** ($CDCl_3$, 600 MHz).

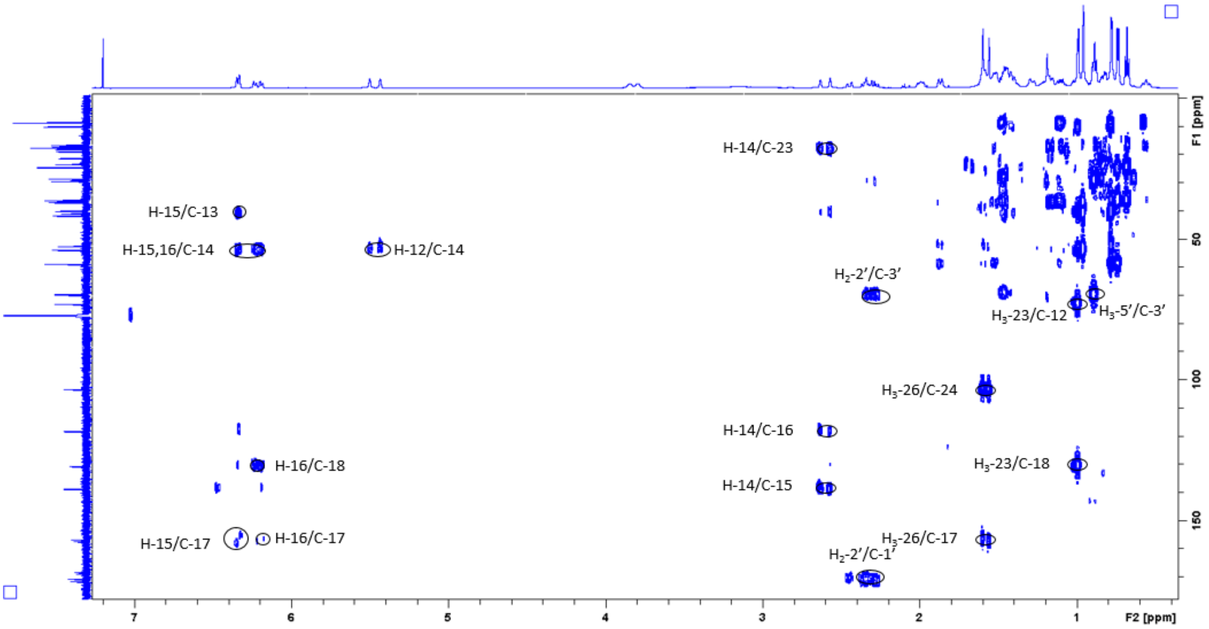

Figure S-103. HMBC spectrum of compound **15** ($CDCl_3$, 600 MHz).

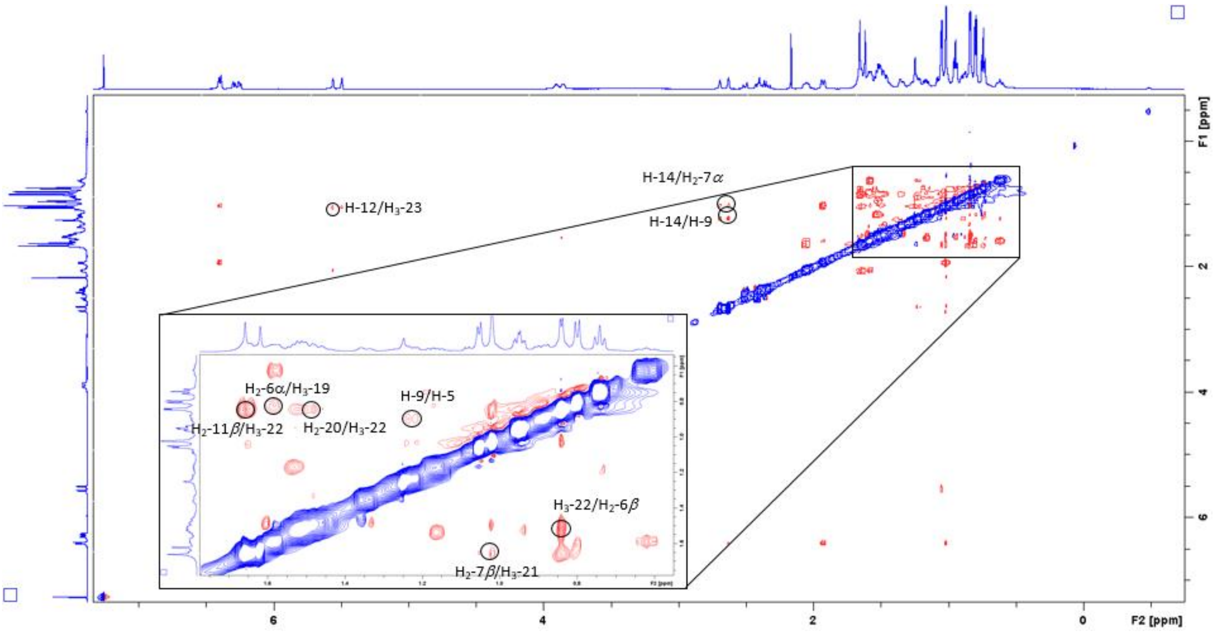

Figure S-104. NOESY spectrum of compound **15** ($CDCl_3$, 600 MHz).

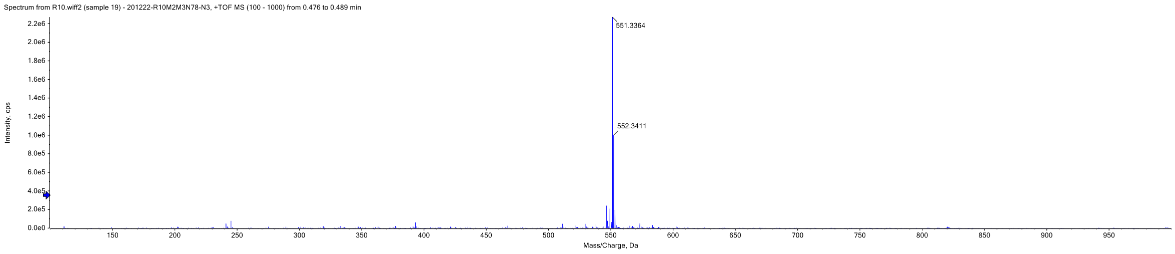

Figure S-105. HRMS spectrum of compound **15**.

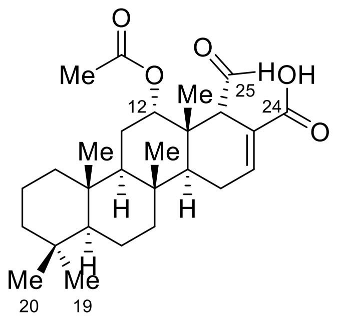

16

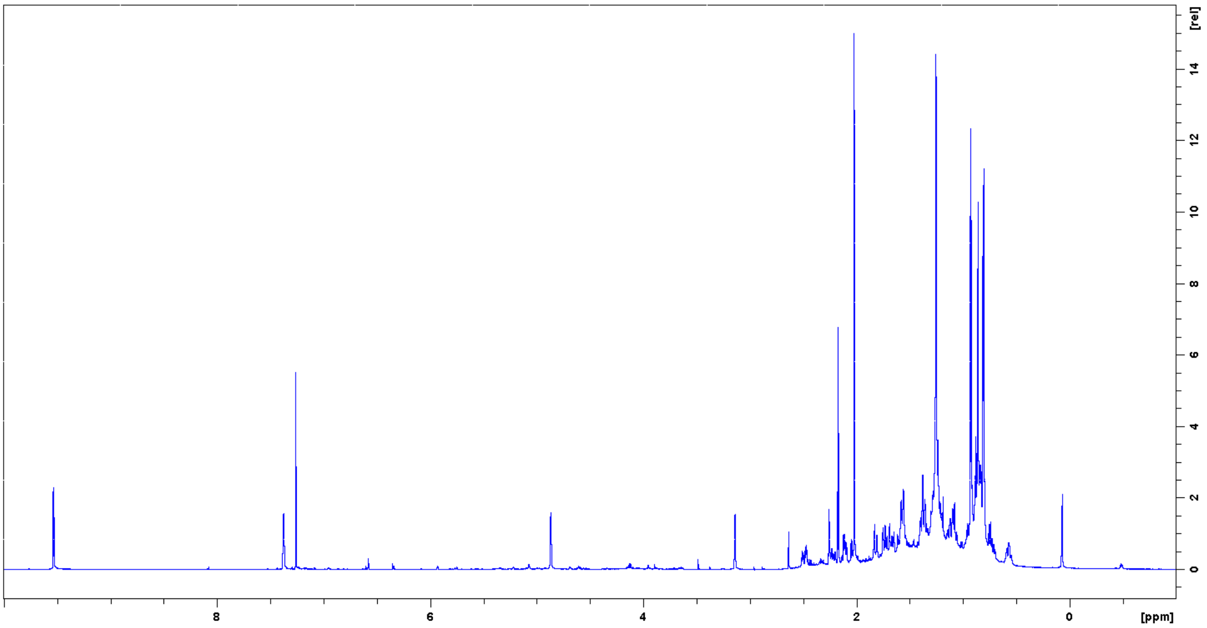

Figure S-106. ^{1}H NMR spectrum of compound **16** ($CDCl_3$, 600 MHz).

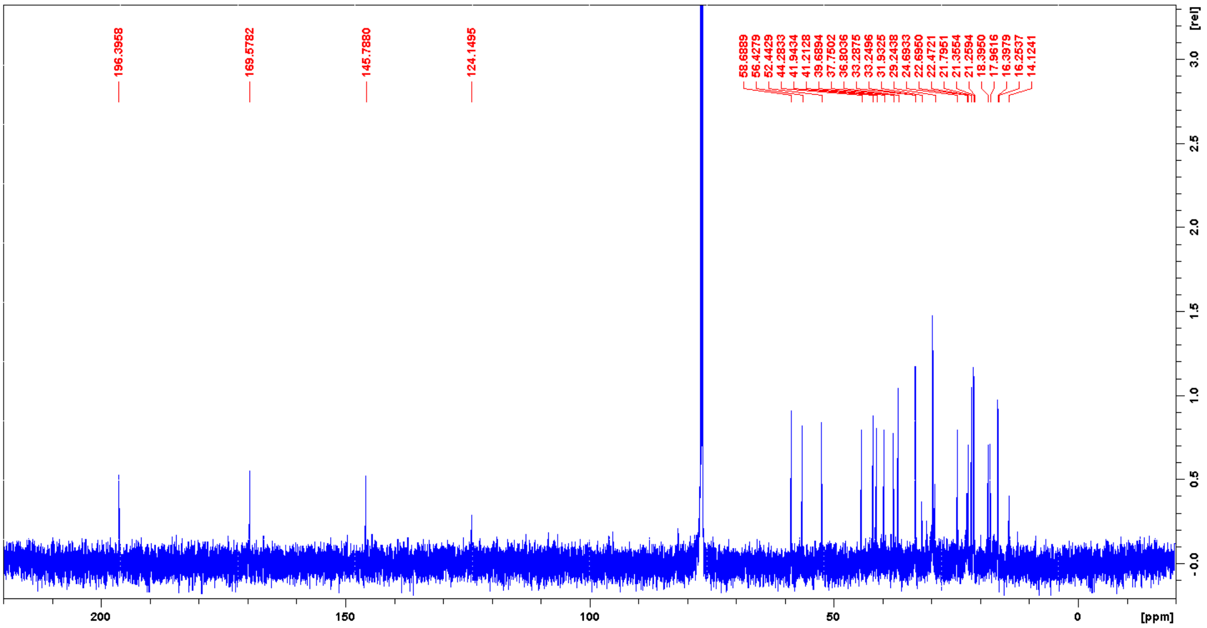

Figure S-107. ^{13}C NMR spectrum of compound **16** ($CDCl_3$, 150 MHz).

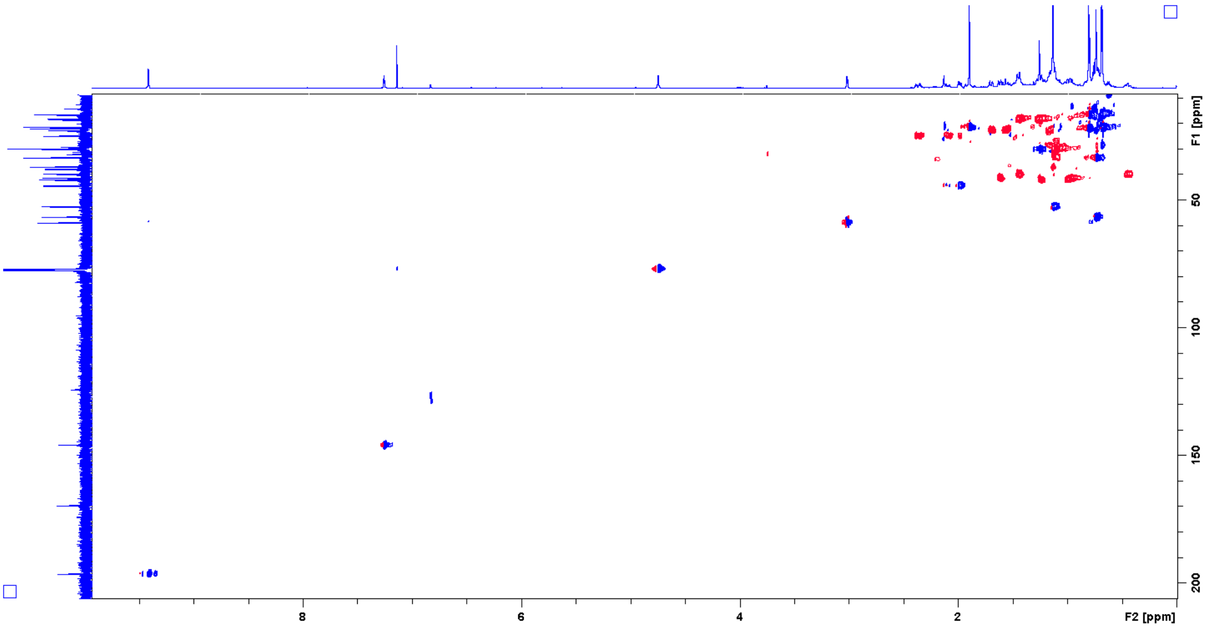

Figure S-108. HSQC spectrum of compound **16** ($CDCl_3$, 600 MHz).

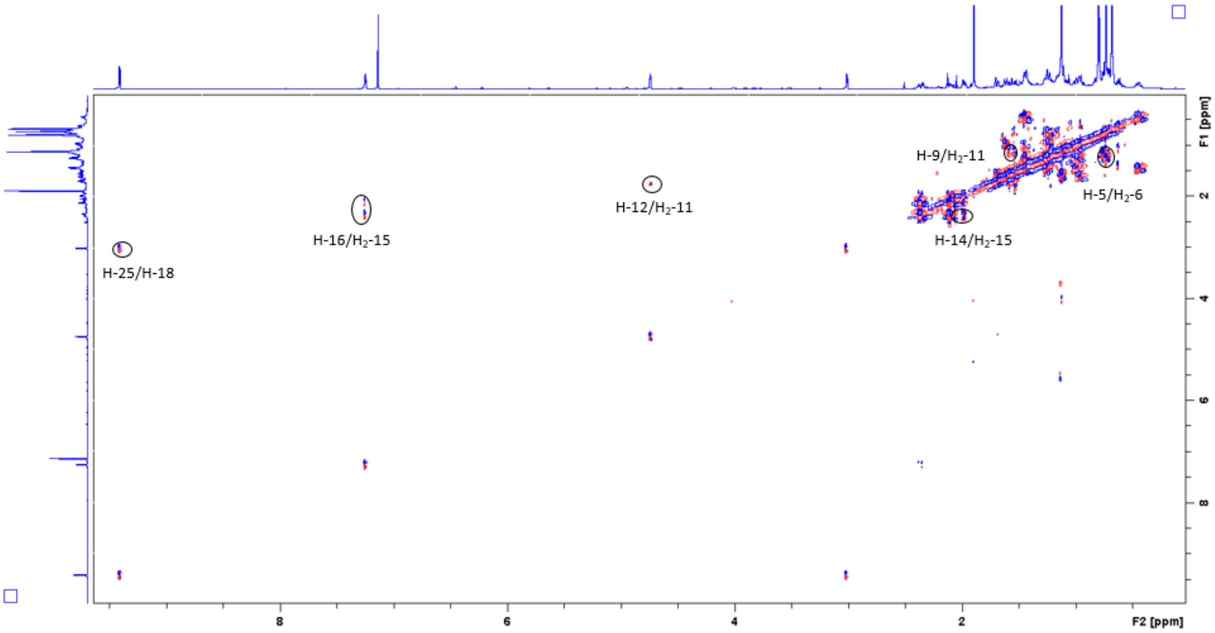

Figure S-109. ^1H-^1H COSY spectrum of compound **16** ($CDCl_3$, 600 MHz).

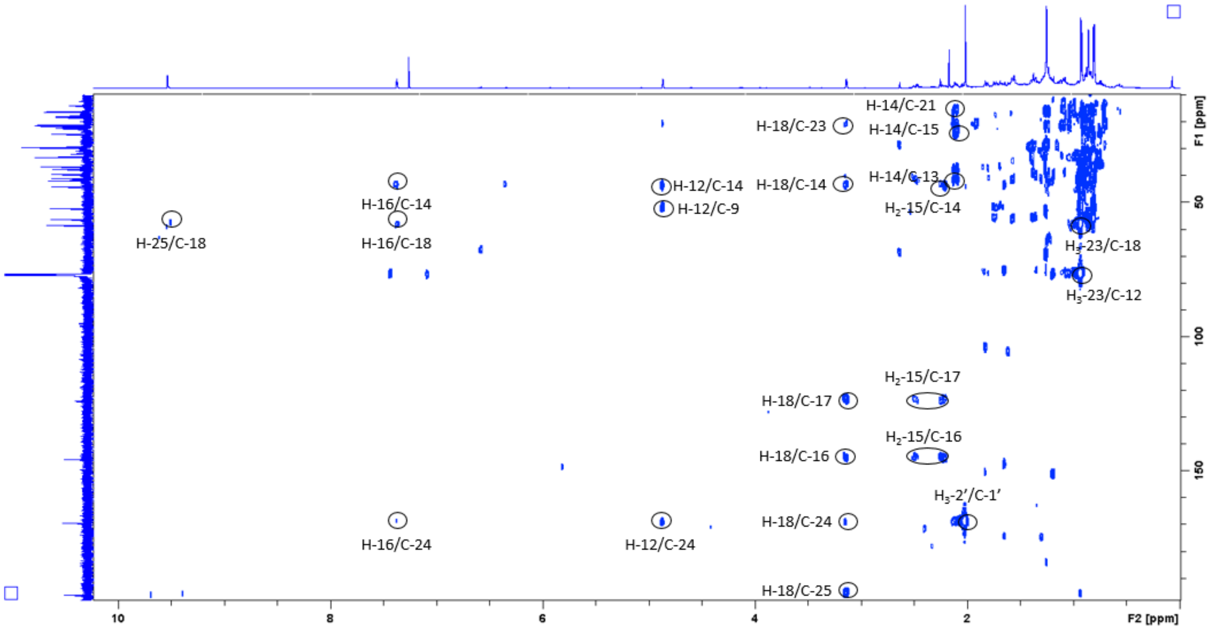

Figure S-110. HMBC spectrum of compound **16** ($CDCl_3$, 600 MHz).

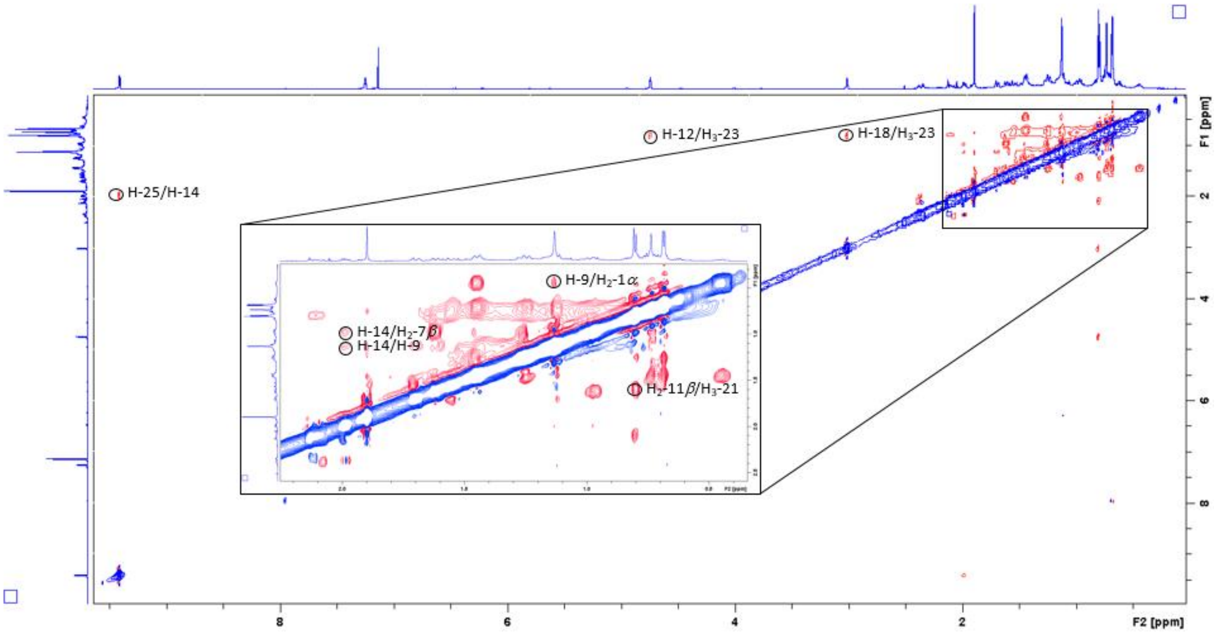

Figure S-111. NOESY spectrum of compound **16** ($CDCl_3$, 600 MHz).

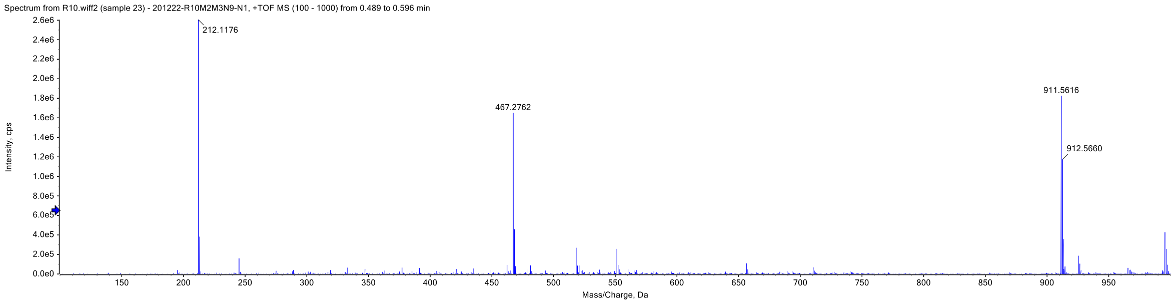

Figure S-112. HRMS spectrum of compound **16**.
