# Supplementary material for: Isolation of Scalarane-Type Sesterterpenoids from the Marine Sponge Dysidea sp. and Stereochemical Reassignment of 12-epi-Phyllactone D/E
Source: Mar Drugs. 2021 Nov 9;19(11):627. doi: 10.3390/md19110627 (PMC8624410; doi:10.3390/md19110627)
Supplement: Supplementary file 1 [file marinedrugs-19-00627-s001.zip › SI-MD-revision.pdf]

Supporting Information

Isolation of Scalarane-type Sesterterpenoids from the Marine Sponge *Dysidea* sp. and Stereochemical Reassignment of 12-*epi*-phyllactone D/E

A-Young Shin,[1,2] *Arang Son,*[3] *Changhoon Choi,*[3]

*and Jihoon Lee**[1,2]

[1]Korea Institute of Ocean Science and Technology (KIOST), Busan 49111, Republic of Korea

[2]Department of Marine Biotechnology, University of Science and Technology (UST), Daejeon 34113, Republic of Korea

[3]Department of Radiation Oncology, Samsung Medical Center, Seoul 06351, Republic of Korea

Table of contents

I. Experimental procedure 3

1. General information 3

Figure S1. Structures of known compounds isolated from *Dysidea* sp. 3

Table S1. ^{13}C (150 MHz) and ^{1}H (600 MHz) NMR data for compound **1**. 4

Table S2. ^{13}C (150 MHz) and ^{1}H (600 MHz) NMR data for compound **2**. 5

Table S3. ^{13}C (150 MHz) and ^{1}H (600 MHz) NMR data for compound **3**. 6

Table S4. ^{13}C (150 MHz) and ^{1}H (600 MHz) NMR data for compound **4**. 7

Table S5. ^{13}C (150 MHz) and ^{1}H (600 MHz) NMR data for compound **5**. 8

Table S6. ^{13}C (150 MHz) and ^{1}H (600 MHz) NMR data for compound **6**. 9

Table S7. ^{13}C (150 MHz) and ^{1}H (600 MHz) NMR data for compound **7**. 10

Table S8. ^{13}C (150 MHz) and ^{1}H (600 MHz) NMR data for compound **8**. 11

Table S9. ^{13}C (150 MHz) and ^{1}H (600 MHz) NMR data for compound **9**. 12

Table S10. ^{13}C (150 MHz) and ^{1}H (600 MHz) NMR data for compound **10**. 13

Table S11. ^{13}C (150 MHz) and ^{1}H (600 MHz) NMR data for compound **11**. 14

Table S12. ^{13}C (150 MHz) and ^{1}H (600 MHz) NMR data for compound **12**. 15

Table S13. ^{13}C (150 MHz) and ^{1}H (600 MHz) NMR data for compound **13**. 16

Table S14. ^{13}C (150 MHz) and ^{1}H (600 MHz) NMR data for compound **14**. 17

Table S15. ^{13}C (150 MHz) and ^{1}H (600 MHz) NMR data for compound **15**. 18

Table S16. ^{13}C (150 MHz) and ^{1}H (600 MHz) NMR data for compound **16**. 19

Figure S2. Key ^{1}H-^{1}H COSY and HMBC correlations of compounds **1-16**........ 20

Figure S3. Key NOESY correlations of compounds **1-16**. 21

Table S17. ^{13}C chemical shifts of phyllactone D/E (**17**, **18**), 12-*epi*-phyllactone D/E (**15**) analyzed by Li et al[1], Andersen et al[2], and our experimental data. 23

Table S18. ^{1}H chemical shifts of phyllactone D/E (**17**, **18**), 12-*epi*-phyllactone D/E (**15**) analyzed by Li et al[1], Andersen et al[2], and our experimental data. 24

Table S19. ^{13}C chemical shifts of representative examples of 12α-epimers (**3**, **4**, **12**, **14**, **15**) and 12β-epimers (**17**, **18**, **20**, **21**[1]). 25

Figure S4. Comparison of $|\Delta_C|$ values at C-9, 11, 12, 14, 23 for 12α-epimers (**3**, **4**, **12**, **14**, **15**) and 12β-epimers (**17**, **18**, **20**, **21**) relative to those reported by Li et al. 26

Figure S5. Comparison of $|\Delta_C|$ values at C-9, 11, 12, 14, 23 for 12α-epimers (**3**, **4**, **12**, **14**) and 12β-epimers (**17**, **18**, **20**, **21**, Li et al) relative to our experimental data for **15**. 26

II. Computational methods 27

1. Geometry optimization 27

I. Experimental procedure

1. General information

Specific optical rotations were obtained on a Rudolph Research Analytical (Autopol III) polarimeter. IR spectra were recorded on a JASCO FT/IR-4100 spectrophotometer. The 1D (^{1}H and ^{13}C) and 2D (COSY, HSQC, HMBC, and NOESY) NMR spectra were taken in $CDCl_3$ at Bruker 600 MHz spectrometer. High resolution mass-spectra were obtained on a Sciex X500R Q-TOF spectrometer (Framingham, MA, USA). equipped with an ESI source. MPLC was performed using the TELEDYNE ISCO CombiFlash Companion with the TELEDYNE ISCO RediSep Normal-phase Silica Flash Column. HPLC was performed on a PrimeLine Binary pump utilizing Silica columns (YMC-Pack Silica, 250 × 10 mm I.D., or 250 × 4.6 mm I.D., 5 μm), the Shodex RI-101, or the UV-M201.

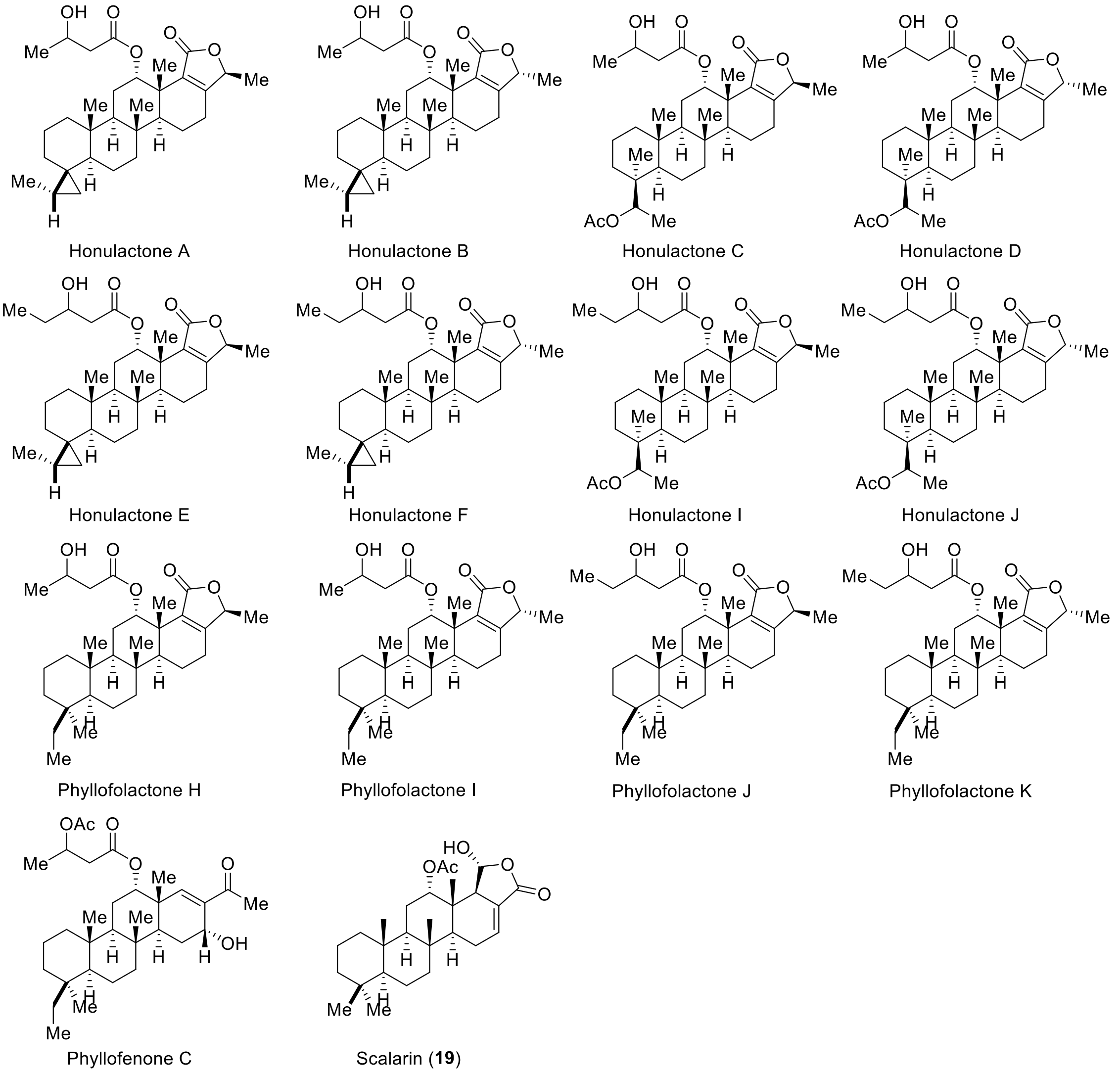

Figure S1. Structures of known compounds isolated from *Dysidea* sp.

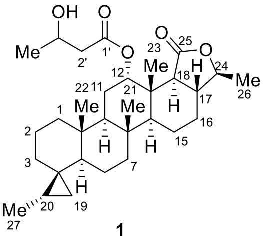

1

Table S1. ^{13}C (150 MHz) and ^{1}H (600 MHz) NMR data for compound **1**.

Position	δ_C	δ_H (*J*, Hz)	^{1}H-^{1}H COSY	HMBC	NOESY
1	39.9	1.70, m; 0.72, m	2		9
2	21.2	1.44, m; 1.44, m	1		
3	33.2	1.53, td (12.6, 5.0); 1.24, m			3β(1.24)-27
4	22.7				
5	50.3	1.39, m	6		7α(0.93)
6	17.7	1.01, m; 1.01, m	5		
7	40.4	1.69, m; 0.93, m			7α–5, 15α(1.76)
8	37.6				
9	51.4	1.19, m	11		
10	38.0				
11	21.5	2.03, ddd (15.0, 3.7, 1.5); 1.71, m	9, 12		11β(1.71)–21, 22
12	74.8	5.41, dd (3.0, 2.4)	11	13, 14, 1’	23
13	38.7				
14	54.3	1.23, m			18
15	20.1	1.76, m; 1.29, m	16		15β(1.29)–23
16	27.7	1.95, m; 1.15, m	15, 17		
17	44.9	1.86, dddd (13.6, 11.8, 9.7, 3.7)	16, 18, 24		
18	52.5	2.34, d (13.8)	17	13, 17, 25	14, 24
19	13.7	0.57, m; -0.49, dd (5.8, 4.3)	20	3, 5, 20, 27	19$_{cis}$ (-0.49)–27; 19$_{trans}$ (0.57)–6
20	13.5	0.72, m	19, 27		
21	16.5	0.82, s		7, 8, 9, 14	23, 27
22	14.1	0.77, s		1, 5, 9, 10	11β, 27
23	17.2	1.04, s		12, 13, 14, 18	12, 15β(1.29), 17, 21
24	80.4	4.03, dq (9.6, 6.1)	17, 26		
25	174.8				
26	18.4	1.37, d (6.0)		17, 24	
27	13.3	1.07, d (6.3)			3β, 19$_{cis}$, 22
1'	171.7				
2'	43.3	2.49, dd (16.2, 3.2); 2.42, dd (16.1, 9.0)	3’	1’, 3’	
3'	64.5	4.19, m	2’, 4’		
4'	22.5	1.23, d	3’	2’, 3’	

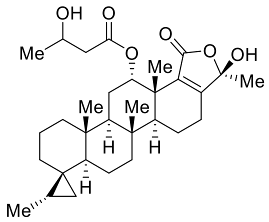

2

Table S2. ^{13}C (150 MHz) and ^{1}H (600 MHz) NMR data for compound **2**.

**Position**	δ_C	δ_H (*J*, Hz)	^{1}H-^{1}H COSY	HMBC	NOESY
1	40.0	1.70, m; 0.69, m	2		
2	21.2	1.49, m; 1.44, m	1	10	
3	33.2	1.53, m; 1.25, m			19_{cis} (-0.49)
4	22.7				
5	50.4	1.42, m			
6	17.6	1.06, m; 1.06, m			19_{trans} (0.58)
7	40.2	1.78, m; 0.99, m		14	
8	37.4				
9	52.0	1.22, m	11		14
10	38.0				
11	21.5	2.05, m; 1.73, m	9, 12		
12	75.0	5.70, dd (3.6, 2.2)	11	14	23
13	38.9				
14	52.0	1.49, m		16	9
15	16.6	1.92, m; 1.49, m	16		15α (1.92)–16α (2.28)
16	23.4	2.35, m; 2.28, m	15		16α–15α, 26
17	162.9				
18	133.7				
19	13.7	0.58, ddd (8.9, 4.4, 2.0); -0.49, dd (5.9, 4.3)	20	3, 5, 27	3, 6, 27
20	13.5	0.69, m	19, 27		22
21	17.3	0.90, s		7, 8, 9, 14	22, 23
22	14.0	0.80, s		1, 9, 10	20, 21, 27
23	20.7	1.22, s		12, 13, 14, 18	12, 21
24	104.4				
25	168.9				
26	22.7	1.56, s		17, 24	
27	13.3	1.09, d (6.3)	20	4, 20	19, 22
1'	169.1				
2'	44.1	2.56, dd (16.2, 3.0); 2.39, m	3’	1’, 3’	
3'	65.3	4.12, m	2’, 4’	1’	
4'	22.7	1.23, d (6.4)	3’	2’, 3’	

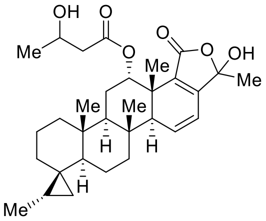

3

Table S3. ^{13}C (150 MHz) and ^{1}H (600 MHz) NMR data for compound **3**.

Position	δ_C	δ_H (*J*, Hz)	^{1}H-^{1}H COSY	HMBC	NOESY
1	39.8	1.68, m; 0.72, m			
2	21.2	1.44, m; 1.44, m			
3	33.1	1.53, m; 1.24, m			3β(1.53)–27
4	22.8/22.7				
5	50.4/50.3	1.43, m	6		9
6	17.3	1.08, m; 1.01, m	5		
7	39.5/39.4	1.86, m; 1.00, m		5	7α(1.00)–14
8	36.9				
9	50.7/50.6	1.26, m	11		5, 14
10	37.9/37.8				
11	21.5	2.12, m; 1.66, m	9, 12		11β(1.67)–23
12	73.2/73.1	5.55/5.50, dd (3.0, 2.4)	11	1’	23
13	40.2/40.1				
14	54.1/54.0	2.69/2.62, t (3.0)	15	8, 13, 15, 16, 18	7α, 9
15	138.8	6.382, dt (10.0, 2.0)/ 6.379, dt (10.0, 2.0)	14, 16	17	
16	118.6/118.4	6.29, dd (24.9, 3.2)/ 6.25, dd (24.9, 3.2)	15	17, 18, 24	
17	157.7/156.8				
18	130.8/130.7				
19	13.7	0.59, dd (8.8, 4.4); -0.48, t (5.2)	20	3, 4, 5	27
20	13.5	0.72, m	19, 27		
21	18.8/18.7	1.01, s		7, 8, 14	22
22	13.9	0.795/0.792, s		1, 5, 10	21, 27
23	17.3/17.1	1.06, s		12, 13, 14, 18	11β, 12
24	103.7/103.5				
25	169.0/168.4				
26	24.7/23.6	1.65/1.62, s		17, 24	
27	13.3	1.09/1.08, d (6.2)	20	4, 20	3β, 19, 22
1'	171.6/170.8				
2'	43.7/43.5	2.49, dd (16.2, 3.2); 2.37, m	3’	1’, 3’	
3'	64.8/64.5	4.18/4.14, m	2’, 4’		
4'	22.6/22.4	1.22/1.20 d (6.3)	3’	2’, 3’	

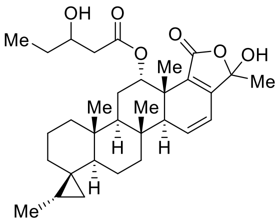

4

Table S4. ^{13}C (150 MHz) and ^{1}H (600 MHz) NMR data for compound **4**.

Position	δ_C	δ_H (*J*, Hz)	^{1}H-^{1}H COSY	HMBC	NOESY
1	39.8	1.69, m; 0.73, m		5	
2	21.2	1.45, m; 1.45, m			20, 27
3	33.1	1.24, m; 1.24, m			
4	22.7				
5	50.4/50.3	1.43, m	6		9
6	17.3	1.04, m; 1.04, m	5, 7		
7	39.5/39.4	1.86, m; 1.00, m	6	5, 9	7α (1.00)–14
8	36.9				
9	50.7/50.6	1.28/1.25, m			5, 14
10	37.9				
11	21.5	2.13, m; 1.67, m	9, 12	10, 12	11β (1.67)–21, 22, 23
12	73.2/73.1	5.56/5.50, dd (3.0, 2.4)	11	14, 1’	23
13	40.2/40.1				
14	54.1/54.0	2.69/2.62, t (3.0)	15	15, 16	7α, 9
15	138.9/138.8	6.39, dt (10.0, 2.0)/ 6.38, dt (10.0, 2.0)	14, 16	14, 17	
16	118.5/118.4	6.28, dd (24.8, 3.1)/ 6.25, dd (24.8, 3.1)		14, 17, 18	
17	157.6/156.7				
18	130.9/130.8				
19	13.7	0.52, dd (8.8, 4.4); -0.54, t (5.1)	20	3, 5	3
20	13.5	0.70, m	19, 27		2
21	18.8/18.7	1.01, s		7, 8, 9, 14	11β, 22
22	13.9	0.799/0.792, s		5	11β, 21, 27
23	17.3/17.1	1.008/1.002, s		12, 13, 14, 18	11β, 12
24	103.6/103.4				
25	169.0/168.4				
26	24.7/23.5	1.66/1.63, s		17, 24	
27	13.5/13.3	1.08/1.07, d (6.1)	20	4	2, 22
1'	171.7/170.9				
2'	41.9/41.8	2.51, dd (16.0, 3.0); 2.35, m	3’	1’, 3’	
3'	69.9/69.6	3.90/3.86, m	2’, 4’		
4'	29.5/29.4	1.50, m	3’, 5’	3’	
5’	10.1	0.95, t (7.4)	4’	3’	

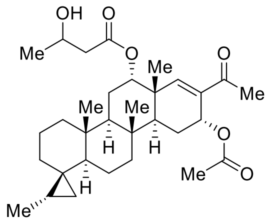

5

Table S5. ^{13}C (150 MHz) and ^{1}H (600 MHz) NMR data for compound **5**.

Position	δ_C	δ_H (J, Hz)	^{1}H-^{1}H COSY	HMBC	NOESY
1	39.5	1.67, m; 0.89, m			
2	21.3	1.46, m; 1.46, m			
3	33.2	1.53, m; 1.25, m			
4	22.7				
5	50.3	1.47, m	6	10	7α (0.79)
6	17.5	1.05 ,m; 1.05 ,m			19$_{trans}$ (0.59)
7	39.9	1.67, m; 0.97, m			7α–5, 9; 7β (1.67)–15α (1.89)
8	36.6				
9	51.8	1.36, dd (13.2, 2.5)	11		7α, 14
10	38.0				
11	22.5	1.89, m; 1.78, m	9, 12	10, 13	11β (1.78)–23
12	76.8	5.11, dd (3.0, 2.4)	11	9, 14	23
13	41.4				
14	44.4	1.76 , m			9
15	24.1	1.89, m; 1.61, m	16	13, 17	15α (1.89)–7β; 15β (1.61)–21
16	65.3	5.76, dd (4.3, 1.6)	15	14, 17, 18, 16-CH$_3$<u>C</u>O	
17	135.1				
18	153.2	6.72, s		12, 14, 16, 17, 24	
19	13.7	0.59, dd (8.8, 4.4); -0.49, t (5.1)	20	3, 5, 20	19$_{cis}$ (-0.49)–27; 19$_{trans}$–6
20	13.5	0.70, m	19, 27	5	
21	17.1	0.84, s		8, 9	15β, 22, 23
22	14.0	0.80, s		1, 5, 10	21, 27
23	19.9	1.06, s		12, 13, 14, 18	11β, 12, 21
24	197.7				
25					
26	25.9	2.22, s		17, 24	
27	13.3	1.08, d (6.3)	20	4, 19	19$_{cis}$, 22
16-CH$_3$<u>C</u>O	170.2				
16-<u>C</u>H$_3$CO	21.3	2.02, s		16-CH$_3$<u>C</u>O	
1'	172.0				
2'	43.9	2.53, dd (16.0, 3.4); 2.39, m	3’	1’, 3’	
3'	64.6	4.19, m	2’, 4’	1’	
4'	22.9	1.25, d	3’	2’, 3’	

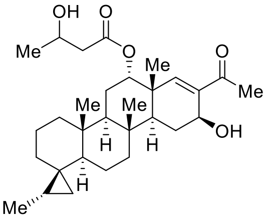

6

Table S6. ^{13}C (150 MHz) and ^{1}H (600 MHz) NMR data for compound **6**.

Position	δ_C	δ_H (*J*, Hz)	^1H-^1H COSY	HMBC	NOESY
1	39.9	1.68, m; 0.77, m	2	3, 5	
2	21.3	1.47, m; 1.47, m	1		
3	33.2	1.55, m; 1.26, m			3β(1.26)–19_{cis} (-0.49)
4	22.7				
5	50.4	1.44, m		4, 10, 22	7α(0.95)
6	17.6	1.08, m; 1.08, m		5, 7	19_{trans} (0.61), 20
7	39.6	1.79, m; 0.95, m			7α–5; 7β(1.79)–15α (2.14)
8	37.0				
9	51.5	1.28, d (6.3)			14
10	38.0				
11	22.4	1.87, m; 1.78, m	12	12, 13	11β(1.87)–21
12	77.1	5.09, dd (3.0, 2.4)	11	1’	23
13	41.6				
14	47.6	1.49, m		18	9
15	25.7	2.14, m; 1.48, m	16	13, 14, 16, 17	15α–7β; 15β(1.48)–21
16	68.1	4.62, dd (9.6, 5.1)	15	15	
17	139.1				
18	152.0	6.62, s		14, 16, 17, 23	
19	13.8	0.61, dd (8.8, 4.4); -0.47, dd (5.9, 4.4)	20	3, 4, 5, 20, 27	6, 19_{cis}–3β, 27
20	13.5	0.73, m	19, 27		6
21	17.0	0.91, s		8, 9, 14	11β, 15β, 22, 23
22	14.0	0.81, s		1, 5	21
23	21.3	1.21, s		12, 18	12, 21
24	202.2				
25					
26	25.8	2.28, s		17, 18, 24	
27	13.3	1.11, d (6.2)	20		19_{cis}
1'	172.3				
2'	43.5	2.49, dd (16.2, 3.7); 2.44, dd (16.1, 8.6)	3’	1’, 3’	
3'	64.6	4.15, m	2’, 4’	1’	
4'	22.8	1.26, d	3’	2’, 3’	

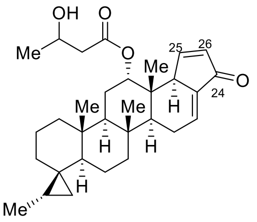

7

Table S7. ^{13}C (150 MHz) and ^{1}H (600 MHz) NMR data for compound **7**.

**Position**	δ_C	δ_H (*J*, Hz)	**^{1}H-^{1}H COSY**	**HMBC**	**NOESY**
1	39.8	1.70, m; 0.80, m	2		
2	21.1	1.47, m; 1.47, m	1		
3	33.1	1.56, m; 1.26, m			3β(1.25)–27
4	22.6				
5	50.1	1.46, m			9
6	17.4	1.03, m; 1.03, m			
7	40.0	1.66, m; 1.04, m		5	7α(0.96)–9
8	37.8				
9	51.2	1.39, m	11		5, 7α, 14
10	37.6				
11	22.9	1.86, m; 1.77, m	9, 12		11β(1.78)–21, 23
12	74.7	5.20, dd (3.0, 2.8)	11	1’	23
13	38.6				
14	50.3	1.77, m	15		9, 18
15	24.5	2.42, m; 2.11, m	14, 16		15β(2.11)–21
16	130.4	6.64, d (4.3)	15		
17	136.4				
18	49.3	3.35, d (2.6)		13, 17, 23, 25	14
19	13.6	0.59, m;<br>-0.49, t (5.2)	20	3, 20	19_{trans} (0.57)–27
20	13.4	0.70, m	19, 27		22
21	15.8	0.89, s		7	11β, 15β, 23
22	14.2	0.81, s		1, 9	20
23	14.5	0.61, s		12, 13	11β, 12, 21
24	195.9				
25	157.5	7.38, dd (6.4, 2.0)	18, 26	17, 18, 24	
26	137.4	6.34, dd (6.1, 2.4)	25	17, 24, 25	
27	13.2	1.08, d (6.2)	20	4, 19, 20	3β, 19_{trans}
1'	172.1				
2'	43.3	2.57, dd (16.3, 3.5);<br>2.50, dd (16.3, 8.8)	3’	3’	
3'	64.5	4.23, m	2’, 4’		
4'	22.6	1.27, d (6.3)	3’	2’	

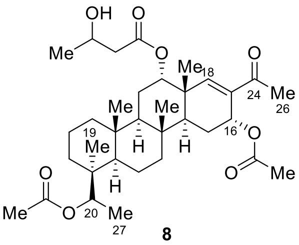

8

Table S8. ^{13}C (150 MHz) and ^{1}H (600 MHz) NMR data for compound **8**.

Position	δ_C	δ_H (*J*, Hz)	^1H-^1H COSY	HMBC	NOESY
1	40.5	1.64, m; 0.74, td (12.5, 4.0)	2	10	1α(0.74)–9; 1β(1.64)–22
2	18.0	1.47, m; 1.39, m	1, 3		2β(1.47)–20, 22
3	39.1	1.67, m; 1.02, m	2		3β(1.67)–27
4	39.4				
5	58.9	1.03, m	6		9
6	19.9	1.75, m; 1.41, m	5, 7		
7	41.8	1.70, m; 0.79, m	6		
8	36.9				
9	54.1	1.29, m	11		1α, 5, 14
10	37.3				
11	22.4	1.83-1.74, m; 2H	9, 12		
12	76.8	5.11, dd (3.0, 2.4)	11	9, 14	23
13	41.3				
14	44.1	1.72, m	15		9
15	24.1	1.86, d (14.6); 1.60, m	14, 16	13, 16	
16	65.2	5.74, dd (4.3, 1.6)	15	14, 18, 16-CH3<u>C</u>O	26
17	135.1				
18	153.2	6.70, s		12	
19	23.3	0.99, s		5, 20	
20	73.2	5.35, q (6.3)	27	20-CH3<u>C</u>O	2β, 22
21	16.9	0.82, s		8, 9, 14	
22	16.6	0.87, s		1, 5, 9	1β, 2β
23	19.9	1.05, s		12, 13, 18	12
24	197.8				
25					
26	25.9	2.21, s		17, 24	16
27	15.9	1.09, d (6.3)	20	4, 20	3β
20-CH3<u>C</u>O	170.6				
20-<u>C</u>H3CO	22.0	2.03, s		20, 20-CH3<u>C</u>O	
16-CH3<u>C</u>O	170.2				
16-<u>C</u>H3CO	21.4	2.03, s		16	
1'	172.0				
2'	43.8	2.52, dd (16.0, 3.4); 2.46, dd (16.0, 8.9)	3’	1’, 3’	
3'	64.5	4.18, m	2’, 4’		
4'	22.9	1.25, d (6.3)	3’	2’, 3’	

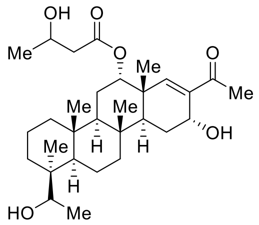

9

Table S9. ^{13}C (150 MHz) and ^{1}H (600 MHz) NMR data for compound **9**.

Position	δ_C	δ_H (*J*, Hz)	^1H-^1H COSY	HMBC	NOESY
1	40.8	1.63, m; 0.74, m	2		
2	18.0	1.46, m; 1.36, m	1, 3		2*β* (1.46)–20
3	39.5	1.60, m; 1.04, m	2		3*β* (1.60)–27
4	40.6				
5	59.3	1.02, m			9
6	22.5	1.82, m; 1.82, m			
7	41.8	1.82, m; 1.01, m			
8	37.1				
9	54.1	1.37, m	11	8, 11	5, 14
10	37.4				
11	21.0	1.81, m; 1.77, m	9, 12		11*β* (1.82)–22
12	77.1	5.11, dd (3.0, 2.4)	11	9, 14	23
13	41.6				
14	43.7	1.86, d (13.2)			9
15	25.3	1.86, m; 1.60, m	16		
16	63.3	4.55, d (3.8)	15	14, 17, 18	
17	138.4				
18	151.8	6.59, s		12, 14, 16, 24	
19	22.0	0.86, s		5, 20	
20	70.1	4.32, q (6.3)	27	19	2*β*, 22
21	16.8	0.91, s		7, 8, 9	
22	16.5	0.98, s		1, 5, 9, 10	11*β*, 20
23	19.9	1.08, s		12, 18	12
24	201.5				
25					
26	25.6	2.25, s		17, 24	
27	19.9	1.08, d (6.2)	20	4, 20	3*β*
1'	172.2				
2'	43.9	2.49, dd (16.1, 3.2); 2.41, dd (16.0, 9.2)	3’	1’, 3’	
3'	64.6	4.15, m	2’, 4’		
4'	22.9	1.22, d (6.3)	3’	2’, 3’	

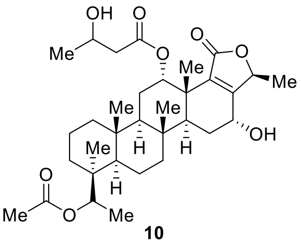

10

Table S10. ^{13}C (150 MHz) and ^{1}H (600 MHz) NMR data for compound **10**.

**Position**	δ_C	δ_H (*J*, Hz)	**^{1}H-^{1}H COSY**	**HMBC**	**NOESY**
1	40.5	1.66, m; 0.64, m	2	10	1α(0.64)–9; 1β(1.66)–22
2	17.9	1.45, m; 1.36, m	1, 3		
3	39.1	1.66, m; 1.00, m	2	4	3β(1.66)–27
4	39.4				
5	59.0	0.98, m			9
6	20.1	1.76, m; 1.42, m		5	
7	42.4	1.78, m; 0.94, m		8, 9	
8	37.2				
9	53.9	1.20, m			1α, 5, 14
10	37.2				
11	21.2	2.05, m; 1.71, m	12		11β(1.71)–22, 23
12	74.4	5.61, dd (3.0, 2.4)	11	9	23
13	39.1				
14	45.9	1.76, m			9
15	27.8	1.91, d (13.8); 1.84, td (14.2, 13.6, 4.7)	16	13, 16, 17	15β(1.84)–21, 23
16	61.6	4.44 , dd (4.7, 1.4)	15	14, 17, 18	26
17	162.1				
18	135.6				
19	23.3	0.96, s		3, 5	
20	73.3	5.35, q (6.3)	27	3, 5, 19, 20-$CH_3\underline{C}O$	22
21	16.8	0.87, s		7, 8, 9, 14	15β, 23
22	16.6	0.87, s		1, 5, 9, 10	1β, 11β, 20
23	19.8	1.13, s		12, 14, 18	11β, 12, 15β, 21
24	77.0	5.07, q (6.8)	26	17, 18, 25	
25	170.9				
26	18.2	1.39, d (6.8)	24	17	16
27	16.0	1.07, d (6.3)	20	20	3β
20-$CH_3\underline{C}O$	170.5				
20-$\underline{C}H_3CO$	22.0	2.03, s		20-$CH_3\underline{C}O$	
1'	171.5				
2'	43.5	2.38, dd (16.0, 3.4); 2.33, dd (16.0, 8.7)	3’	1’, 3’	
3'	64.4	4.11, m	2’, 4’		
4'	22.5	1.18, d (6.3)	3’	2’	

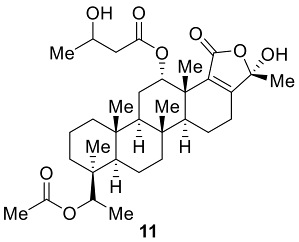

11

Table S11. ^{13}C (150 MHz) and ^{1}H (600 MHz) NMR data for compound **11**.

Position	δ_C	δ_H (J, Hz)	^{1}H-^{1}H COSY	HMBC	NOESY
1	40.7	1.66, m; 0.62, m	2		
2	18.0	1.45, m; 1.36, m	1, 3		
3	39.1	1.66, m; 1.02, m	2		3β(1.66)–27
4	39.4				
5	59.1	0.97, m			9
6	20.1	1.77, m; 1.44, m			
7	42.5	1.82, m; 0.93, m			
8	37.7				
9	54.4	1.16, m	11		5, 14
10	37.3				
11	21.4	1.96, m; 1.72, m	9, 12		11β(1.72)–22
12	74.9	5.69, dd (3.0, 2.4)	11	9	23
13	38.8				
14	51.8	1.47, m	15		9
15	16.6	1.90, m; 1.45, m	14, 16		
16	23.4	2.33, m; 2.28, m	15	14, 17, 18	16β(2.33)–26
17	162.9				
18	133.6				
19	23.3	0.97, s		4, 5, 20	
20	73.2	5.36, q (6.3)	27	5, 27, 20-CH$_3$<u>C</u>O	22
21	17.1	0.87, s		7, 9, 14	23
22	16.6	0.87, s		1, 5, 9	11β, 20
23	20.7	1.21, s		12, 13, 14, 18	12, 21
24	104.4				
25	169.0				
26	22.7	1.56, s		17, 24	16β
27	16.0	1.08, d (6.3)	20	4, 20	3β
20-CH$_3$<u>C</u>O	170.5				
20-<u>C</u>H$_3$CO	22.0	2.04, s		20-CH$_3$<u>C</u>O	
1'	169.1				
2'	44.1	2.55, dd (16.1, 3.0); 2.38, m	3’	1’, 3’	
3'	65.3	4.11, m	2’, 4’		
4'	22.8	1.23, d (6.3)	3’	2’, 3’	

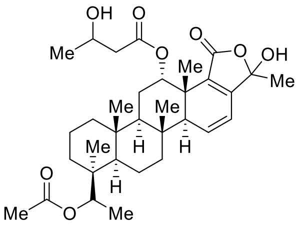

12

Table S12. ^{13}C (150 MHz) and ^{1}H (600 MHz) NMR data for compound **12**.

Position	δ_C	δ_H (J, Hz)	^{1}H-^{1}H COSY	HMBC	NOESY
1	40.5	1.67, m; 0.67, m	2	9	
2	18.0	1.47, m; 1.38, m	1, 3	10	2β(1.38)–20
3	39.1	1.67, m; 1.03, m	2		3β(1.67)–27
4	39.4				
5	59.1	0.99, m			
6	19.78	1.79, m; 1.47, m			6α(1.79)–19; 6β(1.47)–22
7	41.8	1.91, m; 0.93, m		5, 9	
8	37.3/37.2				
9	53.1/53.0	1.22, m	11		14
10	37.2				
11	21.5/21.4	2.07, m; 1.66, m	9, 12		11β(1.66)–22, 23
12	73.2/73.0	5.56/5.51, dd (3.0, 1.8)	11		23
13	40.2/40.0				
14	54.0/53.9	2.66/2.22, t (2.9)	15	15	9
15	138.9/138.7	6.38, m	14, 16	13, 14, 16, 17, 18	
16	118.4	6.28, dd (22.5, 3.2)/ 6.26, dd (22.5, 3.2)	15	14	
17	157.3				
18	131.0				
19	23.4	0.98/0.97, s		3, 5, 20	6α
20	73.3	5.36, q (6.2)	27	4, 5, 19, 27, 20-CH$_3$<u>C</u>O	2β, 22
21	18.7/18.6	0.99, s		7, 8, 14	
22	16.5	0.877/0.873, s		5, 9, 10	6β, 11β, 20
23	17.3/17.0	1.07/1.06, s		12, 13, 14, 18	11β, 12
24	103.32				
25	171.5				
26	24.8/23.6	1.67/1.63, s		17, 24	
27	16.0	1.10/1.09, d (6.3)	20	5, 20	3β
20-CH$_3$<u>C</u>O	170.4				
20-<u>C</u>H$_3$CO	22.0	2.05, s		20-CH$_3$<u>C</u>O	
1'	170.8				
2'	43.7/43.5	2.49, dd (16.9, 2.9) /2.39, m; 2.39, m	3’	1’, 3’	
3'	64.7/64.4	4.19/4.16, m	2’, 4’		
4'	22.6/22.5	1.23/1.21, d (6.3)	3’	2’	

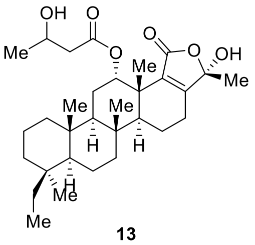

13

Table S13. ^{13}C (150 MHz) and ^{1}H (600 MHz) NMR data for compound **13**.

Position	δ_C	δ_H (*J*, Hz)	^1H-^1H COSY	HMBC	NOESY
1	40.4	1.51, m; 0.49, m	2		
2	18.3	1.40, m; 1.26, m	1		
3	36.7	1.58, m; 0.75, m		5	
4	36.3				
5	59.0	0.80, m			9
6	18.2	1.51, m; 1.40, m	7		6α (1.51)–19; 6β (1.40)–22
7	41.9	1.77, m; 0.94, m	6		
8	37.7				
9	54.0	1.10, m	11		5, 14
10	37.2				
11	21.4	1.88, m; 1.63, m	9, 12		
12	75.0	5.62, dd (3.0, 2.4)	11	9, 11, 14, 1’	23
13	38.9				
14	51.9	1.42, m			9
15	16.7	1.86, m; 1.42, m	16	17	
16	23.4	2.33, m; 2.12, m	15	17, 18	16β (2.33)–26
17	163.0				
18	133.7				
19	28.6	0.73, s		4, 5, 20	6α
20	24.6	1.45, m; 1.09, m	27		22
21	17.4	0.83, s		7, 8, 14	22, 23
22	16.9	0.77, s		1, 5, 9, 10	6β, 20, 21
23	20.7	1.13, s		13, 14, 18	12, 21
24	104.4				
25	163.0				
26	22.7	1.48, s		17, 24	16β
27	8.8	0.67, td (12.9, 4.3)	20	4, 20	
1'	169.1				
2'	44.1	2.47, dd (16.1, 3.0); 2.31, m	3’	1’	
3'	65.3	4.06, m	2’, 4’		
4'	22.8	1.16, d (6.4)	3’	2’, 3’	

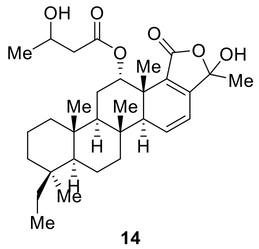

14

Table S14. ^{13}C (150 MHz) and ^{1}H (600 MHz) NMR data for compound **14**.

Position	δ_C	δ_H (*J*, Hz)	^1H-^1H COSY	HMBC	NOESY
1	40.0	1.58, m; 0.61, m	2		1α (0.61)–9; 1β (1.57)–11α (2.04)
2	18.1	1.46, m; 1.35, m	1, 3		
3	36.5	1.65, m; 0.84, m	2	5	
4	36.2				
5	58.8	0.89, m		19	9
6	17.7	1.58, m; 1.49, m			6α (1.58)–9, 19; 6β (1.49)–21, 22
7	41.0	1.93, m; 1.02, m		5, 9	
8	36.9				
9	52.6/52.5	1.22, m	11		1α, 5, 14
10	37.2/37.1				
11	21.3/21.2	2.04, m; 1.63, m	9, 12		11α-1β; 11β (1.65)–21, 22, 23
12	73.0	5.55/5.49, dd (3.0, 1.8)	11	9, 14	23
13	39.9				
14	53.9	2.68/2.62, t (3.0)	15	15, 16	9
15	138.8/138.7	6.40, dt (10.0, 2.0)/ 6.38, dt (10.0, 2.0)	14, 16	13, 14, 16, 17	
16	118.4/118.2	6.28, dd (25.0, 3.2)/ 6.27, dd (25.0, 3.2)	15	14, 18	
17	157.6/156.7				
18	130.7/130.6				
19	28.5	0.80/0.79, s		5	6α
20	24.5	1.52, m; 1.16, m	27	4, 19, 27	22
21	18.9/18.8	1.02, s		7, 8, 14	6β, 11β
22	16.6	0.839/0.834, s		1, 5, 9, 10	6β, 11β, 20
23	17.1/16.9	1.05/1.04, s		12, 13, 14, 18	11β, 12
24	103.5/103.4				
25	168.9/168.3				
26	24.6/23.5	1.65/1.62, s		17, 24	
27	8.6	0.74, t (7.5)	20	4, 20	
1'	171.4/170.7				
2'	43.6/43.4	2.52, dd (16.0, 3.2); 2.36, m	3’	1’, 3’	
3'	64.7/64.4	4.18/4.13, m	2’, 4’		
4'	22.5/22.3	1.22/1.20 d (6.3)	3’	2’, 3’	

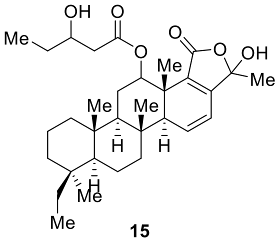

15

Table S15. ^{13}C (150 MHz) and ^{1}H (600 MHz) NMR data for compound **15**.

Position	δ_C	δ_H (J, Hz)	^1H-^1H COSY	HMBC	NOESY
1	40.2/40.1	1.58, m; 0.62, m	2		
2	18.3	1.46, m; 1.35, m	1		
3	36.7	1.66, m; 0.84, m			
4	36.3				
5	58.9	0.89, m			9
6	17.8	1.57, m; 1.49, m			6α(1.57)–19; 6β(1.49)–22
7	41.2/41.1	1.93, m; 1.02, m		5, 9	7α(1.02)–14; 7β(1.93)–21
8	37.1/37.0				
9	52.7/52.6	1.23, m	11		5, 14
10	37.3/37.2				
11	21.4	2.05, m; 1.65, m	9, 12		11β(1.59)–22
12	73.2/73.1	5.55/5.49, dd (2.3, 1.8)		9, 14, 1’	23
13	40.2/40.1				
14	54.0	2.69/2.63, t (3.0)	15	15, 16	7α, 9
15	139.0	6.41, dt (10.0, 2.0)/ 6.40, dt (10.0, 2.0)	14, 16	16, 17	
16	118.4/118.3	6.28, dd (24.8, 3.1)/ 6.27, dd (24.8, 3.1)	15	14, 15, 17, 18	
17	157.7/156.7				
18	130.9/130.8				
19	28.7/28.6	0.80, s		5, 20	6α
20	24.7	1.53, m; 1.16, m	27	4, 27	22
21	19.1/18.9	1.02, s		8, 14	7β
22	16.8/16.7	0.84, s		1, 5, 9	6β, 11β, 20
23	17.3/17.0	1.06/1.05, s		12, 13, 14, 18	12
24	103.5/103.4				
25	168.9/168.3				
26	24.8/23.5	1.66/1.62, s		17, 24	
27	8.8	0.67, t (6.9)	20	4, 20	
1'	171.6/170.9				
2'	41.9/41.8	2.50, dd (15.9, 2.9)/2.42, m; 2.41/2.35, m	3’	1’, 3’	
3'	69.9/69.7	3.90/3.85, m	2’, 4’		
4'	29.5/29.5	1.51/1.25, m	3’, 5’	3’	
5'	10.1	0.95, t (7.0)	4’	3’	

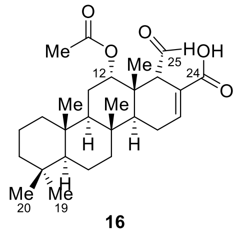

16

Table S16. ^{13}C (150 MHz) and ^{1}H (600 MHz) NMR data for compound **16**.

Position	δ_C	δ_H (*J*, Hz)	^{1}H-^{1}H COSY	HMBC	NOESY
1	39.7	1.50, m; 0.50, m			1α(0.50)–9
2	18.0	1.44, m; 1.25, m			
3	42.0	1.30, m; 1.04, m			
4	33.3				
5	56.4	0.78, m	6		
6	18.4	1.44, m; 1.25, m	5		
7	41.2	1.67, m; 1.02, m			
8	36.8				
9	52.4	1.18, m		12	1α, 14
10	37.8				
11	22.5	1.75, m; 1.62, m	12		11β(1.62)–21
12	76.9	4.80, dd (3.0, 2.4)	11	9, 14, 1'	23
13	41.9				
14	44.3	2.04, m	15	15	9, 25
15	24.7	2.42, m; 2.14, m	14, 16	14, 16, 17	15β(2.14)–21
16	145.8	7.30, dd (4.3, 3.3)	15	14, 18	
17	124.2				
18	58.7	3.07, dd (4.5, 1.1)	25	14, 16, 17, 24, 25	23
19	33.3	0.79, s		4, 20	
20	21.4	0.73, s		3, 4, 5	
21	16.4	0.85, s		9, 14	11β, 15β
22	16.3	0.74, s		1, 9, 10	
23	21.8	0.86, s		12, 13, 18	12, 18
24	169.6				
25	196.4	9.41, d (4.5)	18	18	14
1'	169.6				
2'	21.3	1.95, s		1'	

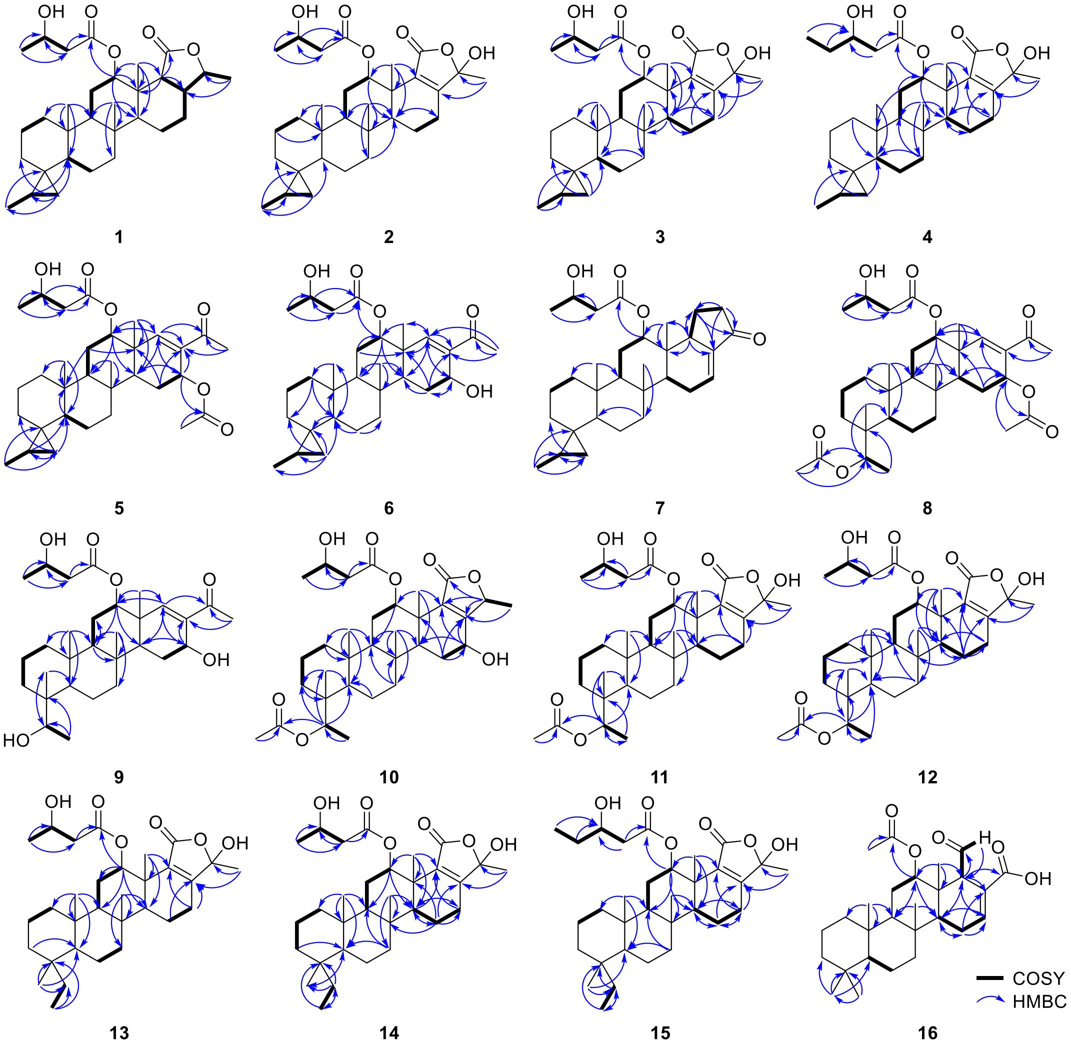

Figure S2. Key ^{1}H-^{1}H COSY and HMBC correlations of compounds **1-16**.

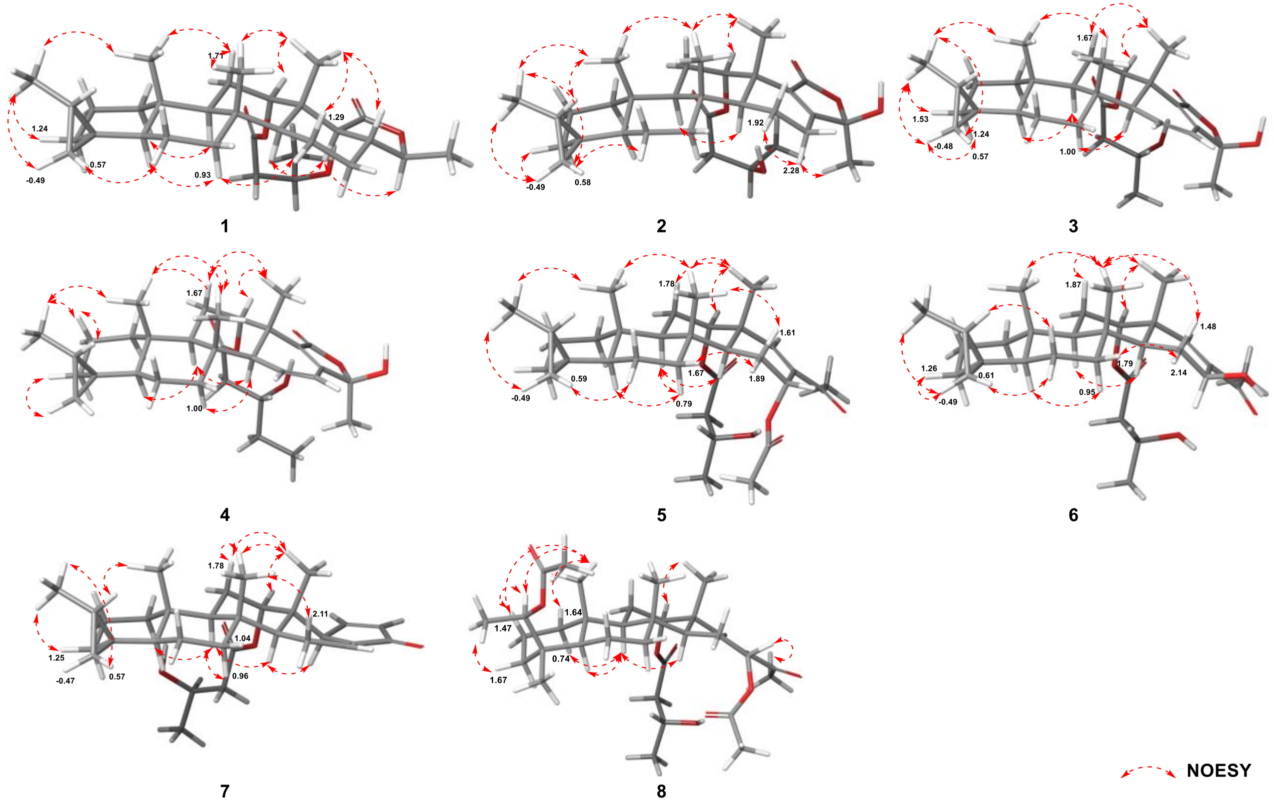

Figure S3. Key NOESY correlations of compounds **1-16** (continued).

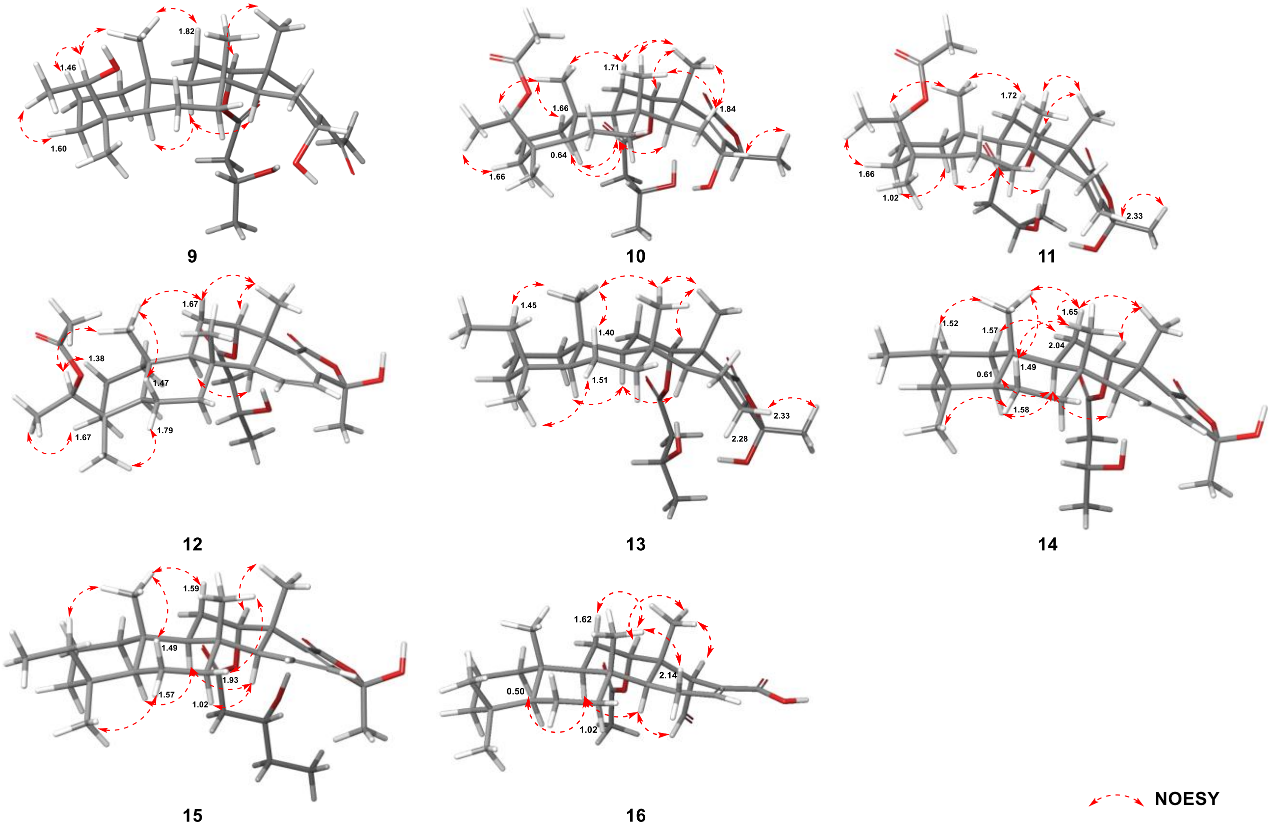

Figure S3. Key NOESY correlations of compounds **1-16**.

Table S17. ^{13}C chemical shifts of phyllactone D/E (**17**, **18**), 12-*epi*-phyllactone D/E (**15**) analyzed by Li et al[1], Andersen et al[2], and our experimental data.

	12β-epimer			12-*epi*-phyllactone D/E	
Position	Phyllactone D (**17**)	Phyllactone E (**18**)	Li et al[1]	Andersen et al[2]	**experimental**
#C	δ_C	δ_C	δ_C	δ_C	δ_C
1	40.1	40.5	40.2	40.0	40.2/40.1
2	17.9	17.9	18.1	18.2	18.3
3	36.8	36.8	36.8	36.6	36.7
4	36.3	36.3	36.1	36.1	36.3
5	58.8	58.8	58.6	58.6	58.9
6	18.3	18.3	17.7	17.8	17.8
7	42.2	41	40	41	41.2/41.1
8	37	37	37.3	37.2	37.1/37.0
**9**	**58.5**	**58.5**	**57**	**52.5**	**52.7/52.6**
10	37.6	37.6	36.6	36.8	37.3/37.2
**11**	**24.3**	**24.4**	**24.1**	**21.3**	**21.4**
**12**	**75.1**	**75.8**	**75.3**	**73.1**	**73.2/73.1**
13	42.4	41.1	42.2	40	40.2/40.1
**14**	**57.2**	**57**	**58.5**	**53.9**	**54.0**
15	139.6	139.3	139.7	138.8	139.0
16	119.6	119.3	119.4	118.1	118.4/118.3
17	158.6	158.2	158.2	156.4/157.2	157.7/156.7
18	132.1	132.1	132.2	131.1	130.9/130.8
19	28.9	28.7	28.5	28.5	28.7/28.6
20	24.7	24.7	24.5	24.5	24.7
21	16.8	16.8	18.8	16.7	19.1/18.9
22	18.9	18.9	16.6	18.9	16.8/16.7
**23**	**12.7**	**12.7**	**12.9**	**17**	**17.3/17.0**
24	103.1	103.1	102.7	103.2	103.5/103.4
25	168.6	167.9	168.2	unassigned	168.9/168.3
26	24.1	24.2	24.5	23.4/24.5	24.8/23.5
27	8.8	8.8	8.6	8.64	8.8
1'	173.5	172.9	172.8	171.1/171.3	171.6/170.9
2'	42.5	42.2	42	41.8	41.9/41.8
3'	69.9	69.7	69.7	69.7	69.9/69.7
4'	29.6	29.6	29.6	29.5	29.5/29.5
5'	10	10.1	10	9.9	10.1

[1] Lan, W. J.; Li, H. J. *Helv. Chim. Acta* **2007**, *90*, 1218–1222.
[2] Williams, D. E.; Hollander, I.; Feldberg, L.; Frommer, E.; Mallon, R.; Tahir, A.; van Soest, R.; Andersen, R. J. *J. Nat. Prod.* **2009**, *72*, 1106–1109.

Table S18. ^{1}H chemical shifts of phyllactone D/E (**17**, **18**), 12-*epi*-phyllactone D/E (**15**) analyzed by Li et al[1], Andersen et al[2], and our experimental data.

	12*β*-epimer			12-*epi*-phyllactone D/E	
Position	Phyllactone D (**17**)	Phyllactone E (**18**)	Li et al[1]	Andersen et al[2]	**experimental**
#H	δ_H	δ_H	δ_H	δ_H	δ_H
9			1.05-1.08, m	1.22, nd	1.23, m
11			ax: 1.65-1.71, m;<br>eq: 1.94, ddd (13.5, 3.0, 2.5)	2.05, nd;<br>1.63, nd	2.05, m;<br>1.65, m
12	5.22, dd (11.0, 4.0)	4.99, dd (11.0, 4.0)	5.19, dd (3.0, 2.5)	5.56/5.51, bs	5.58/5.51,<br>dd (2.3, 1.8)
14			2.19, d (3.5)	2.67/2.63, bs	2.69/2.63, t (3.0)
23	1.08, s	1.08, s	1.12/1.11, s	1.08, s	1.06/1.05, s

[1] Lan, W. J.; Li, H. J. *Helv. Chim. Acta* **2007**, *90*, 1218–1222.
[2] Williams, D. E.; Hollander, I.; Feldberg, L.; Frommer, E.; Mallon, R.; Tahir, A.; van Soest, R.; Andersen, R. J. *J. Nat. Prod.* **2009**, *72*, 1106–1109.

Table S19. ^{13}C chemical shifts of representative examples of 12α-epimers (**3**, **4**, **12**, **14**, **15**) and 12β-epimers (**17**, **18**, **20**[1], **21**[1]).

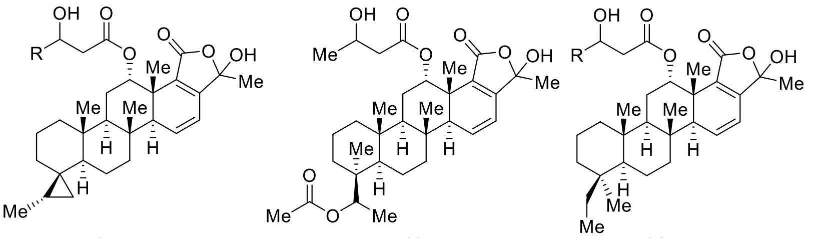

3 R = Me
4 R = Et
12
14 R = Me
15 R = Et

Position	**3**	**4**	**12**	**14**	**15**
9	50.7/50.6	50.7/50.6	53.1/53.0	52.6/52.5	52.7/52.6
11	21.5	21.5	21.5/21.4	21.3/21.2	21.4
12	73.2/73.1	73.2/73.1	73.2/73.0	73.0	73.2/73.1
14	54.1/54.0	54.1/54.0	54.0/53.9	53.9	54.0
23	17.3/17.1	17.3/17.1	17.3/17.0	17.1/16.9	17.3/17.0

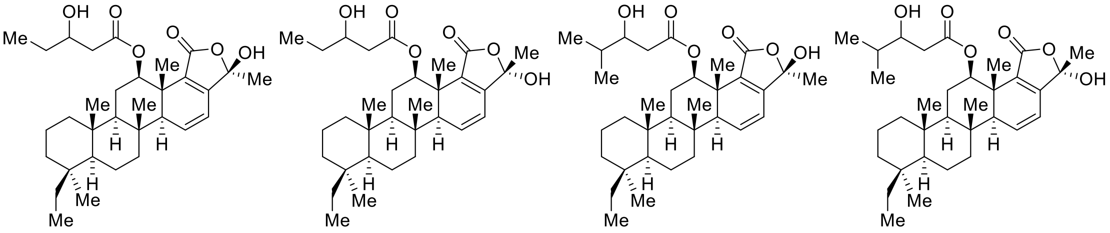

Phyllactone D (**17**) Phyllactone E (**18**) Phyllactone F (**20**) Phyllactone G (**21**)

Position	**17**	**18**	**20**	**21**
9	58.5	58.5	58.5	58.4
11	24.3	24.4	24.5	24.3
12	75.1	75.8	75.5	75.3
14	57.2	57.0	57.1	56.8
23	12.7	12.7	12.6	12.6

[1] Fu, X.; Zeng, L.; Su, J.; Pais, M.; Potier, P. *J. Nat. Prod.* **1993**, *56*, 1985–1988.

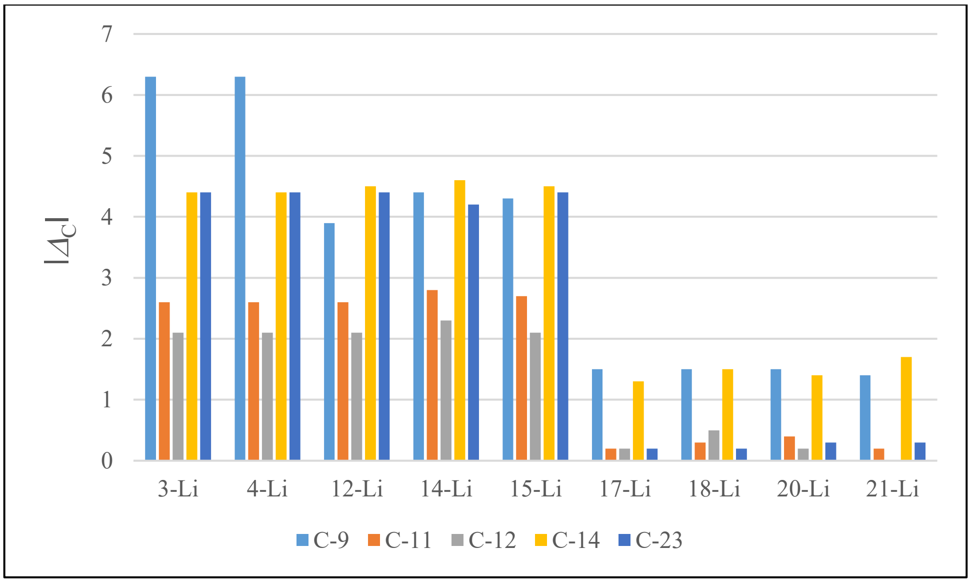

Figure S4. Comparison of |Δ_C| values at C-9, 11, 12, 14, 23 for 12α-epimers (**3**, **4**, **12**, **14**, **15**) and 12β-epimers (**17**, **18**, **20**, **21**) relative to those reported by Li et al.

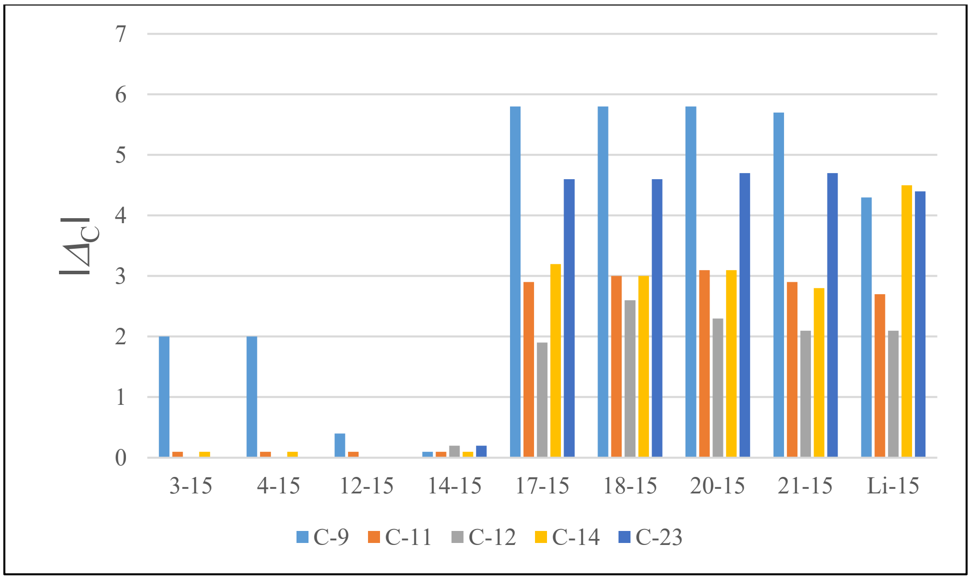

Figure S5. Comparison of |Δ_C| values at C-9, 11, 12, 14, 23 for 12α-epimers (**3**, **4**, **12**, **14**) and 12β-epimers (**17**, **18**, **20**, **21**, Li et al) relative to our experimental data for **15**.

II. Computational methods

1. Geometry optimization

All of the molecular mechanics calculations were performed on Macromodel software (Maestro Materials Science 3.7.013 based on Maestro Core 12.3.013, MMshare Version 4.9.013, Release 2020-1, Platform Windows-x64). All conformational searches were conducted using the Polak-Ribier Conjugate Gradient (PRCG) method, the mixed torsional/low-mode sampling method, and the Optimized Potentials for Liquid Simulation (OPLS3e) force field. Additionally, the searches were carried out in the gas phase with a 21.0 kJ/mol upper energy limit. The maximum number of steps was set as 1000 to find all of the possible conformers.

Quantum mechanical calculations were performed using Jaguar (version 10.7). The conformers within an energy threshold of 5.0 kJ/mol were used as input structures for DFT calculations employing the hybrid-B3LYP and the 6-3lG** basis set to output optimized structures. All optimization was performed “fine” grid density and “ultrafine” accuracy level.
